# An updated systematic review of radiomics in osteosarcoma: utilizing CLAIM to adapt the increasing trend of deep learning application in radiomics

**DOI:** 10.1186/s13244-022-01277-6

**Published:** 2022-08-20

**Authors:** Jingyu Zhong, Yangfan Hu, Guangcheng Zhang, Yue Xing, Defang Ding, Xiang Ge, Zhen Pan, Qingcheng Yang, Qian Yin, Huizhen Zhang, Huan Zhang, Weiwu Yao

**Affiliations:** 1 Department of Imaging, Tongren Hospital, Shanghai Jiao Tong University School of Medicine, No. 1111 Xianxia Road, Shanghai, 200336 China; 2 Department of Sports Medicine, Shanghai Jiao Tong University Affiliated Sixth People’s Hospital, No. 600 Yishan Road, Shanghai, 200233 China; 3 Department of Orthopedics, Shanghai Jiao Tong University Affiliated Sixth People’s Hospital, No. 600 Yishan Road, Shanghai, 200233 China; 4 Department of Pathology, Shanghai Jiao Tong University Affiliated Sixth People’s Hospital, No. 600 Yishan Road, Shanghai, 200233 China; 5 Department of Radiology, Ruijin Hospital, Shanghai Jiao Tong University School of Medicine, No. 197 Ruijin 2nd Road, Shanghai, 200025 China

**Keywords:** Osteosarcoma, Radiomics, Machine learning, Quality improvement, Systematic review

## Abstract

**Objective:**

To update the systematic review of radiomics in osteosarcoma.

**Methods:**

PubMed, Embase, Web of Science, China National Knowledge Infrastructure, and Wanfang Data were searched to identify articles on osteosarcoma radiomics until May 15, 2022. The studies were assessed by Radiomics Quality Score (RQS), Transparent Reporting of a multivariable prediction model for Individual Prognosis Or Diagnosis (TRIPOD) statement, Checklist for Artificial Intelligence in Medical Imaging (CLAIM), and modified Quality Assessment of Diagnostic Accuracy Studies (QUADAS-2) tool. The evidence supporting radiomics application for osteosarcoma was rated according to meta-analysis results.

**Results:**

Twenty-nine articles were included. The average of the ideal percentage of RQS, the TRIPOD adherence rate and the CLAIM adherence rate were 29.2%, 59.2%, and 63.7%, respectively. RQS identified a radiomics-specific issue of phantom study. TRIPOD addressed deficiency in blindness of assessment. CLAIM and TRIPOD both pointed out shortness in missing data handling and sample size or power calculation. CLAIM identified extra disadvantages in data de-identification and failure analysis. External validation and open science were emphasized by all the above three tools. The risk of bias and applicability concerns were mainly related to the index test. The meta-analysis of radiomics predicting neoadjuvant chemotherapy response by MRI presented a diagnostic odds ratio (95% confidence interval) of 28.83 (10.27–80.95) on testing datasets and was rated as weak evidence.

**Conclusions:**

The quality of osteosarcoma radiomics studies is insufficient. More investigation is needed before using radiomics to optimize osteosarcoma treatment. CLAIM is recommended to guide the design and reporting of radiomics research.

**Supplementary Information:**

The online version contains supplementary material available at 10.1186/s13244-022-01277-6.

## Key points

- The MRI-radiomics in predicting neoadjuvant chemotherapy response is supported by weak evidence.
- The quality of osteosarcoma radiomics studies has been improved recent two years.
- CLAIM can adapt the increasing trend of deep learning application in radiomics.

## Introduction

Osteosarcoma is the most common primary high-grade sarcoma of the skeleton, in which the tumor cells produce neoplastic bone [1]. Imaging is the key examination in the work-up of osteosarcoma management, from diagnosis, staging, treatment evaluation, to follow-up [1–3]. The diagnosis of osteosarcoma generally starts with X-ray radiography and is followed by CT for further evaluation. A contrast-enhanced MRI scan is useful in diagnosis completion and soft tissue involvement assessment and is usually the last step before biopsy of local disease. A chest CT scan is substantial for lung metastases detection. For patients with pathologically confirmed osteosarcoma, a whole-body PET examination has been recommended for initial staging rather than a bone scintigraphy nowadays. The treatment evaluation and follow-up imaging commonly include local CT and MRI scans and chest CT scan. In most cases, the current imaging approach with physical, laboratory, and histopathological examinations can guide clinicians to an appropriate curation plan, but there remain difficulties in differential diagnosis of osteosarcoma subtypes, prediction of response to treatment, and prognosis concerns including survival, recurrence, and lung metastasis [2, 3]. Radiomics, utilizing a plethora of strategies for extracting underlying information from medical images, has been used to overcome such challenges [4–7]. Radiomics models have been deemed as a promising approach for addressing clinical problems related to osteosarcoma patients, especially for predicting their response to neoadjuvant chemotherapy (NAC) [8].

Our preliminary search suggested that radiomics studies in osteosarcoma patients have doubled since the publication of the previous review [8], indicating necessity for updates on this rapidly developing field. It is unclear whether the radiomics study quality has improved in recent years. Next, the study quality and risk of bias of radiomics research on osteosarcoma have been only assessed by the Radiomics Quality Score (RQS) [7] and the modified Quality Assessment of Diagnostic Accuracy Studies (QUADAS-2) tool [9]. An additional evaluation using the Transparent Reporting of a multivariable prediction model for Individual Prognosis Or Diagnosis (TRIPOD) checklist [10] has been recommended to identify several significant items for reporting transparency of radiomics studies [11–14]. Further, RQS and TRIPOD may not be totally suitable for current radiomics studies, since recently developed deep radiomics applies convolutional neural networks to analyze these extracted features [15–18]. The Checklist for Artificial Intelligence in Medical Imaging (CLAIM) [19] has been demonstrated as a useful tool to improve design and reporting of deep learning research [20, 21]. It is potentially better for the evaluation of current radiomics studies with increasing application of deep learning. Finally, the level of evidence supporting the radiomics application in osteosarcoma has not been evaluated yet [22]. It is of importance to provide an overall evidence strength rating before translating radiomics into clinical practice [23, 24]. Therefore, we hypothesized that the publication of the previous review could improve the radiomics study quality in osteosarcoma, and that CLAIM is a better tool for current radiomics studies.

The aim of the present study is to provide an updated systematic review of radiomics in osteosarcoma with quality assessment and evidence-level rating and find out whether CLAIM can better identify disadvantages in current radiomics studies.

## Methods

### Protocol and registration

The updating of this systematic review was decided according to a three-step decision framework [25] and was conducted in the style of the Preferred Reporting Items for Systematic Reviews and Meta-Analyses (PRISMA) statement [26]. The review protocol (CRD42020175383) and updating information are present in Additional file 1: Note S1. The PRISMA checklist for current systematic review and meta-analysis is present as Additional file 2.

### Literature search and selection

An up-to-date literature search was performed via PubMed, Embase, Web of Science, China National Knowledge Infrastructure, and Wanfang Data until May 15, 2022, by two reviewers each with 4 years’ experience in radiology and radiomics research. Disagreements were solved by a review group consisting of radiologists, orthopedists, and pathologists with different levels of experience. All primary research assessing the role of radiomics in osteosarcoma treatment for diagnostic, prognostic, or predictive purposes was considered eligible for the current review. No publication period restrictions were applied, while only articles in English, Japanese, Chinese, German or French were available. The two reviewers screened the titles and abstracts after the removal of duplications and obtained the full-texts and their supplementary materials. The same reviewers determined their eligibility according to the inclusion and exclusion criteria. Other potentially eligible articles were identified from the reference lists of relevant articles and reviews. For uncertainties, the review group was consulted. The search and selection strategy is shown in Additional file 1: Note S2.

### Data extraction and quality assessment

We used a data collection sheet for bibliographical information, study characteristics, radiomics considerations, and model metrics (Additional file 1: Table S1) [8]. The eligible studies employed RQS [7], TRIPOD [10], CLAIM [19], and QUADAS-2 tools [9] (Additional file 1: Tables S2–S5). The RQS is a consensus list composed of sixteen items for methodological issues specific to radiomics studies and is later summarized to six key domains [12–14]. The TRIPOD statement provides a checklist consisting of thirty-seven items in twenty-two criteria aiming to promote transparency of prediction model studies and is recommended for identifying room of improvement in radiomics studies [12–14]. The CLAIM includes forty-two items in seven topics that should be viewed as a best practice to guide presentation of AI research [19]. The CLAIM has seldomly been employed for quality assessment of radiomics studies [20, 21]. However, we assumed that CLAIM is suitable for radiomics studies evaluation, as radiomics is a subset of AI application in medical imaging [15–18]. The QUADAS-2 tool was tailored to our review by modifying the signaling questions [8]. Two reviewers independently extracted the data and evaluated the studies. Disagreements were solved by discussion with the review group. Topics discussed are recorded in Additional file 1: Note S3.

### Data synthesis and analysis

The statistical analysis was performed with R language version 4.1.3 within RStudio version 1.4.1106 [27]. The RQS rating, the ideal percentage of RQS, and adherence rates of RQS, TRIPOD and CLIAM were calculated. In case a score of at least one point for each item was obtained without minus points, it was considered to have basic adherence, as those have been reported [12–14]. For example, if the item of validation in RQS obtained 2–5 points, it was considered as basic adherent, while it was regarded as without basic adherence when it was rated as -5 points. The QUADAS-2 assessment result was summarized. Pearson correlation test was used for the correlation analysis between the ideal percentage of RQS, the TRIPOD adherence rate and the CLAIM adherence rate. Subgroup analysis was performed to compare the ideal percentage of RQS, the TRIPOD adherence rate and the CLAIM adherence rate by journal type, first authorship, imaging modality, and publication period. A two-tailed *p* value < 0.05 indicated statistical significance, unless otherwise specified. Post hoc multiple comparisons were adjusted using the Bonferroni method. The detailed data analysis method is described in Additional file 1: Note S4.

The meta-analysis was performed using Stata software version 15.1 [28]. In the current systematic review, the role of MRI-driven radiomics in prediction of osteosarcoma patients’ response to NAC was addressed repeatedly. To present the true performance of the radiomics model, corresponding meta-analysis was conducted based on results of testing datasets. The two-by-two tables were directly extracted from the articles or reconstructed based on available data. The diagnostic odds ratio (DOR) with 95% confidence interval (CI) and corresponding p value were calculated using the random-effect model. Sensitivity, specificity, positive and negative likelihood ratio were estimated. A summary receiver operating characteristic (SROC) curve was drawn. The Cochran’s Q test and the Higgins I^2^ test were used for heterogeneity assessment. The funnel plot was drawn with Egger’s test and Begg’s test, and the Deeks’ funnel plot was constructed with Deeks’ funnel plot asymmetry test for publication bias. A two-tailed *p* value > 0.10 indicated a low publication bias. The trim and fill method was employed to estimate the number of missing studies. The level of evidence supporting the clinical application of radiomics in osteosarcoma was rated based on results of meta-analysis (Additional file 1: Table S6) [22].

## Results

### Literature search

**Fig. 1:**
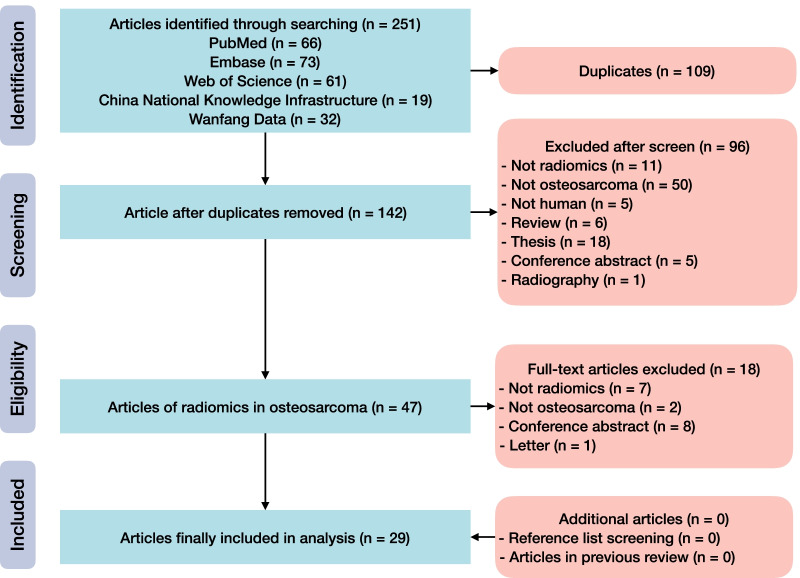
Flow diagram of study inclusion

The search yielded 251 records in total, in which 142 remained after removing duplicates. After screening the titles and abstracts, full-texts of 47 articles were retrieved and reviewed. Ultimately, 29 articles were included in this systematic review [29–57] (Fig. 1). No additional eligible study was identified through hand search of relevant reviews and reference lists of eligible articles.

### Study characteristics

**Fig. 2:**
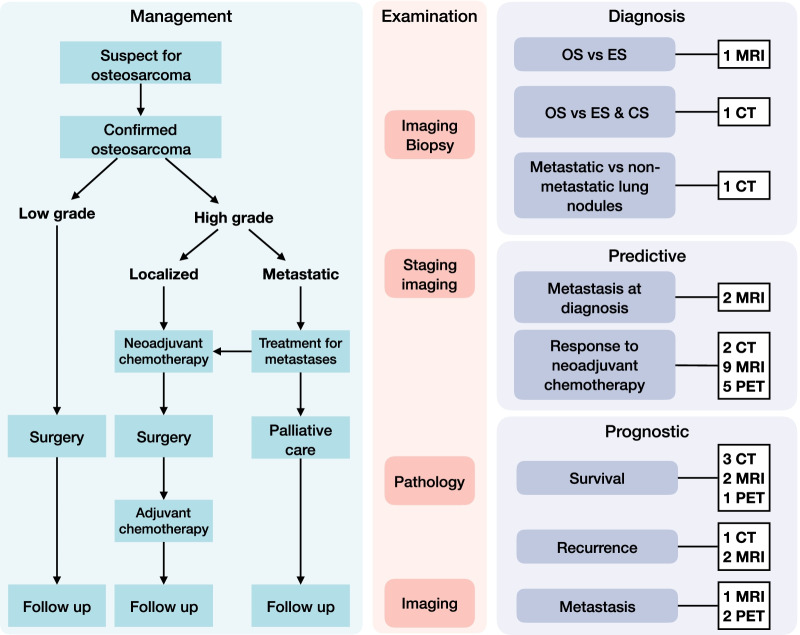
Imaging in osteosarcoma and radiomics study topics. Imaging examination is a routine in diagnosis and treatment decision in osteosarcoma. Radiomics has shown potential in personal precision medicine in this process. The study topics and number of radiomics studies in osteosarcoma with imaging modality were summarized. Note two studies built a prediction model for the response to NAC and a prognostic model for survival, respectively; and two studies built one prediction model for response to NAC a prognostic model for survival, respectively. This resulted in 33 radiomics models in 29 included studies. *OS* osteosarcoma, *ES* Ewing sarcoma, *CS* chondrosarcoma

Table 1 and Fig. 2 summarized the characteristics of 33 models described in the 29 included studies. The sample size of studies ranged from 17 to 191 patients with a median of 81 patients. More than a half of studies were published on non-imaging journals (55.2%), while the majority of first authorship belonged to radiologists (65.5%). The most utilized imaging modality was MRI (48.3%). Almost half of the models aimed to predict the response to NAC (48.5%), followed by prognostic models for survival (18.2%) and those for recurrence or metastasis (9.1%). Most models were validated within the same data with or without resampling (48.5%), while a limited number of models were externally validated (12.1%). The detailed characteristics of studies are present in Additional file 1: Tables S7–S10.

**Table 1:** Study characteristics

Study Characteristics	Data
Sample size, mean ± standard deviation, median (range)	86.6 ± 45.8, 81 (17–191)
Journal type, *n* (%)	*N* = 29
Imaging	13 (44.8)
Non-imaging	16 (55.2)
First authorship, *n* (%)	*N* = 29
Radiologist	19 (65.5)
Non-radiologist	10 (34.5)
Imaging modality, *n* (%)	*N* = 29
CT	9 (31.0)
MRI	14 (48.3)
PET	6 (20.7)
Biomarker, *n* (%)	*N* = 33
Diagnostic	3 (9.1)
Predictive	18 (54.5)
Prognostic	12 (36.4)
Model type, *n* (%)	*N* = 33
Type 1a: Developed model validated with exactly the same data	8 (24.2)
Type 1b: Developed model validated with resampling data	8 (24.2)
Type 2a: Developed model validated with randomly splitting data	12 (36.4)
Type 2b: Developed model validated with non-randomly splitting data	1 (3.0)
Type 3: Developed model validated with separate data	4 (12.1)
Type 4: Validation only	0 (0.0)

There were 33 radiomics models identified in 29 included studies. The model type was determined according to criteria in TRIPOD statement. *TRIPOD* Transparent Reporting of a multivariable prediction model for Individual Prognosis Or Diagnosis

### Study quality

**Fig. 3:**
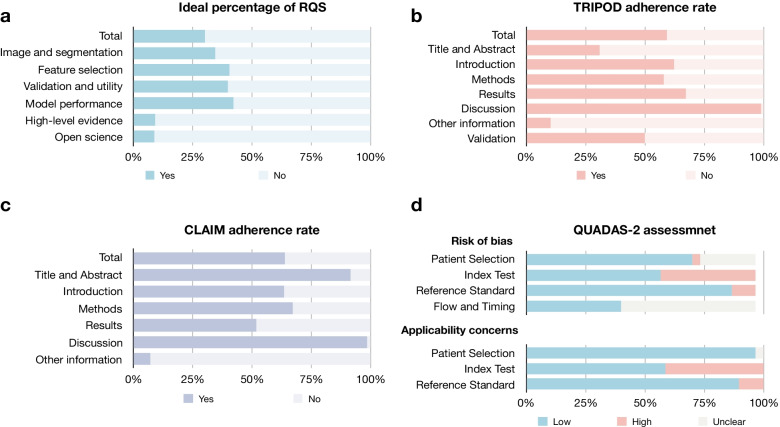
Quality assessment of included studies. **a** ideal percentage of RQS; **b** TRIPOD adherence rate; **c** CLAIM adherence rate; **d** QUADAS-2 assessment result

Figure 3 summarized the results of study quality evaluation. Table 2 showed that the median (range) of RQS for current osteosarcoma radiomics studies was 10 (3–18), with a percentage of the ideal score of 29.2% (305/1044) and the adherence rate of 44.6% (207/464). Tables 3 and 4 presented that the TRIPOD and CLAIM adherence rates were 59.2% (481/812) and 63.7% (961/1508), respectively. The risk of bias and applicability concerns were mainly related to the index test. The individual assessment for each study is present in Additional file 1: Tables S11–S14.

**Table 2:** RQS rating of included studies

16 items according to 6 key domains	Range	Median (range)	Percentage of ideal score, *n* (%)	Adherence rate, *n* (%)
Total 16 items	− 8–36	10 (3–18)	305/1044 (29.2)	207/464 (44.6)
Domain 1: protocol quality and stability in image and segmentation	0–5	2 (0–3)	50/145 (34.5)	50/116 (43.1)
Protocol quality	0–2	1 (0–1)	22/58 (37.9)	22/29 (75.9)
Multiple segmentations	0–1	1 (0–1)	20/29 (69.0)	20/29 (69.0)
Test–retest	0–1	0 (0–1)	8/29 (27.6)	8/29 (27.6)
Phantom study	0–1	0 (0–0)	0/29 (0.0)	0/29 (0.0)
Domain 2: feature selection and validation	− 8 to 8	5 (− 8 to 8)	94/232 (40.5)	49/58 (84.5)
Feature reduction or adjustment of multiple testing	− 3 to 3	3 (3–3)	69/87 (79.3)	26/29 (89.7)
Validation	− 5 to 5	2 (− 5 to 5)	25/145 (17.2)	23/29 (79.3)
Domain 3: biologic/clinical validation and utility	0–6	2 (0–5)	69/174 (39.7)	61/116 (52.6)
Non-radiomics features	0–1	1 (0–1)	18/29 (62.1)	18/29 (62.1)
Biologic correlations	0–1	1 (0–1)	27/29 (93.1)	27/29 (93.1)
Comparison to “gold standard”	0–2	0 (0 to 2)	16/58 (27.6)	8/29 (27.6)
Potential clinical utility	0–2	0 (0–1)	8/58 (13.8)	8/29 (27.6)
Domain 4: model performance index	0 to 5	2 (1–4)	61/145 (42.1)	35/87 (40.2)
Cut-off analysis	0–1	0 (0–0)	0/29 (0.0)	0/29 (0.0)
Discrimination statistics	0–2	2 (1–2)	49/58 (84.5)	29/29 (100.0)
Calibration statistics	0–2	0 (0–2)	12/58 (20.7)	6/29 (20.7)
Domain 5: high level of evidence	0–8	0 (0–7)	21/232 (9.1)	3/58 (5.2)
Prospective study	0–7	0 (0–7)	21/203 (10.3)	3/29 (10.3)
Cost-effectiveness analysis	0–1	0 (0–0)	0/29 (0.0)	0.29 (0.0)
Domain 6: open science and data	0–4	0 (0–2)	10/116 (8.6)	9/29 (31.0)

The ideal score was described as score and percentage of score to ideal score for each item. In the cases where a score of one point per item was obtained, the study was considered to have basic adherence to each item. The adherence rate was calculated as proportion of the number of articles with basic adherence to number of total articles

*RQS* Radiomics Quality Score

**Table 3:** TRIPOD adherence of included studies

37 Selected items in 22 criteria according to 7 sections (*N* = 29)	Study, *n* (%)
Overall (excluding items 5c, 11, 14b, 10c, 10e, 12, 13, 17, and 19a)	481/812 (59.2)
Section 1: Title and Abstract	18/58 (31.0)
1. Title—identify developing/validating a model, target population, and the outcome	2/29 (6.9)
2. Abstract—provide a summary of objectives, study design, setting, participants, sample size, predictors, outcome, statistical analysis, results, and conclusions	16/29 (55.2)
Section 2: Introduction	36/58 (62.1)
3a. Background—Explain the medical context and rationale for developing/validating the model	29/29 (100.0)
3b. Objective—Specify the objectives, including whether the study describes the development/validation of the model or both	7/29 (24.1)
Section 3: Methods	218/277 (57.8)
4a. Source of data—describe the study design or source of data (randomized trial, cohort, or registry data)	29/29 (100.0)
4b. Source of data—specify the key dates	29/29 (100.0)
5a. Participants—specify key elements of the study setting including number and location of centers	29/29 (100.0)
5b. Participants—describe eligibility criteria for participants (inclusion and exclusion criteria)	22/29 (75.9)
5c. Participants—give details of treatment received, if relevant (*N* = 25)	16/25 (64.0)
6a. Outcome—clearly define the outcome, including how and when assessed	27/29 (93.1)
6b. Outcome—report any actions to blind assessment of the outcome	3/29 (10.3)
7a. Predictors—clearly define all predictors, including how and when assessed	10/29 (34.5)
7b. Predictors—report any actions to blind assessment of predictors for the outcome and other predictors	4/29 (13.8)
8. Sample size—explain how the study size was arrived at	3/29 (10.3)
9. Missing data—describe how missing data were handled with details of any imputation method	6/29 (20.7)
10a. Statistical analysis methods—describe how predictors were handled	29/29 (100.0)
10b. Statistical analysis methods—specify type of model, all model-building procedures (any predictor selection), and method for internal validation	21/29 (72.4)
10d. Statistical analysis methods—specify all measures used to assess model performance and if relevant, to compare multiple models (discrimination and calibration)	6/29 (20.7)
11. Risk groups—provide details on how risk groups were created, if done (*N* = 0)	n/a
Section 4: Results	117/174 (67.2)
13a. Participants—describe the flow of participants, including the number of participants with and without the outcome. A diagram may be helpful	16/29 (55.2)
13b. Participants—describe the characteristics of the participants, including the number of participants with missing data for predictors and outcome	26/29 (89.7)
14a. Model development—specify the number of participants and outcome events in each analysis	23/29 (79.3)
14b. Model development—report the unadjusted association between each candidate predictor and outcome, if done (N = 5)	4/5 (80.0)
15a. Model specification—present the full prediction model to allow predictions for individuals (regression coefficients, intercept)	21/29 (72.4)
15b. Model specification—explain how to the use the prediction model (nomogram, calculator, etc.)	11/29 (37.9)
16. Model performance—report performance measures (with confidence intervals) for the prediction model	20/29 (69.0)
Section 5: Discussion	86/87 (98.9)
18. Limitations—Discuss any limitations of the study	28/29 (96.6)
19b. Interpretation—Give an overall interpretation of the results	29/29 (100.0)
20. Implications—Discuss the potential clinical use of the model and implications for future research	29/29 (100.0)
Section 6: Other information	6/58 (10.3)
21. Supplementary information—provide information about the availability of supplementary resources, such as study	0/29 (0.0)
22. Funding—give the source of funding and the role of the funders for the present study	6/29 (20.7))
Section 7: Validation for Model type 2a, 2b, 3, and 4 (N = 16)	32/64 (50.0)
10c. Statistical analysis methods—describe how the predictions were calculated	15/16 (93.8)
10e. Statistical analysis methods—describe any model updating (recalibration), if done (N = 0)	n/a
12. Development versus validation—Identify any differences from the development data in setting, eligibility criteria, outcome, and predictors	10/16 (62.5)
13c. Participants (for validation)—show a comparison with the development data of the distribution of important variables	2/16 (12.5)
17. Model updating—report the results from any model updating, if done (N = 0)	n/a
19a. Interpretation (for validation)—discuss the results with reference to performance in the development data and any other validation data	5/16 (31.3)

In the cases where a score of one point per item was obtained, the study was considered to have basic adherence to each item. The adherence rate was calculated as proportion of the number of articles with basic adherence to number of total articles. During the calculation, the “if done” or “if relevant” items (5c, 11, and 14b) and validation items (10c, 10e, 12, 13, 17, and 19a) were excluded from both the denominator and numerator

*TRIPOD* Transparent Reporting of a multivariable prediction model for Individual Prognosis Or Diagnosis, *n/a* not applicable

**Table 4:** CLAIM adherence of included studies

CLAIM items (*N* = 29)	Study, *n *(%)
Overall (excluding item 27)	961/1508 (63.7)
Section 1: Title and Abstract	53/58 (91.4)
1. Title or abstract—Identification as a study of AI methodology	29/29 (100.0)
2. Abstract—Structured summary of study design, methods, results, and conclusions	24/29 (82.8)
Section 2: Introduction	55/87 (63.2)
3. Background—scientific and clinical background, including the intended use and clinical role of the AI approach	29/29 (100.0)
4a. Study objective	22/29 (75.9)
4b. Study hypothesis	4/29 (13.8)
Section 3: Methods	700/1044 (67.0)
5. Study design—Prospective or retrospective study	29/29 (100.0)
6. Study design—Study goal, such as model creation, exploratory study, feasibility study, non-inferiority trial	29/29 (100.0)
7a. Data—Data source	29/29 (100.0)
7b. Data—Data collection institutions	29/29 (100.0)
7c. Data—Imaging equipment vendors	25/29 (86.2)
7d. Data—Image acquisition parameters	22/29 (75.9)
7e. Data—Institutional review board approval	28/29 (96.6)
7f. Data—Participant consent	24/29 (82.8)
8. Data—Eligibility criteria	22/29 (75.9)
9. Data—Data pre-processing steps	20/29 (69.0)
10. Data—Selection of data subsets (segmentation of ROI in radiomics studies)	26/29 (89.7)
11. Data—Definitions of data elements, with references to Common Data Elements	29/29 (100.0)
12, Data—De-identification methods	3/29 (10.3)
13. Data—How missing data were handled	6/29 (20.7)
14. Ground truth—Definition of ground truth reference standard, in sufficient detail to allow replication	27/29 (93.1)
15a. Ground truth—Rationale for choosing the reference standard (if alternatives exist)	0/29 (0.0)
15b. Ground truth—Definitive ground truth	29/29 (100.0)
16. Ground truth—Manual image annotation	17/29 (586)
17. Ground truth—Image annotation tools and software	10/29 (34.5)
18. Ground truth—Measurement of inter- and intra-rater variability; methods to mitigate variability and/or resolve discrepancies	9/29 (31.0)
19a. Data Partitions—Intended sample size and how it was determined	29/29 (100.0)
19b. Data Partitions—Provided power calculation	4/29 (13.8)
19c. Data Partitions—Distinct study participants	23/29 (79.3)
20. Data Partitions—How data were assigned to partitions; specify proportions	22/29 (75.9)
21. Data Partitions—Level at which partitions are disjoint (e.g., image, study, patient, institution)	22/29 (75.9)
22a. Model—Provided reproducible model description	21/29 (72.4)
22b. Model—Provided source code	0/29 (0.0)
23. Model—Software libraries, frameworks, and packages	20/29 (69.0)
24. Model—Initialization of model parameters (e.g., randomization, transfer learning)	23/29 (79.3)
25. Training—Details of training approach, including data augmentation, hyperparameters, number of models trained	16/29 (55.2)
26. Training—Method of selecting the final model	21/29 (72.4)
27. Training—Ensembling techniques, if applicable (*N* = 14)	8/14 (57.1)
28. Evaluation—Metrics of model performance	29/29 (100.0)
29. Evaluation—Statistical measures of significance and uncertainty (e.g., confidence intervals)	20/29 (69.0)
30. Evaluation—Robustness or sensitivity analysis	10/29 (34.5)
31. Evaluation—Methods for explainability or interpretability (e.g., saliency maps), and how they were validated	11/29 (37.9)
32. Evaluation—Validation or testing on external data	16/29 (55.2)
Section 4: Results	90/174 (51.7)
33. Data—Flow of participants or cases, using a diagram to indicate inclusion and exclusion	16/29 (55.2)
34. Data—Demographic and clinical characteristics of cases in each partition	25/29 (86.2)
35a. Model performance—Test performance	16/29 (55.2)
35b. Model performance—Benchmark of performance	8/29 (27.6)
36. Model performance—Estimates of diagnostic accuracy and their precision (such as 95% confidence intervals)	20/29 (69.0)
37. Model performance—Failure analysis of incorrectly classified cases	5/29 (17.2)
Section 5: Discussion	57/58 (98.3)
38. Study limitations, including potential bias, statistical uncertainty, and generalizability	28/29 (96.6)
39. Implications for practice, including the intended use and/or clinical role	29/29 (100.0)
Section 6: Other information	6/87 (6.9)
40. Registration number and name of registry	0/29 (0.0)
41. Where the full study protocol can be accessed	0/29 (0.0)
42. Sources of funding and other support; role of funders	6/29 (20.7)

*CLAIM* Checklist for Artificial Intelligence in Medical Imaging. In the cases where a score of one point per item was obtained, the study was considered to have basic adherence to each item. The adherence rate was calculated as proportion of the number of articles with basic adherence to number of total articles. During the calculation, the “if applicable” item (27) was excluded from both the denominator and numerator

RQS addressed a radiomics-specific issue of phantom study (0.0%) and the deficiency in cut-off analysis (0.0%) and cost-effectiveness analysis (0.0%). TRIPOD emphasized the shortness in reporting title (6.9%), blindness of assessment for outcomes and predictors (10.3%; 13.7%), and stating study objective in introduction (24.1%). Both RQS and CLAIM indicated a low percentage of comparing the model with the benchmark (27.6%; 27.6%), while both TRIPOD and CLAIM pointed out the disadvantages in sample size or power calculation (10.3%; 13.7%), and missing data handling (20.7%; 20.7%). CLAIM identified extra lacking in reporting in data de-identification (10.3%), stating study hypothesis in introduction (13.8%), and failure analysis (17.2%). All the above three tools emphasized the validation (25/145, 17.2%; 32/64, 50.0%; 16/29, 55.2%) and open science or additional information (10/116, 8.6%; 6/58, 10.3%; 6/87, 6.9%). The correlation between RQS and TRIPOD (*r* = 0.7498, *p* < 0.001) was moderate, while that between TRIPOD and CLAIM (*r* = 0.9004, *p* < 0.001) and that between RQS and CLAIM (*r* = 0.8158, *p* < 0.001) were high (Additional file 1: Fig. S1).

**Fig. 4:**
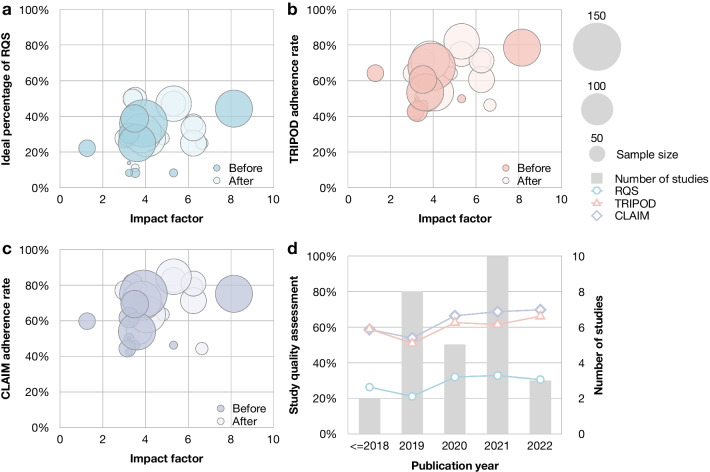
Quality evaluation with impact factor, sample size, and publication year. Swam plots of (**a**) ideal percentage of RQS, (**b**) TRIPOD adherence rate, and (**c**) CLAIM adherence rate with impact factor and sample size. The diameter of bubbles indicates the sample size of studies. The lighter color indicates the studies after the publication of previous review; the darker color indicates those before its publication. Notice one study published on journals without impact factor was excluded. **d** Bar plot depicting the number of studies, and line plots presenting ideal percentage of RQS, TRIPOD adherence rate, and CLAIM adherence rate of radiomics studies on osteosarcoma over the years

Figure 4 presents results of study quality evaluation with impact factor, sample size, and publication year. We compared the quality of studies published before and after the previous review and found that the ideal percentage of RQS (22.7% vs 33.8%, *p* = 0.020), the TRIPOD adherence rate (53.6% vs 63.4%, *p* = 0.026), and the CLAIM adherence rate (56.1% vs 69.1%, *p* = 0.007) have all been improved (Additional file 1: Table S15 and Additional file 1: Fig. S2). Subgroup analysis also found that imaging modalities utilized in studies have influence on TRIPOD and CLAIM adherence rates (*p* = 0.002, *p* = 0.004). The journal type and first authorship did not significantly influence study quality (both *p* > 0.05).

### Meta-analysis

**Fig. 5:**
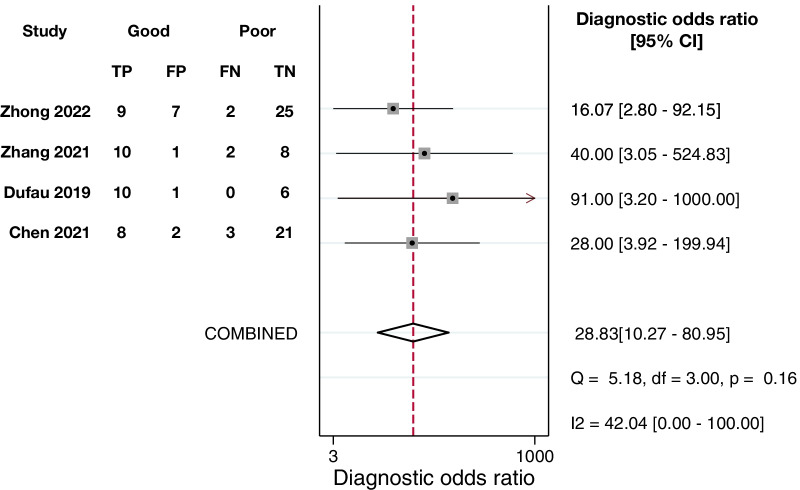
Forest plots of diagnostic odds ratios. The performance of radiomics in prediction of NAC response in osteosarcoma patients based on testing datasets. *TP* pathological good responders predicted as good responders, *FP* pathological poor responders predicted as good responders, *FN* pathological good responders predicted as poor responders, *TN* pathological poor responders predicted as poor responders

The meta-analysis of radiomics predicting NAC response by MRI presented a DOR of 28.83 (95%CI 10.27–80.95) on testing datasets of 115 osteosarcoma patients in total [35, 39, 55, 57] (Fig. 5). The corresponding metrics indicates a dramatic performance (Additional file 1: Figs. S3–S7). The Cochran’s Q test (*Q* = 5.18, *p* = 0.160) and Higgins *I*-square statistic (*I*^2^ = 42.04%) indicated that the heterogeneity was moderate. The funnel plot with Egger’s test (*p* = 0.035) and Begg’s test (*p* = 0.089) and the Deeks' funnel plot with Deeks’ asymmetry test (*p* = 0.069) revealed that the likelihood of publication bias was high (Additional file 1: Figs. S8–S9). The trim and fill analysis estimated that two studies were missing (Additional file 1: Fig. S10). However, the adjusted DOR was 20.53 (95%CI 7.80–54.06; *p* < 0.001). The level of evidence supporting the application of radiomics in predicting NAC response by MRI is rated as weak (Table 5). All meta-analyzed data are presented in Additional file 1: Table S16.

**Table 5:** The prediction performance of radiomics for NAC response in osteosarcoma patients

Clinical question	MRI-driven radiomics prediction model for NAC response in osteosarcoma patients
Number of studies	4
Good responder/sample size	44/115
Pooled analysis	
DOR (95%CI)	28.83 (10.27–80.95)
*p* value for DOR	*p* < 0.001
Sensitivity (95% CI)	0.84 (0.70–0.92)
Specificity (95% CI)	0.85 (0.74–0.91)
PLR (95% CI)	5.43 (3.11–9.49)
NLR (95% CI)	0.19 (0.09–0.37)
AUC (95% CI)	0.91 (0.88–0.93)
Heterogeneity	
Higgins *I*^2^ test	*I*^2^ = 42.04%
Cochran’s *Q* test	*Q* = 5.18, *p* = 0.160
Publication bias	
Egger’s test	*p* = 0.035
Begg’s test	*p* = 0.089
Deeks’ test	*p* = 0.069
Trim and fill method	
Number of missing studies	2
Adjusted DOR (95%CI)	20.53 (7.80–54.06)
*p* value for adjusted DOR	*p* < 0.001
Level of Evidence	Weak

*AUC* area under curve, *CI* confidential interval, *DOR* diagnostic odds ratio, *NAC* neoadjuvant chemotherapy, *NLR* negative likelihood ratio, *n/a* not applicable, *PLR* positive likelihood ratio

## Discussion

We provided an updated systematic review on osteosarcoma radiomics. Although the overall methodological and reporting quality of included studies was still suboptimal, it has improved after the publication of the previous review. The evidence supporting MRI-driven radiomics to predict NAC response in osteosarcoma has been rated as weak based on meta-analysis of testing data. CLAIM has shown unique ability in capturing deficiency in radiomics studies with deep learning.

In the previous review, the most frequently investigated question was whether radiomics could predict the NAC response [8], and it is still the most attractive topic nowadays in osteosarcoma radiomics [29–32, 35, 38–44, 49, 53, 55, 57]. The current review identified two studies each for differential diagnosis [37, 54], for metastasis at diagnosis [46, 47], and for early recurrence [33, 34] of osteosarcoma, while none of the previous twelve studies touched upon these topics. These achievements cover the routine for osteosarcoma and have potential in aiding clinicians to improve their treatment decision. MRI is currently the most frequently utilized imaging modality, and CT has exceeded PET to become the second. In terms of MRI techniques, T1 mapping and dynamic contrast-enhanced MRI have been introduced into osteosarcoma radiomics studies [31, 55]. However, whether these advanced techniques allow radiomics to better answer the clinical questions has not been fully investigated. Although most of studies segmented ROIs manually, two studies and one study, respectively, employed the region growing method based on the threshold of SUV [40, 42] and a deep learning nnU-Net [57] for automatic segmentation. These approaches may liberate radiologists from time-consuming segmentation workloads and potentially make osteosarcoma radiomics an automatic pipeline for clinical use. In addition to segmentation, deep learning models have been compared with radiomics models and showed better performance in predicting NAC response and metastasis [41, 42], and the performance of radiomics models improved when incorporating deep learning features [50]. The application of deep learning has not been detected by the previous review, but currently more studies used deep learning to further mine information in images. More studies tested their model using datasets from other institutions [33–35, 45] or splitting testing datasets [39, 44, 46–48, 50–57] to show the true performance of their models, while none of the studies in the previous review has been externally validated. The improvements in validation settings allow us to meta-analyze the performance of radiomics for prediction of NAC response based on testing datasets. The pooled DOR is lower than that in the previous review (28.83 vs 43.68), but result of the present review is more robust and interpretable [23]. We only included MRI-driven radiomics models which have been evaluated on testing datasets [35, 39, 55, 57], while the previous meta-analysis was carried out based on any imaging modality or dataset.

Study quality has improved since the publication of the previous review. However, the overall study quality is suboptimal. RQS and TRIPOD have identified disadvantages in phantom study, cut-off analysis, cost-effectiveness analysis, blindness of assessment, sample size calculation, and missing data handling, which have been repeatedly addressed [8, 12–14]. The previous review only employed RQS for quality assessment. RQS was a specialized tool proposed to help the radiomics community assess the quality and value of a radiomics study. However, RQS was tailored on hand‐crafted features. As deep learning is gaining momentum, the current version of RQS may not capture the strengths and weaknesses of deep learning radiomics studies correctly [58]. TRIPOD is a similar example that aimed to promote transparency reporting of diagnostic accuracy model studies and has been recommended to identify room for improvements in radiomics studies [11]. Nevertheless, the current version of TRIPOD may not capture some unique challenges with machine learning or AI application [59]. In contrast, CLAIM captured unique shortness in our review, such as data de-identification and failure analysis. CLAIM has been employed as a useful tool for quality evaluation in deep learning studies [20, 21], and our review demonstrated the feasibility of CLAIM in radiomics studies. We further confirmed that CLAIM can serve as a better review and study design guideline in radiomics studies. CLAIM may guide the update of TRIPOD and RQS, because it not only includes general reporting criteria, but also allows extra distinguishment of unique shortness in deep learning. CLAIM may even replace RQS and TRIPOD, considering the overlapping items and high correlation between these tools. The researchers are still reticent in publishing the RQS and TRIPOD for their radiomics studies [58]. Only one study in our review included RQS, TRIPOD and CLAIM as supplementary materials [57].

Our review has several limitations. First, our review focused on osteosarcoma radiomics studies. The conclusion should be interpreted with caution when expanded to other diseases. However, it provided insights for the design and reporting radiomics studies. Second, our study only included AI research applying the radiomics approach, but overlooked those conducted with only deep learning for segmentation [60–62] or modeling [63]. However, the secondary aim of our study is to find out whether CLAIM can better identify disadvantages in radiomics studies than the currently recommended RQS and TRIPOD. As CLAIM is suitable for both radiomics and deep learning radiomics studies, future review is encouraged to carry out without the restriction of radiomics. Third, it has not been investigated in our review how to weigh each item in CLAIM. The previous reviews have created subitems for some evaluations [20] and weighed them as equal [21]. We treated each subitem as equal, but it is necessary to find out whether it is appropriate. Fourth, we did not employ more specific tools for evaluation, because they are not suitable or are currently under development [59, 64–68]. The review may benefit from the increasing study reporting guidelines for clinical studies using AI in healthcare, because they pay extra attention to additional factors which do not neatly conform to traditional reporting guidelines, especially details relating to technical algorithm development [69]. The Image Biomarkers Standardization Initiative (IBSI) guideline is another potential eligible checklist for quality elevation [70]. However, we did not apply it as previous reviews [14, 24], since this radiomics-specific checklist may not suitable for deep learning studies. Finally, due to the heterogeneity and limited numbers of studies, we only rated the evidence level of radiomics in prediction of NAC response. Further investigation is needed to lay a more robust scientific basis for translating the radiomics approach to a clinical useful tool [23, 24].

In conclusion, the quality of radiomics studies in osteosarcoma has improved in recent years, but is still suboptimal. MRI-driven radiomics for prediction of NAC response in osteosarcoma is rated as weak evidence according to meta-analysis of testing datasets, calling for more high-quality studies to promote radiomics application in osteosarcoma. CLAIM can better identify disadvantages in radiomics studies and therefore is recommended for future evaluation of AI studies including radiomics.

## Supplementary Information

**Additional file 1**. **Note S1**: Review protocol. **Note S2**: Search strategy and study selection. **Note S3**: Consensus reached during data extraction and quality assessment. **Note S4**: Data synthesis and analysis methods. **Table S1**: Data extraction sheet. **Table S2**: RQS elements according to six key domains. **Table S3**: TRIPOD reporting completeness checklist. **Table S4**: CLAIM for authors and reviewers. **Table S5**: QUADAS-2 tool for risk of bias and concern on application. **Table S6**: Category of five levels of supporting evidence of meta-analyzes. **Table S7**: Study characteristics of included studies. **Table S8**: PICOT of included studies. **Table S9**: Radiomics methodological issue of included studies. **Table S10**: Model presentation and performance metrics of included studies. **Table S11**: RQS rating per study. **Table S12**: TRIPOD adherence per study. **Table S13**: CLAIM adherence per study. **Table S14**: QUADAS-2 assessment per study. **Table S15**: Subgroup analysis of study quality according to study characteristics. **Table S16**: Model metrics of studies included in meta-analysis. **Figure S1**: Correlation between quality evaluation tools. **Figure S2**: Subgroup analysis of quality evaluation results. **Figure S3**: Forrest plot of pooled sensitivity. **Figure S4**: Forrest plot of pooled specificity. **Figure S5**: Forrest plot of pooled positive likelihood ratio. **Figure S6**: Forrest plot of pooled negative likelihood ratio. **Figure S7**: SROC curve of the model performance. **Figure S8**: Funnel plot of studies included in meta-analysis. **Figure S9**: Deeks' funnel plot of studies included in meta-analysis. **Figure S10**: Trim and fill analysis of studies included in meta-analysis.

**Additional file 2**. PRISMA 2020 checklist.

## Data Availability

All data generated or analyzed during this study are included in this published article and its supplementary information files.

## Abbreviations

AI: Artificial intelligence
CI: Confidence interval
CLAIM: Checklist for Artificial Intelligence in Medical Imaging
DOR: Diagnostic odds ratio
NAC: Neoadjuvant chemotherapy
QUADAS-2: Modified Quality Assessment of Diagnostic Accuracy Studies
RQS: Radiomics Quality Score
SROC: Summary receiver operating characteristic
TRIPOD: Transparent Reporting of a multivariable prediction model for Individual Prognosis or Diagnosis
