# Supplementary material for: An updated systematic review of radiomics in osteosarcoma: utilizing CLAIM to adapt the increasing trend of deep learning application in radiomics
Source: Insights Imaging. 2022 Aug 20;13:138. doi: 10.1186/s13244-022-01277-6 (PMC9392674; doi:10.1186/s13244-022-01277-6)

ELECTRONIC SUPPLEMENTARY MATERIAL

An updated systematic review of radiomics in osteosarcoma: utilizing CLAIM to adapt the increasing trend of deep learning application in radiomics

List of Supplementary Materials

Supplementary Note S1 Review protocol
Supplementary Note S2 Search strategy and study selection
Supplementary Note S3 Consensus reached during data extraction and quality assessment
Supplementary Note S4 Data synthesis and analysis methods

Supplementary Table S1 Data extraction sheet
Supplementary Table S2 RQS elements according to six key domains
Supplementary Table S3 TRIPOD reporting completeness checklist
Supplementary Table S4 CLAIM for authors and reviewers
Supplementary Table S5 QUADAS-2 tool for risk of bias and concern on application
Supplementary Table S6 Category of five levels of supporting evidence of meta-analyzes
Supplementary Table S7 Study characteristics of included studies
Supplementary Table S8 PICOT of included studies
Supplementary Table S9 Radiomics methodological issue of included studies
Supplementary Table S10 Model presentation and performance metrics of included studies
Supplementary Table S11 RQS rating per study
Supplementary Table S12 TRIPOD adherence per study
Supplementary Table S13 CLAIM adherence per study
Supplementary Table S14 QUADAS-2 assessment per study
Supplementary Table S15 Subgroup analysis of study quality according to study characteristics
Supplementary Table S16 Model metrics of studies included in meta-analysis

Supplementary Figure S1 Correlation between quality evaluation tools
Supplementary Figure S2 Subgroup analysis of quality evaluation results
Supplementary Figure S3 Forrest plot of pooled sensitivity
Supplementary Figure S4 Forrest plot of pooled specificity
Supplementary Figure S5 Forrest plot of pooled positive likelihood ratio
Supplementary Figure S6 Forrest plot of pooled negative likelihood ratio
Supplementary Figure S7 SROC curve of the model performance
Supplementary Figure S8 Funnel plot of studies included in meta-analysis
Supplementary Figure S9 Deeks funnel plot of studies included in meta-analysis
Supplementary Figure S10 Trim and fill analysis of studies included in meta-analysis

Supplementary Note S1 Review protocol

PROSPERO registration

ROSPERO ID: CRD42020175383
Automatically published: 14 Jul 2020
Submit to PROSPERO: 26 Mar 2020
First draft: 10 Mar 2020
Last edited: 26 Mar 2020
Update started: 01 May 2022

Review question
This systematic review assesses the quality of the literature published on radiomics or texture analysis in CT, MRI, PET/CT or PET/MR of osteosarcoma in humans and identifies challenges impeding the clinical translation of proposed models for stratification of tumor, prediction of response to therapy or prognosis.

Searches
Primary publications concerning radiomics or image texture analysis of CT, MRI, PET/CT or PET/MR in patients with osteosarcoma will be included in this review. Electronic databases including PubMed, EMBASE and Web of Science will be searched. Literature search strategies will be developed using medical subject headings (MeSH) and derived words, including radiomics, textural analysis, CT, MR, PET, osteosarcoma, etc. No restriction will be made regarding publication period. The search strategy will include only terms relating to the review question. Publications must be available in English, Japanese, Chinese, German or French.

To ensure literature saturation, we will check the reference lists of included studies or relevant reviews identified through the search. As relevant studies are identified, reviewers will check for additional relevant cited and citing articles.

Types of study to be included
This systematic review will include primary research assessing the role of texture analysis in patients with osteosarcoma. Studies may be prospective or retrospective and randomized or non-randomized and shall investigate the use of texture analysis for diagnostic, prognostic or predictive purposes on cross-sectional imaging of human patients with osteosarcoma. The reference lists of included studies were screened for additional potentially eligible articles. However, reviews, technical reports, letters to editors, comments to published studies, conference proceedings, case reports and brief communications, and studies with insufficient information for assessing the methodological quality will be excluded.

Condition or domain being studied
Osteosarcoma is the most common primary high-grade sarcoma of the skeleton. Primary osteosarcoma arises in any bone, but originate most frequently surrounding the knee. It has a bimodal age distribution with most cases developing between the ages of 10–14 years and a second smaller peak in older adults aged 40 years. Although aggressive treatment plans including surgery, chemotherapy and radiotherapy are beneficial for patients who are likely to exhibit poor survival, not all osteosarcoma patients benefit from these treatments.

Imaging examinations play an important role in diagnosis and differential diagnosis, therapy response evaluation as well as prognosis prediction of osteosarcoma. However, radiological practice relies mainly on the subjective interpretation of imaging data by an expert radiologist and therefore is dependent on reader experience [5-8]. Quantitative, reader independent analysis, i. e. radiomics model or texture analysis, may supplement expert opinion and improve diagnostic, predictive and prognostic accuracy.

This systematic review will study the application of radiomics model or texture analysis in human patients with osteosarcoma.

Participants/population
Participants inclusion criteria:

1) patients with histologically confirmed osteosarcoma;
2) patients had undergone at least one pre- treatment pre- or post-treatment CT, MRI, PET/CT or PET/MR;
3) a radiomics model or texture analysis for stratification of tumor, prediction of response to therapy or prognosis of patients was established.

Participants exclusion criteria:
1) not human patients, e. g. cell line, xenotransplant;
2) not osteosarcoma, e. g. Ewing sarcoma;
3) no performed imaging procedure;
4) no radiomics model established or texture analysis performed.

Intervention(s), exposure(s)
Patients with osteosarcoma underwent at least one pre- treatment pre- or post-treatment CT, MRI, PET/CT or PET/MR with a radiomics model or texture analysis performed based on these imaging data.

Comparator(s)/control
Standard-of-care imaging.

Context
Studies describing radiomics model or texture analysis of CT, MRI, PET/CT or PET/MR in patients with osteosarcoma for stratification of tumor, prediction of response to therapy or prognosis will be included in this review. Studies must be with full-text available and sufficient information for assessing the methodological quality.

Main outcome(s)
The Radiomics Quality Score per study included in the review as a metric of the methodological quality of the studies. The systematic review aims to establish the quality level found in texture analysis research in osteosarcoma. This is pertinent to define which models may be further studied to achieve external validation and aim for clinical translation of research models. Furthermore, this systematic review shall identify methodological challenges which future studies should solve.

Measures of effect
The Radiomics Quality Score rating results will be used as the measures of effect for our main outcome. The Radiomics Quality Score rated resulting with a minimum score with -8 to 0 defined as 0% and a maximum score with 36 points defined as 100%.

Additional outcome(s)
The potential role of radiomics related to stratification of tumor, prediction of response to therapy or prognosis. If a sufficient number of studies attempts to answer a similar question, e.g. the correlation of radiomics model or image texture with response to the chemotherapy, a meta-analysis may be performed. Secondary outcomes of this review will be an assessment of the risk of bias in individual studies. The risk of bias will be assessed with the QUADAS tool, version 2.

Measures of effect
These measures are made during the data analysis phase.

Data extraction (selection and coding)
Study inclusion criteria:
1) studies are reported in English, Japanese, Chinese, German or French with institutional full-text availability;
2) the cohort consists of patients with histologically confirmed osteosarcoma;
3) patients had undergone at least one pre- treatment pre- or post-treatment CT, MRI, PET/CT or PET/MR;
4) a radiomics model or texture analysis for stratification of tumor, prediction of response to therapy or prognosis of patients was established.

Study exclusion criteria:
1) duplicate studies;

2) reviews, technical reports, letters to editors, comments to published studies, conference proceedings, case reports, brief communications and articles with insufficient information for assessing the methodological quality;
3) studies are reported other than English, Japanese, Chinese, German or French;
4) not human, not osteosarcoma, not radiomics or texture analysis studies.

A data collection tool will be established based on similar reviews and then trialed on two randomly chosen studies, which fulfilled all the inclusion criteria. These shall be used to train reviewers to appropriately apply the data extraction tool. In particular, two members of the research team will independently calculate the Radiomics Quality Score and assess the risk of bias using the QUADAS tool, version 2. The entire research team will assess the performance of the data extraction tool and justify modifications.

Risk of bias (quality) assessment
Quality assessment will be conducted using the dedicated Radiomics Quality Score. Furthermore, the risk of bias will be assessed using the QUADAS tool, version 2.

Strategy for data synthesis
A narrative synthesis will be provided with information presented in the text and/or tables to summarize and explain the characteristics and findings of the included studies. A quantitative synthesis will be done if the included studies are sufficiently homogenous. All analysis will be based on aggregate data.

Analysis of subgroups or subsets
If a sufficiently homogeneous subset of studies analyzed a single outcome parameter, e.g. prediction of response to the chemotherapy based on radiomics or texture analysis, a meta-analysis of this subgroup may be attempted.

Type and method of review
Diagnostic, Narrative synthesis, Prognostic, Systematic review

Funding sources/sponsors
This research is supported by National Natural Science Funds of China (No. 81771790) and Medicine and Engineering Combination Project of Shanghai Jiao Tong University (No. YG2019ZDB09).

Conflicts of interest
The authors declare that they have no competing interests.

Update of the systematic review
This systematic review is an update of published review (Eur Radiol. 2021;31(3):1526-1535). We decided to update according to the three-step decision framework (BMJ. 2016;354:i3507). Following topics have been discussed by the review group.

(1.1) Does the published review still address a current question? The preliminary search showed that the related articles doubled since the publication of this review. Therefore, we considered that the published review cannot address the current status of the topic. We also include two Chinese databases to identify relevant articles as possible.
(1.2) Has the review had good access or use? Reviews that are widely cited and used could be important to update should the need arise. The published review has been cited for 20 times since it published online on 02 Sep 2020. Therefore, we considered that this review is addressing a question that is valued, and therefore is worth updating.
(1.3) Did the review use valid methods and was it well conducted? The question is current and clearly defined. The published review assesses the quality of the literature published on radiomics or texture analysis in medical imaging of osteosarcoma in humans and identifies challenges impeding the clinical translation of proposed models for stratification of tumor, prediction of response to therapy or prognosis.
(2.1) Are there any new relevant methods? We noticed that there is a new suitable tool published for this review, i. e., Checklist for artificial intelligence in medical imaging (CLAIM) (Radiol Artif Intell 2(2):e200029). This checklist is proposed to aid authors and reviewers of AI manuscripts in medical imaging. The CLAIM modeled after the Standards for Reporting of Diagnostic Accuracy Studies (STARD) guideline and has

been extended to address applications of AI in medical imaging that include classification, image reconstruction, text analysis, and workflow optimization. The elements described in CLAIM are viewed as a “best practice” to guide authors in presenting their research. Although the feasibility of CLAIM has not been specifically tested in radiomics studies. We believe that radiomics studies as a subset of AI approach are suitable to be evaluated by CLAIM. Actually, one of the aims of our new systematic review is to find out whether CLAIM can better identify disadvantages in current radiomics studies.
(2.2) Are there any new studies or other information? We have slightly updated the search strategies used in the published review and running the searches as a preliminary search for the new review. Our preliminary search showed that the related articles doubled since the publication of published review. The updated search string is described in detail in the following search strategy section.
(3.1) Will the adoption of new methods change the findings or credibility? We planned to apply CLAIM (Radiol Artif Intell 2(2):e200029) as well as Transparent Reporting of a multivariable prediction model for Individual Prognosis Or Diagnosis (TRIPOD) checklist (Ann Intern Med. 2015;162(1):55-63) for study quality evaluation, in addition to the Radiomics Quality Score (RQS, Nat Rev Clin Oncol. 2017;14(12):749-762) and modified Quality Assessment of Diagnostic Accuracy Studies (QUADAS-2) tool (Ann Intern Med. 2011;155(8):529-536). We also decided to use the evidence rating system to evaluate the current evidence supporting radiomics application in osteosarcoma (Eur Radiol. 2021;31(7):4528-4537).
(3.2) Will the new studies, information, or data change the findings or credibility? We believed that there are valuable articles published. Overall, we decided to update this systematic review and meta-analysis, to present the current status of radiomics in osteosarcoma as well as improvements accomplished these two years.

We briefly summarized the changes we made for this updating systematic review.
(1) Data source: two Chinese databases, namely China National Knowledge Infrastructure (http://www.cnki.net) and Wanfang Data (https://www.wanfangdata.com.cn) will be included.
(2) Search string: the search string for three English databases will be updated, to allow studies based on SPECT and ultrasound images.
(3) Quality assessment: two new tools will be employed for study quality assessment, namely TRIPOD checklist (Ann Intern Med. 2015;162(1):55-63), and CLAIM (Radiol Artif Intell 2(2):e200029).
(4) Evidence level rating: we will employ an evidence level rating system (Eur Radiol. 2021;31(7):4528-4537) for rating the strength level of evidence supporting the radiomics application in osteosarcoma, based on results of meta-analyzes.
(5) Secondary study aim: we decided to find out whether CLAIM can better identify disadvantages in radiomics studies than currently recommended RQS and TRIPOD, as there are more and more radiomics studies applying deep learning method.
(6) Meta-analysis: we used stricter criteria for inclusion of studies into meta-analysis. We only included studies which calculated diagnostic performance on a validation dataset.

Supplementary Note S2 Search strategy and study selection

1. Study search strategy

1.1 PubMed search strategy

Available via https://pubmed.ncbi.nlm.nih.gov
Preliminary search date: 23 Mar 2020
Articles retrieved: 29
Formal search date: 30 Apr 2020
Articles retrieved: 30
Original search string:
('osteosarcoma'[Mesh] OR osseous sarcoma OR osteogenic sarcoma) AND ('magnetic resonance imaging'[Mesh] OR magnetic resonance imaging OR magnetic resonance OR MRI OR MR OR 'tomography, x-ray computed'[Mesh] OR computed tomography OR CT OR 'positron-emission tomography'[Mesh] OR positron emission tomography OR PET) AND (textural* OR texture* OR radiomics* OR radiomic* OR histogram*)

Updated preliminary search date: 15 Apr 2022
Articles retrieved: 55
Updated formal search date: 15 May 2022
Articles retrieved: 66
Updated search string:
('osteosarcoma'[Mesh] OR osseous sarcoma OR osteogenic sarcoma) AND ('magnetic resonance imaging'[Mesh] OR magnetic resonance imaging OR magnetic resonance OR MRI OR MR OR 'tomography, x-ray computed'[Mesh] OR computed tomography OR CT OR 'positron-emission tomography'[Mesh] OR positron emission tomography OR PET OR 'Tomography, Emission-Computed, Single-Photon'[Mesh] OR single photon emission computed tomography OR SPECT OR 'Ultrasonography'[Mesh] OR Ultrasound) AND (textural* OR texture* OR radiomics* OR radiomic* OR histogram*)

1.2 Embase search strategy

Available via www.embase.com
Preliminary search date: 23 Mar 2020
Articles retrieved: 35 (without filter)/21 (publication type article)
Formal search date: 30 Apr 2020
Articles retrieved: 35 (without filter)/21 (publication type article)
Original search string:
((‘osteosarcoma'/exp OR ‘osteosarcoma':ti,ab,kw) OR (‘osseous sarcoma’:ti,ab,kw) OR (‘osteogenic sarcoma'/exp OR 'osteogenic sarcoma’:ti,ab,kw)) AND ((‘radiomic’:ti,ab,kw) OR ('radiomics'/exp OR ‘radiomics':ti,ab,kw) OR (‘textural':ti,ab,kw) OR ('texture'/exp OR ‘texture':ti,ab,kw) OR (‘histogram'/exp OR ‘histogram’:ti,ab,kw)) AND ((‘magnetic resonance imaging'/exp OR 'magnetic resonance imaging':ti,ab,kw OR 'magnetic resonance’:ti,ab,kw) OR (MR:ti,ab,kw) OR (MRI:ti,ab,kw) OR ('computed tomography'/exp OR 'computed tomography':ti,ab,kw) OR (CT:ti,ab,kw) OR ('positron emission tomography'/exp OR 'positron emission tomography':ti,ab,kw) OR (PET:ti,ab,kw))

Updated preliminary search date: 15 Apr 2022
Articles retrieved: 68
Updated formal search date: 15 May 2022
Articles retrieved: 73
Updated search string:
(('osteosarcoma'/exp OR 'osteosarcoma':ti,ab,kw) OR ('osseous sarcoma':ti,ab,kw) OR ('osteogenic sarcoma'/exp OR 'osteogenic sarcoma':ti,ab,kw)) AND (('magnetic resonance imaging'/exp OR 'magnetic resonance imaging':ti,ab,kw OR 'magnetic resonance':ti,ab,kw) OR (MR:ti,ab,kw) OR (MRI:ti,ab,kw) OR ('computed tomography'/exp OR 'computed tomography':ti,ab,kw) OR (CT:ti,ab,kw) OR ('positron emission tomography'/exp OR 'positron emission tomography':ti,ab,kw) OR (PET:ti,ab,kw) OR ('single photon emission computed tomography'/exp OR 'single photon emission computed tomography':ti,ab,kw) OR (SPECT:ti,ab,kw) OR ('echography'/exp OR ultrasound:ti,ab,kw)) AND ((‘radiomic':ti,ab,kw) OR

('radiomics'/exp OR 'radiomics':ti,ab,kw) OR ('textural':ti,ab,kw) OR ('texture'/exp OR ‘texture':ti,ab,kw) OR ('histogram'/exp OR 'histogram':ti,ab,kw))

1.3 Web of Science (WOS) search strategy
Available via apps.webofknowledge.com
Preliminary search date: 23 Mar 2020
Articles retrieved: 33 (without filter)/ 24 (publication type article)
Formal search date: 30 Apr 2020
Articles retrieved: 33 (without filter)/ 24 (publication type article)
Original search string:
(TS=(osteosarcoma*) OR TS=(osseous sarcoma) OR TS=(osteogenic sarcoma)) AND (TS=(radiomic*) OR TS=(radiomics*) OR TS=(textural*) OR TS=(texture*) OR TS=(histogram*)) AND (TS=(magnetic resonance imaging) OR TS=(magnetic resonance) OR TS=(MRI) OR TS=(MR) OR TS=(computed tomography) OR TS=(CT) OR TS=(positron emission tomography) OR TS=(PET))

Updated preliminary search date: 15 Apr 2022
Articles retrieved: 60
Updated formal search date: 15 May 2022
Articles retrieved: 61
Updated search string:
(TS=(osteosarcoma*) OR TS=(osseous sarcoma) OR TS=(osteogenic sarcoma)) AND (TS=(radiomic*) OR TS=(radiomics*) OR TS=(textural*) OR TS=(texture*) OR TS=(histogram*)) AND (TS=(magnetic resonance imaging) OR TS=(magnetic resonance) OR TS=(MRI) OR TS=(MR) OR TS=(computed tomography) OR TS=(CT) OR TS=(positron emission tomography) OR TS=(PET) OR TS=(single photon emission computed tomography) OR TS=(SPECT) OR TS=(ultrasound))

1.4 China National Knowledge Infrastructure (CNKI) search strategy
Available via http://www.cnki.net
Preliminary search date: 01 Feb 2022
Articles retrieved: 14
Formal search date: 15 May 2022
Articles retrieved: 19
Search string: ("骨肉瘤" + "成骨肉瘤") * ("影像组学" + "直方图" + "纹理")
English translation: (“osteosarcoma” + “osseous sarcoma”) * (“radiomics” + “histogram” + “texture”)

1.5 Wanfang Data search strategy
Available via https://www.wanfangdata.com.cn
Preliminary search date: 01 Feb 2022
Articles retrieved: 28
Formal search date: 15 May 2022
Articles retrieved: 32
Search string: ("骨肉瘤" OR "成骨肉瘤") AND ("影像组学" OR "直方图" OR "纹理")
English translation: (“osteosarcoma” OR “osseous sarcoma”) AND (“radiomics” OR “histogram” OR “texture”)

This study search strategy has been tested in a pilot search to confirm its feasibility on 23 Mar 2020. The initial formal study search was performed on 06 Apr 2020. The initial formal search was performed on 30 Apr 2020. We further included China National Knowledge Infrastructure and Wanfang Data. We have updated the search string. The feasibility of study search strategy in two Chinese databases (China National Knowledge Infrastructure, and Wanfang Data) and three English databases (PubMed, Embase, Web of Science) were tested on 01 Feb 2022 and 15 Apr 2022, respectively. The update search was performed on 15 May 2022.

2. Study Selection
2.1 Studies included in systematic review
Study inclusion criteria:

1) studies are reported in English, Chinese, Japanese, German or French with institutional full-text availability;
2) the cohort consists of patients with histologically confirmed osteosarcoma;
3) patients had undergone at least one pre- or post-treatment CT, MRI, PET, SPECT, or ultrasound;
4) a quantitative image analysis or model for stratification of tumor, prediction of response to therapy or prognosis of patients was established.

Study exclusion criteria:
1) duplicate studies;
2) reviews, technical reports, letters to editors, comments to published studies, conference proceedings, case reports, brief communications and articles with insufficient information for assessing the methodological quality;
3) studies are reported other than English, Chinese, Japanese, German or French;
4) not human, not chondrosarcoma, not quantitative image analysis studies.

Contact with the authors was sought if the full-text version was not accessible otherwise. The reference lists of included studies and relevant reviews identified through the search were screened for additional, potentially eligible articles. Two reviewers both with 4-year-experience in radiology and radiomics research screened and selected studies independently. One of these two reviewers can read articles in English, Chinese, Japanese, German and French. The disagreements were resolved by a third reviewer with 30-year-experience in musculoskeletal radiology.

2.2 Studies included in meta-analyzes
As predetermined in the review protocol, if a sufficient number of studies attempts to answer a similar question, a meta-analysis could be performed. In current study, response to NAC predicted by MRI, were repeatedly addressed. There were studies calculated the model performance on the validation dataset. Therefore, these studies were included in the meta-analysis.

The studies included in meta-analysis should meet following criteria:
1) studies used a radiomic model to answer one of the above three clinical questions;
2) studies with documented sensitivity (Se), specificity (Sp), accuracy, positive predictive value (PPV), negative predictive value (NPV) and likelihood ratio (LR), diagnostic odds ratio (DOR), or with those could be calculated using published data.
3) data were calculated on a validation dataset.

The studies were excluded from meta-analysis because:
1) overlapping cohorts;
2) Two-by-two tables not documented, and could be calculated using published data.
3) data were not calculated on a validation dataset.

If multiple radiomic models were reported in a study, only the one with the best discrimination performance was included. If multiple radiomic models were aimed to answer different clinical questions, all of them were included. The two-by-two tables were directly extracted, if documented, or reconstructed based on available data, by one reviewer and then checked by another. The disagreements were resolved by a third reviewer. One of the reviewers has significant statistical expertise, and performed the meta-analysis.

Supplementary Note S3 Consensus reached during data extraction and quality assessment

Two reviewers who both with 4-year-experience in radiology and radiomics research discussed with multiple reviewers to make a consensus on additional topics of RQS, TRIPOD checklist, CLAIM, and QUADAS-2 tool of current review. Our review group consists with one radiologist with 30-year-experience in musculoskeletal radiology, one radiologist with 10-year-experience in image texture analysis and radiomics research and 28-year-experience in radiology, one orthopedist with 25-year-experience in bone and soft tissue tumor treatment, one pathologist with 35-year-experience in musculoskeletal pathology, and several other reviewers with different level of experience in radiology, orthopedics and pathology.

1. RQS

The RQS consists of 16 items concerning crucial aspects of radiomics studies, to assess their methodological quality. The reviewers performed RQS evaluation according to six key domains as previous reported. The following topics were discussed to reach a consensus:

(1) Multiple segmentation (domain 1): when there were two or more readers, the article earned an additional point if segmentation variability was considered. Automatic segmentation using a convolutional neural network or other automatic software earned a point as the method pursued better segmentation reproducibility.
(2) Validation (domain 2): if cross-validation or nested cross-validation was performed only within the training set, it was considered missing validation and scored -5 points, as previously described. If validation was performed on a dataset from the same institution, it scored +2 points. If the validation was based on a dataset from another institute, it scored +3 points.
(3) Non-radiomics feature (domain 3): non-radiomics features includes clinical features, laboratory test results, and radiologist's interruption of images. For studies applied deep learning method, the deep learning feature are considered as non-radiomics feature, since they were extracted through a method different from radiomics pipeline.
(4) Comparison with the gold standard (domain 3): in studies with differential diagnostic purpose and perdition of response to NAC, the judgment of radiologists before the post-operation histological assessment was considered the gold standard. Therefore, studies comparing the diagnostic or predictive performance of radiomics with that of radiologists scored 1 point. As there are several scoring systems for the prediction of prognosis in patients with osteosarcoma, such as TMN system or Ennkecking staging, if the studies compered radiomics models with these scoring systems, the studies scored 1 point on this topic. This item is different from the ground truth. For studies with differential diagnostic purpose and perdition of response to NAC, histological assessment is considered as the preferred criteria of ground truth. For prognosis in patients with osteosarcoma, follow-up is considered as the preferred criteria of ground truth.
(5) Biologic correlation (domain 3): studies that attempted to elucidate the possible correlations between radiomic features and microenvironments of tumors (e. g., variance measures the deviation of gray levels from the mean and represents the extent of the histogram, which may reflect on morphologic imaging performance) scored 1 point. We did not employ the criteria that correlations between radiomic features and genetic mutation status, since genetic mutation detection has not been widely accepted in the clinical settings.
(6) Clinical utility (domain 3): clinical utility is thought to be achieved when a biomarker leads to net improvement of health outcomes or provides information useful for prevention, diagnosis, treatment, and management of a disease. A study earned 2 points if the clinical utility was objectively 'measured', such as decision curve analysis to demonstrate net improvement. On the other hand, discussion of the potential utility of radiomics without proper analysis did not earn additional points.
(7) Open science and data (domain 6): the study gains 1 point for open science, if the code is available, or software and version is available, or images are available, or ROI is available.

2. TRIPOD

The TRIPOD checklist, consisting of 37 items in 22 criteria, was applied to determine the reporting completeness of the included prediction models. Since the TRIPOD checklist was originally produced for the clinical prediction model, it was partially modified for application in radiomics studies. The following topics were discussed to reach a consensus:

(1) Title (item 1): considered as complete if all elements of the type of study (development/validation/incremental value/or combination), the target population, and outcome are included.
(2) Study objective (items 2 and 3b): considered as complete if 'development' and/or 'validation' is explicitly written. Synonyms instead of development such as 'establish', 'build', 'investigate', and 'evaluate' were not considered as complete.
(3) Source of data (item 4a and 4b): whether the study was conducted in a randomized controlled trial, cohort, or registry with a consecutive, random, or convenience series. A study was considered as complete for item 4a when the terms 'retrospective' or 'prospective', inclusion period, and inclusion center were mentioned. A study was considered as complete for item 4b when the name of open-source data was provided, or declaim that the study was performed based on institutional dataset with a specific inclusion period.
(4) Clearly define all predictors used, including how and when they were measured (item 7a): the radiomics studies involve quantitative feature extraction through an automated process; thus, the element 'when' was ignored. Report any actions to blind assessment of predictors for the outcome and other predictors (item 7b): if radiomics studies were based on regions-of-interest and the blindness of readers to the reference standard was considered, they were recorded as complete. If 'blind' or 'unaware of' the reference standard was not explicitly written, it was considered as incomplete. Automatic segmentation was considered as complete.
(5) Specify type of the model, all procedures, and methods for internal validation (item 10b): considered as complete if all three elements, model type (e.g., logistic regression, Cox proportional hazards model), feature selection procedure to control overfitting, and methods of internal validation (cross-validation, bootstrap sample), were included. A regularization or penalization method such as the least absolute shrinkage and selection operator (LASSO) was considered as both a feature selection procedure and internal validation, as it contains 10-fold cross-validation as a default setting.
(6) Specify measure of model performance (item 10d): the article was considered as complete if both the discrimination and calibration index were written.
(7) Flow of participants (item 13a): considered as complete for 13a if a diagram or text description with the numbers of screened patients, excluded patients and included patients was provided.
(8) Describe how the predictions were calculated (item 10c), present the full prediction models (item 15a), and explain how to use the prediction model (item 15b): these items determine if an article describes how the obtained model predicted the outcome probabilities for an individual. If the articles described this in the methods (item 10c) and contained a full prediction model including all regression coefficients and the intercept or baseline hazard for a particular time point, they were considered as complete for item 15a. If the study contained explicit formula or a nomogram, the study was considered as complete for item 15b.
(9) Model update (items 10e and 17): If an article describes methods to adjust (recalibrate) or update a previously developed prediction model, the article is scored. This is different from 'comparison with gold standard' in RQS criterion 13, in that it requires recalibration of regression coefficients and hazard ratios in the pre-existing model, and was scored if it was completely reported.
(10) Difference between development and validation cohort (items 12 and 13c): considered as complete if a table comparing developing and testing dataset or text description was provided. The comparison needed to be present with *P* values.

3. CLAIM

The CLAIM is developed after the Standards for Reporting of Diagnostic Accuracy Study (STARD) guideline, and has been demonstrated as a useful tool to improve design and reporting of deep learning researches. This checklist is designed for clear, transparent and reproducible scientific communication about the application of AI in medical imaging. The CLAIM includes forty-two items in seven topics that should be viewed as a best practice to guide presentation of AI research. The CLAIM has seldomly been employed for quality assessment of radiomics studies. However, we assumed that CLAIM is suitable for radiomics studies evaluation, as radiomics is a subset of AI application in medical imaging. The following topics were discussed to reach a consensus:

(1) Title (item 1): This item considered as complete if the title and/ or abstract indicates the usage of AI methodology, such as histogram, texture analysis, radiomics, machine learning (or specific machine learning method), or deep learning (or neuron network).

(2) Study objective (item 4a), study hypotheses (item 4b) and study aim (item 6): These items are different. We considered the item 4a as complete if there is a sentence that declare “The aim of this study is to…”. We considered the item 4b as complete if there is a sentence that declare “The hypothesis of this study is…”. The item 6 is considered as complete if the following keywords were indicated, such as model creation, exploratory study, feasibility study, or noninferiority trial.
(3) Data source (item 7): This item is classified into six subitems, including data source (item 7a), data collection institutions (item 7b), imaging equipment vendors (item 7c), image acquisition parameters (item 7d), institutional review board approval (item 7e), participant consent (item 7f).
(4) Selection of data subsets, if applicable (item 10): For radiomics studies, segmentation is a necessary step. The available values for item are not documented, image cropping documented, and reproducible image cropping method documented.
(5) Provided reproducible model description (item 22a): This item needs the article to provide a complete and detailed structure of the model, including inputs, outputs, and all intermediate layers, in sufficient detail that another investigator could exactly re-construct the network. We considered this item as complete if all three elements, model type (e.g., logistic regression, Cox proportional hazards model), feature selection procedure to control overfitting, and methods of internal validation (cross-validation, bootstrap sample), were included. A regularization or penalization method such as the least absolute shrinkage and selection operator (LASSO) was considered as both a feature selection procedure and internal validation, as it contains 10-fold cross-validation as a default setting.
(6) Initialization of model parameters (item 24): For the deep learning models, indicate how the parameters of the model were initialized. For radiomics only models, this item is considered as complete, if feature reduction and selection is described for initialization of the radiomics models using the training dataset. Later this model could be validated using the validation dataset, and tested using the testing dataset.
(7) Flow of participants or cases, using a diagram to indicate inclusion and exclusion (item 33): considered as complete for item 33 if a diagram or text description with the numbers of screened patients, excluded patients and included patients was provided.
(8) Benchmark of performance (item 35b): Report the final model’s performance on the test partition. Benchmark the performance of the AI model against current standards, such as histopathologic identification of disease or a panel of medical experts with an explicit method to resolve disagreements. This item refers to the comparison to ground truth, rather than the ground truth.

4. QUADAS-2

The QUADAS-2 tool was developed for the risk of bias and concern of application assessment. The tool was tailored to our study by two reviewers who both with 4-year-experience in radiology and radiomics research through modifying signaling questions specific to current study. The following topics were discussed to reach a consensus:

(1) Patient selection: we used the three original signal questions for our review: “was a consecutive or random sample of patients enrolled?”, “was a case–control design avoided?”, and “did the study avoid inappropriate exclusions?”, since these questions were suitable for radiomics studies. The studies which avoided case-control design, and clearly declare consecutive or random sample inclusion were rated as “low risk”. We considered the studies that have provided a clear inclusion period as consecutive. The studies with case-control design or inappropriate exclusions, were rated as “high risk”. The studies without a clear declaration of consecutive or random sample inclusion were rated as “unclear”.
(2) Index test: the original signal questions of this domain were “were the index test results interpreted without knowledge of the results of the reference standard?” and “if a threshold was used, was it prespecified?” However, the radiomics process is an automatic pipeline, and the knowledge of the results of the reference standard would has limited influence on the results interpretation, since the researchers who did the segmentation did not directly make the interpretation. For radiomics studies, it is not possible to prespecify a threshold for radiomics features. Therefore, these two questions were not considered. For replacement, we have added three additional questions specified for radiomics methodology: “were the imaging acquisition protocol, image processing approach described in detail?”, “were the segmentation method(s), and feature extraction software described in detail?”, and “was the validation independent (i. e. external)?”. We considered cross-validation and bootstrapping as internal validation. The external validation may be performed using three different strategies including temporal (i.e., data obtained in newly recruited patients), geographic (i.e., data collected in a different institution), or split-sample (i.e., data split from the

entire dataset and kept untouched for the test). We considered these three modified signal questions were tightly related to the risk of bias of radiomics workflow. The studies without detailed imaging acquisition protocol, image processing approach, the segmentation method(s), and feature extraction software, or validated with an internal dataset were rated as “high risk”. The studies with detailed methodological description and external validation were rated as “low risk”

(3) Reference standard: we used one of the two original signal questions for our review: “is the reference standard likely to correctly classify the target condition?”. We considered pathohistological assessment as the adequate standard for osteosarcoma in diagnostic and prediction of response to NAC researches. Both excision or biopsy were acceptable sample source for pathohistological assessment. For prognosis researches, an adequate follow-up with suitable examinations were needed. We did not used the other original signal question: “were the reference standard results interpreted without knowledge of the results of the index test?”, since the results interpretation by reference standard was always before the results interpretation by the radiomics model, and these two processes were performed separately. The studies with adequate standard were rated as “low risk” and those using suboptimal standard were rated as “high risk”.

(4) Follow and timing: there were four original questions: “was there an appropriate interval between index tests and reference standard?”, “did all patients receive a reference standard?”, “did all patients receive the same reference standard?”, and “were all patients included in the analysis?”. We only used the first signal question. Since the timing of radiomics workflow did not influence the results, we focus on the timing of imaging and reference standard, especially the interval between imaging and surgery or biopsy. We did not use the second and third signal question, because all the patients had had to be assessed with adequate standard before the radiomics workflow began. We did not use the last signal question, because not all the patients were included in the analysis due the nature of radiomics workflow, which always divided patients into training dataset and validation dataset. The modified signal question was “was there an appropriate interval between imaging and reference standard?”. If the reference standard was pathohistological assessment after surgery or biopsy, the studies with a clear declaration of adequate interval between imaging and surgery or biopsy were rated as “low risk”; those without a clear declaration were rated as “unclear”; those with a clear declaration of inadequate interval were rated as “high risk”. If the reference standard was clinical diagnosis, or follow-up for prognosis purpose, the studies were rated as “low risk”, since there was not suitable to set an appropriate interval.

Supplementary Note S4 Data synthesis and analysis methods

1. Statistical Analysis

The statistical analysis was performed with R language version 4.1.3 within RStudio version 1.4.1106. A two-tailed *p*-value < 0.05 was recognized as statistical significance, unless otherwise specified. 16 items of the RQS were scored. The RQS score and percentage of the ideal score were described as score and percentage of score to ideal score for each item, respectively. A total of 37 items and subitems on the TRIPOD checklist was scored. During the calculation of TRIPOD, the "if done" or "if relevant" items (5c, 11, and 14b) and validation items (10c, 10e, 12, 13, 17, and 19a) were excluded from both the denominator and numerator. A total of 53 items and subitems of CLAIM was scored. During the calculation of CLAIM, the "if applicable" item (27) was excluded from both the denominator and numerator. In the cases where a score of one point per item was obtained, the study was considered to have basic adherence to each item of the RQS rating, TRIPOD checklist and CLAIM. The adherence rate of RQS rating, TRIPOD checklist and CLAIM were calculated as proportion of the number of articles with basic adherence to number of total articles. The result of QUADAS-2 risk of bias and application concern assessment was summarized as proportions of high risk, low risk and unclear.

In our statistical analysis plan, the Pearson or Spearman correlation test was planned to use for the correlation analysis between ideal percentage of RQS, TRIPOD adherence rate and CLAIM adherence rate. Subgroup analysis was planned to perform to determine whether factor influenced on the study quality including journal type, first authorship, biomarker, and imaging modality. According to normality test results, independent *t*-test or Mann-Whitney's U-test were used for intergroup differences, and one way analysis of variance or Kruskal-Wallis H-test were applied for multiple comparisons. The Bonferroni method was used for post-hoc correction.

In the formal statistical analysis stage, the Kolmogorov-Smirnov test showed that ideal percentage of RQS, TRIPOD adherence rate and CLAIM adherence rate presented normal distribution (all P > 0.05). Therefore, Pearson correlation test was used for the correlation analysis between RQS, TRIPOD, CLAIM. The correlation was considered as high, if $|r|\geq0.8$; moderate, if $0.5\leq|r|<0.8$; low, if $0.3\leq|r|<0.5$; and not correlated if $|r|<0.3$. Subgroup analysis was performed to determine whether factor influenced on ideal percentage of RQS, TRIPOD adherence rate and CLAIM adherence rate, including journal type, first authorship, imaging modality, and publication period, using independent t test or one-way analysis of variance. A two-tailed P value < 0.05 indicated statistical significance, unless otherwise specified. Post-hoc multiple comparisons were performed using Tukey-Kramer method, the significance threshold is 0.05 for the adjusted P value using Bonferroni method.

2. Meta-analysis

The Stata software version 15.1 with metan, midas, and metandi packages was employed for meta-analysis. In current review, the prediction of response to NAC by MRI, was repeatedly addressed. There were four radiomics model tested within test dataset. These models were included in the meta-analysis. If multiple radiomic models were reported in a study, only the one with the highest area under the receiver operating curve (AUC), or the highest Youden' s index or the highest accuracy, if no AUC was reported, was included. If multiple radiomic models were aimed to answer different clinical questions, all of them were included. Due to the relatively insufficient sample size of included studies, we conducted the meta-analyzes with all available data.

One reviewer directly extracted or reconstructed the two-by-two tables based on available data; and then another reviewer cross-checked the results. The diagnostic odds ratio (DOR) and its corresponding 95% confidence interval (CI) were quantitatively synthesized as the main effect using random-effect model, and the corresponding *p*-value was calculated. Sensitivity, specificity, positive and negative likelihood ratio and their 95%CIs were also calculated, and relevant forest plots were obtained. Additionally, forest plots were drawn to show the heterogeneity in sensitivity, specificity, positive likelihood ratio, negative positive likelihood ratio and diagnostic odds ratio. A summary receiver operating characteristic (SROC) curve was plotted to visually show the diagnostic accuracy.

For assessment of heterogeneity between the included studies, the Cochran's Q and the I^2 statistic were calculated. Measuring inter-study dispersion assumes that, if all studies were methodologically identical

and variation in results were only due to the random selection of study participants, the effect sizes would follow a chi-squared distribution. Cochran's Q assesses the hypothesis that the distribution of results is homogenous and p-values < 0.05 would generally lead to the rejection of this null-hypothesis. As with a small number of studies Cochran's Q can be distorted, I^2, a measure for how much of the variability between effect size estimates is due to methodological heterogeneity rather than sampling error, was also reported. I^2 values of 25% and less are usually considered to be low or unimportant, 25% to 50% moderate and values above 75% are considered high.

A funnel plot and Deeks funnel plot were drawn to visually assessed publication bias. Egger's and Begg's tests were performed to assess the publication bias and a p-value > 0.1 indicated a low publication bias. Trim and fill method was used to estimate the number of missing studies. A Deeks funnel asymmetry test was also constructed to explore the risk of publication bias, and a p-value > 0.10 indicated a low publication bias. Excess significance evaluation and 10% credibility ceiling calculation were not necessary in current review. Since none of the clinical question included in meta-analysis met the criteria of >1000 samples.

The code used for Stata programming is present as follows.

```
// tp = true positive, fp = false positive, fn = false negative, tn = ture nagative
// For sensitivity, specificity, positive likelihood ratio, negative likelihood ratio and diagnostic odds ratio plot
midas tp fp fn tn, res(all)
midas tp fp fn tn, uforest(dss) id (studyid) ford fors
midas tp fp fn tn, uforest(dlr) id (studyid) ford fors
midas tp fp fn tn, texts(0.6) uforest(dlor) id (studyid) ford fors
// For HSROC curve plot
metandi tp fp fn tn, plot
midas tp fp fn tn, sroc(conf)
// For heterogeneity
midas tp fp fn tn, res(het)
// For funnel plot, and Egger's and Begg's test
gen d=sqrt(3) * (log(tp)+log(tn)-log(fp)-log(fn))/3.14
replace d=sqrt(3) * (log(tp+0.5)+log(tn+0.5)-log(fp+0.5)-log(fn+0.5))/3.14 if d==.
gen vard=3 * (1/tp+1/fp+1/fn+1/tn)/(3.14 * 3.14)
replace vard=3 * (1/(tp+0.5)+1/(fp+0.5)+1/(fn+0.5)+1/(tn+0.5))/(3.14 * 3.14) if vard==.
gen sed=sqrt(vard)
metafunnel d sed
metabias d sed, egger
metabias d sed, begg
// For Deeks funnel plot, and Deeks funnel plot asymmetry test
midas tp fp fn tn, pubbias
// For trim and fill method analysis
gen logor= log((tp * tn)/(fp * fn))
replace logor=log(((tp+0.5) * (tn+0.5))/((fp+0.5) * (fn+0.5))) if logor==.
gen selogor=sqrt(1/tp+1/fp+1/fn+1/tn)
replace selogor=sqrt(1/(tp+0.5)+1/(fp+0.5)+1/(fn+0.5)+1/(tn+0.5)) if selogor==.
metatrim logor selogor, eform funnel
```

3. Clinical value and Level of Evidence

The pieces of evidence supporting clinical values of radiomics models were categorized into five levels (convincing, highly suggestive, suggestive, weak, and not suggestive; Eur Radiol. 2021;31(7):4528-4537) based on results of meta-analysis. The process of evidence category requires multiple results based on

meta-analysis, including a p value of pooled analysis with random model, events calculation, the largest study reaches statistical significance, assessment of heterogeneity by I^2 assessment, the null value excluded by the 95% predictive interval, small-study effects by Egger's test, excess significance, and 10% credibility ceiling calculation.

Supplementary Table S1 Data extraction sheet

Field	Item
Bibliographical Information	The Title of The Study
	The First Authorship of The Study
	Published Year
	Published Journal
	Impact Factor of Published Journal
	Published Volume
	Published Issue
	Published Page
	Country
	Study ID, determined by First Author + Year, + Journal if needed
Study Characteristics	Study Design
	Patient Condition
	Patient Gender
	Patient Age
	Imaging Modality
	Predictor
	Outcome
	Reference Standard
	Data Splitting
Radiomics Considerations	ROI Segmentation
	Radiomics Feature Extraction Details
	Radiomics Feature Reduction Details
	Radiomics Feature Selection Details
	Selector
Model Metrics	Sample Size
	Number of Events (True Positive, False Positive, False Negative, True Negative)
	Sensitivity
	Specificity
	Accuracy
	Positive Predictive Value (PPV)
	Negative Predictive Value (NPV)
	Positive Likelihood Ratio (PLR)
	Negative Likelihood Ratio (NLR)
	Diagnostic Odds Ratio (DOR)

Note: none.

Supplementary Table S2 RQS elements according to six key domains

Domain	RQS#	RQS scoring item	Points and Interpretation
**Domain 1:** Protocol quality and stability in image and segmentation (0 to 5)	1	**Image protocol quality** - well-documented image protocols (for example, contrast, slice thickness, energy, etc.) and/or usage of public image protocols allow reproducibility/replicability	+ 1 if protocols are well-documented<br>+ 1 if public protocol is used
	2	**Multiple segmentations** - possible actions are: segmentation by different physicians/algorithms/software, perturbing segmentations by (random) noise, segmentation at different breathing cycles. Analyse feature robustness to segmentation variabilities	+ 1 if segmented multiple times (different physicians, algorithms, or perturbation of regions of interest)
	3	**Phantom study on all scanners** - detect inter-scanner differences and vendor-dependent features. Analyse feature robustness to these sources of variability	+ 1 if texture phantoms were used for feature robustness assessment
	4	**Imaging at multiple time points** - collect images of individuals at additional time points. Analyse feature robustness to temporal variabilities (for example, organ movement, organ expansion/ shrinkage)	+ 1 multiple time points for feature robustness assessment
**Domain 2:** Feature selection and validation (-8 to 8)	5	**Feature reduction or adjustment for multiple testing** - decreases the risk of overfitting. Overfitting is inevitable if the number of features exceeds the number of samples. Consider feature robustness when selecting features	- 3 if neither measure is implemented<br>+ 3 if either measure is implemented
	12	**Validation** - the validation is performed without retraining and without adaptation of the cut-off value, provides crucial information with regard to credible clinical performance	− 5 if validation is missing<br>+ 2 if validation is based on a dataset from the same institute/<br>+ 3 if validation is based on a dataset from another institute/<br>+ 4 if validation is based on two datasets from two distinct institutes/<br>+4 if the study validates a previously published signature/<br>+5 if validation is based on three or more datasets from distinct institutes<br>*Datasets should be of comparable size and should have at least 10 events per model feature
**Domain 3:** Biologic/clinical validation and utility (0 to 6)	6	**Multivariable analysis with non-radiomics features** (for example, EGFR mutation) - is expected to provide a more holistic model. Permits correlating/inferencing between radiomics and non-radiomics features	+ 1 if multivariable analysis with non-radiomics features

	7	**Detect and discuss biological correlates** - demonstration of phenotypic differences (possibly associated with underlying gene–protein expression patterns) deepens understanding of radiomics and biology	+ 1 if present
	13	**Comparison to gold standard** - assess the extent to which the model agrees with/is superior to the current 'gold standard' method (for example, TNM-staging for survival prediction). This comparison shows the added value of radiomics	+ 2 for comparison to gold standard
	14	**Potential clinical utility** - report on the current and potential application of the model in a clinical setting (for example, decision curve analysis)	+ 2 for reporting potential clinical utility
**Domain 4:** Model performance index (0 to 5)	8	**Cut-off analyses** - determine risk groups by either the median, a previously published cut-off or report a continuous risk variable. Reduces the risk of reporting overly optimistic results	+ 1 if cutoff either pre-defined or at median or continuous risk variable reported
	9	**Discrimination statistics** - report discrimination statistics (for example, C-statistic, ROC curve, AUC) and their statistical significance (for example, p-values, confidence intervals). One can also apply resampling method (for example, bootstrapping, cross-validation)	+ 1 if a discrimination statistic and its statistical significance are reported<br>+ 1 if a resampling method technique is also applied
	10	**Calibration statistics** - report calibration statistics (for example, Calibration-in-the-large/slope, calibration plots) and their statistical significance (for example, *P*-values, confidence intervals). One can also apply resampling method (for example, bootstrapping, cross-validation)	+ 1 if a calibration statistic and its statistical significance are reported<br>+ 1 if a resampling method technique is also applied
**Domain 5:** High level of evidence (0 to 8)	11	**Prospective study registered in a trial database** - provides the highest level of evidence supporting the clinical validity and usefulness of the radiomics biomarker	+ 7 for prospective validation of a radiomics signature in an appropriate trial
	15	**Cost-effectiveness analysis** - report on the cost-effectiveness of the clinical application (for example, QALYs generated)	+ 1 for cost-effectiveness analysis
**Domain 6:** Open science and data (0 to 4)	16	**Open science and data** - make code and data publicly available. Open science facilitates knowledge transfer and reproducibility of the study	+ 1 if scans are open source<br>+ 1 if region of interest segmentations are open source<br>+ 1 if code or software is open source<br>+ 1 if radiomics features are calculated on a set of representative ROIs and the calculated features and representative ROIs are open source
	Total points (36 = 100%)		

Note: RQS = Radiomics Quality Score.

Extracted from Lambin P, Leijenaar RTH, Deist TM, et al. Radiomics: the bridge between medical imaging and personalized medicine. Nat Rev Clin Oncol. 2017;14(12):749-762.

Insights Imaging (2022) Zhong J, Hu Y, Zhang G, et al.

Supplementary Table S3 TRIPOD reporting completeness checklist

Section	TRIPOD#	Item	Explanation	Values
**Title and Abstract**	1	**Title -** identify developing/validating a model, target population, and the outcome	#1: considered as complete if all elements of the type of study (development, validation, incremental value or combination), the target population, and outcome are included.	0. Not documented<br>1. Complete
	2	**Abstract** - provide a summary of objectives, study design, setting, participants, sample size, predictors, outcome, statistical analysis, results, and conclusions	#2 and #3b: considered as complete if ‘development’ and/or ‘validation’ is explicitly written. Synonyms instead of development such as ‘establish’, ‘build’, ‘investigate’, and ‘evaluate’ were not considered as complete.	0. Not complete<br>1. Complete
**Introduction**	3a	**Background -** Explain the medical context and rationale for developing/validating the model	#3a: considered as complete if at least a simple sentence was provided to introduce the medical context and rationale for developing/validating the model.	0. Not complete<br>1. Complete
	3b	**Objective -** Specify the objectives, including whether the study describes the development/validation of the model or both.	#2 and #3b: considered as complete if ‘development’ and/or ‘validation’ is explicitly written. Synonyms instead of development such as ‘establish’, ‘build’, ‘investigate’, and ‘evaluate’ were not considered as complete	0. Not complete<br>1. Complete
**Methods**	4a	**Source of data -** describe the study design or source of data (randomized trial, cohort, or registry data)	#4a: whether the study was conducted in a randomized controlled trial, cohort, or registry with a consecutive, random, or convenience series. A study was considered as complete when the terms ‘retrospective’ or ‘prospective’ were mentioned.	0. Not documented<br>R. Retrospective<br>P. Prospective<br>RP. Both retrospective and prospective
	4b	**Source of data -** specify the key dates	#4b: provide the name of open-source data, or declaim that the study was performed based on institutional dataset with a specific inclusion period.	0. Not documented<br>L. Local data collection<br>P. Public data<br>LP. Both local and public data
	5a	**Participants -** specify key elements of the study setting including number and location of centers	#5a: number and location of centers should be declared in multicenter studies; monocenter study should state the location of that the study performed.	0. Not documented<br>SC. Single-center data<br>MC. Multi-center data
	5b	**Participants** - describe eligibility criteria for participants (inclusion and exclusion criteria)	#5b: considered as complete if a structured criterion of inclusion and exclusion were provided; only disease name was not considered as complete.	0. Not documented<br>1. Documented

	5c	**Participants** - give details of treatment received, *if relevant*	#5c: treatments are relevant in prognostic studies as they modify outcomes and relevant information should be reported.	0. Not documented<br>1. Documented
	6a	**Outcome** - clearly define the outcome, including how and when assessed	#6a: the method of assessment, e.g., histology and experience of pathologists; follow-up, frequency and modality; or expert's opinion and experience of experts.	0. Not defined<br>1. Defined either explicitly or by reference to a Common Data Element
	6b	**Outcome** - report any actions to blind assessment of the outcome	#6b: describe weather the outcome is ideally assessed while blinded to information about the predictors.	0. Not documented<br>1. Documented
	7a	**Predictors** - clearly define all predictors, including how and when assessed	#7a: the radiomics studies involve quantitative feature extraction through an automated process; thus, the element 'when' was ignored.	0. Not documented<br>1. Documented
	7b	**Predictors** - report any actions to blind assessment of predictors for the outcome and other predictors	#7b: if radiomics studies were based on regions-of-interest and the blindness of readers to the reference standard was considered, they were recorded as complete. If 'blind' or 'unaware of' the reference standard was not explicitly written, it was considered as incomplete. Automatic segmentation was considered as complete.	0. Not documented<br>1. Documented
	8	**Sample size** - explain how the study size was arrived at	#8: considered as complete if the database, software or method, and results were described.	0. Not documented<br>1. Documented
	9	**Missing data** - describe how missing data were handled with details of any imputation method	#9: considered as complete if the imputation method was described when there is missing data, or how to excluded the insufficient data when imputation was not performed	0. Not documented<br>E. Missing data excluded from analysis<br>I. Missing data included in analysis
	10a	**Statistical analysis methods** - describe how predictors were handled	#10a: considered as complete if the statistical analysis method (e.g., t test, chi-square test) were included, and suitable for the variable type.	0. Not documented<br>1. Documented
	10b	**Statistical analysis methods** - specify type of model, all model-building procedures (any predictor selection), and method for internal validation	#10b: considered as complete if all three elements, model type (e.g., logistic regression, Cox proportional hazards model), feature selection procedure to control overfitting, and methods of internal validation (cross-validation, bootstrap sample), were included. A regularization or penalization method such as the least absolute shrinkage and selection operator (LASSO) was considered as both a feature selection procedure and internal	0. Not documented<br>1. Documented

			validation, as it contains 10-fold cross-validation as a default setting.	
	10d	**Statistical analysis methods -** specify all measures used to assess model performance and if relevant, to compare multiple models (discrimination or calibration)	#10d: the article was considered as complete if both the discrimination and calibration index were written	0. Not documented<br>1. Documented
	11	**Risk groups -** provide details on how risk groups were created, if done	#11: considered as complete if the cutoffs were provided, e.g., disease stage, predictive absolute incidence, or risk rate.	0. Not documented<br>1. Documented
**Results**	13a	**Participants -** describe the flow of participants, including the number of participants with and without the outcome. A diagram may be helpful.	#13a: considered as complete if a diagram or text description with the numbers of screened patients, excluded patients and included patients was provided.	0. Not documented<br>1. Documented
	13b	**Participants -** describe the characteristics of the participants, including the number of participants with missing data for predictors and outcome	#13b: considered as complete if a table or text description was provided.	0. Not documented<br>1. Documented
	14a	**Model development -** specify the number of participants and outcome events in each analysis	#14a: considered as complete if a table or text description was provided.	0. Not documented<br>1. Documented
	14b	**Model development -** report the unadjusted association between each candidate predictor and outcome, *if done*	#14b: considered as complete if the metrics and their confidence interval were provided.	0. Not documented<br>1. Documented
	15a	**Model specification -** present the full prediction model to allow predictions for individuals (regression coefficients, intercept)	#10c, #15a, and #15b: these items determine if an article describes how the obtained model predicted the outcome probabilities for an individual. If the articles described this in the methods (item 10c) and contained a full prediction model including all regression coefficients and the intercept or baseline hazard for a particular time point, they were considered as complete for item 15a. If the study contained explicit formula or a nomogram, the study was considered as complete for item 15b.	0. Not documented<br>1. Documented
	15b	**Model specification -** explain how to the use the prediction model (nomogram, calculator, etc)	#10c, #15a, and #15b: these items determine if an article describes how the obtained model predicted the outcome probabilities for an individual. If the articles described this in the methods (item 10c) and contained a full prediction model	0. Not documented<br>1. Documented

			including all regression coefficients and the intercept or baseline hazard for a particular time point, they were considered as complete for item 15a. If the study contained explicit formula or a nomogram, the study was considered as complete for item 15b.	
	16	**Model performance -** report performance measures (with confidence intervals) for the prediction model	#16: considered as complete if the metrics (at least the discrimination outcome) and their confidence interval were provided.	0. Diagnostic performance reported without measure of precision<br>1. Diagnostic performance reported with confidence interval or standard error
**Discussion**	18	**Limitations -** Discuss any limitations of the study	#18: considered as complete if there was a limitation paragraph, usually the paragraph before the conclusion.	0. Not discussed<br>1. Discussed
	19b	**Interpretation -** Give an overall interpretation of the results	#19b: considered as complete if there was an interpretation of results paragraph, usually the paragraph of discussion.	0. Not documented<br>1. Documented
	20	**Implications -** Discuss the potential clinical use of the model and implications for future research	#20: considered as complete if there was text description or decision curve analysis. This is different from 'clinical validity' in RQS criterion 14, that the decision curve analysis was necessary.	0. Not discussed<br>1. Discussed
**Validation (types 2a, 2b, 3, and 4)**	10c	**Statistical analysis methods -** describe how the predictions were calculated	#10c, #15a, and #15b: these items determine if an article describes how the obtained model predicted the outcome probabilities for an individual. If the articles described this in the methods (item 10c) and contained a full prediction model including all regression coefficients and the intercept or baseline hazard for a particular time point, they were considered as complete for item 15a. If the study contained explicit formula or a nomogram, the study was considered as complete for item 15b.	0. Not documented<br>1. Documented
	10e	**Statistical analysis methods -** describe any model updating (recalibration), *if done*	#10e and #17: If an article describes methods to adjust (recalibrate) or update a previously developed prediction model, the article is scored. This is different from 'comparison with gold standard' in RQS criterion 13, in that it requires recalibration of regression coefficients and hazard ratios in the pre-existing model, and was scored if it was completely reported.	0. Not documented<br>1. Documented<br>n/a. Not updating

	12	**Development vs. validation** - Identify any differences from the development data in setting, eligibility criteria, outcome, and predictors	#12 and #13c: considered as complete if a table comparing developing and testing dataset or text description was provided.	0. Not documented<br>1. Documented
	13c	**Participants (for validation)** - show a comparison with the development data of the distribution of important variables	#12 and #13c: considered as complete if a table comparing developing and testing dataset or text description was provided.	0. Not documented<br>1. Documented
	17	**Model updating** - report the results from any model updating, *if done*	#10e and #17: If an article describes methods to adjust (recalibrate) or update a previously developed prediction model, the article is scored. This is different from 'comparison with gold standard' in RQS item 13, in that it requires recalibration of regression coefficients and hazard ratios in the pre-existing model, and was scored if it was completely reported.	0. Not documented<br>1. Documented<br>n/a. Not updating
	19a	**Interpretation (for validation)** - discuss the results with reference to performance in the development data and any other validation data	#19a: considered as complete if there was a paragraph that discuss the influence of difference between development and validation data on the model performance. The performance of the model in the validation study should be discussed and placed in context to the model performance in the original development study and with any other existing validation studies of that model. One should highlight the main results, as well as any biases that may have affected the comparison. When the validation study shows a different (usually poorer) performance, reasons should be discussed to enhance interpretation.	0. Not documented<br>1. Documented
**Other Information**	21	**Supplementary information** - provide information about the availability of supplementary resources, such as study protocol, Web calculator, and data sets	#21: considered as complete if the study provided supplementary materials and/or links for online resources, or declared that all data were provided in the manuscript.	0. Not documented<br>1. Documented
	22	**Funding** - give the source of funding and the role of the funders for the present study	#22: considered as complete if the source of funding and the role of the funders were both declared.	0. Not documented<br>F. Funding source documented<br>FR. Funding source and role documented<br>NF. Stated no funding received

Note: TRIPOD = Transparent Reporting of a multivariable prediction model for Individual Prognosis Or Diagnosis.
Extracted from Collins GS, Reitsma JB, Altman DG, Moons KG. Transparent reporting of a multivariable prediction model for individual prognosis or diagnosis (TRIPOD): the TRIPOD statement. Ann Intern Med. 2015;162(1):55-63.

Supplementary Table S4 CLAIM for authors and reviewers

Section / Topic	CLAIM#	Item	Explanation	Values
**TITLE / ABSTRACT**				
**Title or abstract**	1	Identification as a study of AI methodology	#1: considered as complete if the title and/ or abstract indicates the usage of AI methodology, such as histogram, texture analysis, radiomics, machine learning (or specific machine learning method), or deep learning (or neuron network).	0. Not specified<br>1. Specified
**Abstract**	2	Structured summary of study design, methods, results, and conclusions.	#2: considered as complete if all following items is provided: study design, methods, results, and conclusions. However, the original version recommended to present: (1) Provide an overview of the study population (number of patients or examinations, number of images, age and sex distribution). (2) Indicate if the study is prospective or retrospective, and summarize the statistical analysis that was performed. (3) When presenting the results, be sure to include *P* values for any comparisons. (4) Indicate whether the software, data, and/or resulting model are available publicly.	0. Not included<br>1. Included
**INTRODUCTION**				
**Background**	3	Scientific and clinical background, including the intended use and clinical role of the AI approach	#3: considered as complete if at least a simple sentence was provided to introduce the medical context and rationale for developing/validating the model. Address an important clinical, scientific, or operational issue. Describe the study's rationale, goals, and anticipated impact. Summarize related literature and highlight how the investigation builds upon and differs from that work. Guide readers to understand the context for the study, the underlying science, the assumptions underlying the methodology, and the nuances of the study.	0. Not provided<br>1. Provided
**Study objectives and hypotheses**	4a	Study objectives	#4a and #4b: considered as complete if there are two sentences describe the aim/ purpose/ objective of the study (4a), and/ or the hypothesis of the study (4b), respectively. Define clearly the clinical or scientific question to be answered; avoid vague statements or descriptions of a process. Limit the chance of post hoc data dredging by specifying the study's hypothesis a priori. Identify a compelling problem to address. The study's objectives and hypothesis will guide sample size	0. Not provided<br>1. Provided
	4b	Study hypotheses		0. Not documented<br>1. Documented

			calculations and whether the hypothesis will be supported or not.	
**METHODS**				
**Study Design**	5	Prospective or retrospective study	#5: considered as complete if the study indicates whether the study is retrospective or prospective. It is recommended to evaluate predictive models in a prospective setting if possible, but if not, this item is not considered as incomplete.	0. Not documented<br>R. Retrospective<br>P. Prospective
	6	Study goal, such as model creation, exploratory study, feasibility study, non-inferiority trial	#6: considered as complete if the following keywords were indicated. (1) Define the study's goal, such as model creation, exploratory study, feasibility study, or noninferiority trial. For classification systems, state the intended use, such as diagnosis, screening, staging, monitoring, surveillance, prediction, or prognosis. (2) Indicate the proposed role of the AI algorithm relative to other approaches, such as triage, replacement, or add-on. (3) Describe the type of predictive modeling to be performed, the target of predictions, and how it will solve the clinical or scientific question.	0. Not documented<br>1. Documented
**Data**	7a/94%	Data source	#7: each subitems are evaluated respectively. #7a: not documented, local data source, public data source, local and public data source. #7b: not documented, single-center data, multi-center data. #7c: not documented, single vendor, multiple vendors. State the source of data and indicate how well the data match the intended use of the model. Describe the targeted application of the predictive model to allow readers to interpret the implications of reported accuracy estimates. Reference any previous studies that used the same dataset and specify how the current study differs. Adhere to ethical guidelines to assure that the study is conducted appropriately; describe the ethics review and informed consent. Provide links to data sources and/or images, if available. Authors are strongly encouraged to deposit data and/or software used for modeling or data analysis in a publicly accessible repository.	0. Not documented<br>L. Local data collection<br>P. Public data<br>LP. Both local and public data
	7b/72%	Data collection institutions		0. Not documented<br>SC. Single-center data<br>MC. Multi-center data
	7c/51%	Imaging equipment vendors		0. Not documented<br>SV. Single vendor<br>MV. Multiple vendors
	7d/37%	Image acquisition parameters		0. Not documented<br>1. Documented
	7e/48%	Institutional review board approval		0. Not documented<br>1. Documented
	7f/17%	Participant consent		0. Not documented<br>1. Documented
	8/27%	Eligibility criteria: how, where, and when potentially eligible participants or studies were	#8: considered as complete if a structured criterion of inclusion and exclusion were provided; only disease name was not considered as complete. Define how, where, and when	0. Not documented<br>1. Documented

		identified (e.g., symptoms, results from previous tests, inclusion in registry, patient-care setting, location, dates)	potentially eligible participants or studies were identified. Specify inclusion and exclusion criteria such as location, dates, patient-care setting, symptoms, results from previous tests, or registry inclusion. Indicate whether a consecutive, random, or convenience series was selected. Specify the number of patients, studies, reports, and/or images.	
	9/56%	Data pre-processing steps	#9: not documented, pre-processing documented (but not complete), reproducible pre-processing method documented (fulfilled the following 5 key points), documented that pre-processing not employed. Describe preprocessing steps fully and in sufficient detail so that other investigators could reproduce them. (1) Specify the use of normalization, resampling of image size, change in bit depth, and/or adjustment of window/level settings. (2) State whether or not the data have been rescaled, threshold-limited ("binarized"), and/or standardized. (3) Specify how the following issues were handled: regional format, manual input, inconsistent data, missing data, wrong data types, file manipulations, and missing anonymization. (4) Define any criteria to remove outliers. (5) Specify the libraries, software (including manufacturer name and location), and version numbers, and all option and configuration settings employed.	0. Not documented<br>P. Pre-processing documented<br>NP. Documented that pre-processing not employed
	10/40%	Selection of data subsets, if applicable (for radiomics studies, segmentation is a necessary step)	#10: not documented, image cropping documented, reproducible image cropping method documented. For radiomics studies, segmentation is a necessary step. Describe the tools and parameters used; if done manually, specify the training of the personnel and the criteria they used. Justify how this manual step would be accommodated in the context of the clinical or scientific problem to be solved.	0. Not documented<br>C. Image cropping documented<br>CM. Reproducible image cropping method documented
	11/100%	Definitions of data elements, with references to Common Data Elements	#11: considered as complete, if the predictor and outcome variables is defined. Map them to common data elements, if applicable.	0. Not defined<br>1. Not defined
	12/2%	De-identification methods	#12: not defined, anonymization documented, reproducible anonymization method documented. Describe the methods by which data have been de-identified and how protected health information has been removed.	0. Not documented<br>A. Anonymization documented
	13/17%	How missing data were handled	#13: missing data handling strategy not documented, missing data excluded from analysis, missing data included in analysis.	0. Not documented

			State clearly how missing data were handled, such as replacing them with approximate or predicted values. Describe the biases that the imputed data might introduce.	E. Missing data excluded from analysis<br>I. Missing data included in analysis
**Ground Truth**	14/25%	Definition of ground truth reference standard, in sufficient detail to allow replication	#14: Include detailed, specific definitions of the ground truth annotations, ideally referencing common data elements. Provide an atlas of examples to annotators to illustrate subjective grading schemes (e. g., mild/moderate/severe), and make that information available for review.	0. Not defined<br>1. Defined either explicitly or by reference to a Common Data Element
	15a/3%	Rationale for choosing the reference standard (if alternatives exist)	#15: Describe the rationale for the choice of the reference standard and the potential errors, biases, and limitations of that reference standard.	0. Not documented<br>1. Documented
	15b/50%	Definitive ground truth		0. No definitive ground truth<br>P. Histopathology<br>DI. Definitive imaging modality<br>FU. Case follow-up<br>PFU. Histopathology and case follow-up<br>PDI. Histopathology and definitive imaging modality
	16/40%	Manual image annotation	#16: Specify the number of human annotators and their qualifications. Describe the instructions and training given to annotators; include training materials as a supplement, if possible. Describe whether annotations were done independently and how any discrepancies among annotators were resolved.	0. Not documented<br>UR. Radiologist with unspecified expertise<br>SR. Radiologist with relevant subspecialist expertise<br>OC. Other clinician<br>A. Automatic method
	17/12%	Image annotation tools and software	#17: Specify the software used for manual, semiautomated, or automated annotation, including the version number.	0. Not documented<br>1. Documented
	18/ 16%VM	Measurement of inter- and intra-rater variability; methods to mitigate variability and/or resolve discrepancies	#18: Describe the methods to measure inter- and intra- rater variability, and any steps taken to reduce or mitigate this variability and/or resolve discrepancies. For radiomics studies, this mainly refers to variability between readers, as well we other measurements, such as image interoperations.	0. Not documented<br>V. Variability statistics documented<br>M. Aggregation method documented

				VM. Variability statistics and aggregation method documented
**Data Partitions**	19a/87%	Intended sample size	Describe the sample size and how it was determined. Use traditional power calculation methods, if applicable, to estimate the required sample size to allow for generalizability in a larger population and how many cases are needed to show an effect.	0. Not documented<br>1. Documented number of images in dataset
	19b/1%	Provided power calculation		0. Not documented<br>1. Documented
	19c/72%	Distinct study participants		0. Not documented<br>N. number of study participants
	20/76%	How data were assigned to partitions; specify proportions	#20: Specify how the data were assigned into training, validation ("tuning"), and testing partitions; indicate the proportion of data in each partition and justify that selection. Indicate if there are any systematic differences between the data in each partition, and if so, why.	0. Not documented<br>1. Documented
	21/32%	Level at which partitions are disjoint (e.g., image, study, patient, institution)	#21: Describe the level at which the partitions are disjoint. Sets of medical images generally should be disjoint at the patient level or higher so that images of the same patient do not appear in each partition.	0. Not documented<br>1. Documented partition disjunction at patient level
**Model**	22a	Provided reproducible model description	#22: Provide a complete and detailed structure of the model, including inputs, outputs, and all intermediate layers, in sufficient detail that another investigator could exactly re-construct the network. (1) For neural network models, include all details of pooling, normalization, regularization, and activation in the layer descriptions. Model inputs must match the form of the preprocessed data. Model outputs must correspond to the requirements of the stated clinical problem, and for supervised learning should match the form of the ground truth annotations. If a previously published model architecture is employed, cite a reference that meets the preceding standards and fully describe every modification made to the model. (2) For radiomics studies, this item is considered as complete if all three elements, model type (e.g., logistic regression, Cox proportional hazards model), feature selection procedure to control overfitting, and methods of internal validation (cross-validation, bootstrap sample), were included. A regularization or penalization method such as the	0. Not documented<br>1. Documented
	22b/20%	Provided source code		0. Not documented<br>1. Documented

			least absolute shrinkage and selection operator (LASSO) was considered as both a feature selection procedure and internal validation, as it contains 10-fold cross-validation as a default setting. (3) In some cases, it may be more convenient to provide the structure of the model in code as supplemental data.	
	23/41%	Software libraries, frameworks, and packages	#23: Specify the names and version numbers of all software libraries, frameworks, and packages. Avoid detailed description of hardware unless benchmarking computational performance is a focus of the work.	0. Not documented<br>S. Documented software<br>SV. Documented software and version
	24/68%	Initialization of model parameters (e.g., randomization, transfer learning)	#24: Considered as complete, if feature reduction and selection is described for radiomics models, and the parameters is determined by internal or external validations. Indicate how the parameters of the model were initialized. Describe the distribution from which random values were drawn for randomly initialized parameters. Specify the source of the starting weights if transfer learning is employed to initialize parameters. When there is a combination of random initialization and transfer learning, make it clear which portions of the model were initialized with which strategies.	0. Not documented<br>1. Documented
**Training**	25/ 44%-61%	Details of training approach, including data augmentation, hyperparameters, number of models trained	#25: Completely describe all of the training procedures and hyperparameters in sufficient detail that another investigator could exactly duplicate the training process. This process needed to be performed in a training dataset.	0. Not documented<br>1. Documented
	26/69%	Method of selecting the final model	#26: Describe the method and performance parameters used to select the best-performing model among all the models trained for evaluation against the held-out test set. If more than one model is selected, justify why this is appropriate.	0. Not documented<br>1. Documented model selection criterion, specifying k if k-fold cross validation<br>employed
	27/93%	Ensembling techniques, if applicable	#27: If the final algorithm involves an ensemble of models, describe each model comprising the ensemble in complete detail in accordance with the preceding recommendations. Indicate how the outputs of the component models are weighted and/or combined.	0. Not documented<br>1. Documented<br>n/a. Ensembling not employed
**Evaluation**	28/36%	Metrics of model performance	#28: Describe the metric(s) used to measure the model's performance and indicate how they address the performance characteristics most important to the clinical or scientific	0. Not documented<br>1. Documented

			problem. Compare the presented model to previously published models.	
	29/39%	Statistical measures of significance and uncertainty (e.g., confidence intervals)	#29: Indicate the uncertainty of the performance metrics' values, such as with standard deviation and/or confidence intervals. Compute appropriate tests of statistical significance to compare metrics. Specify the statistical software.	0. Not documented<br>1. Documented
	30/12%	Robustness or sensitivity analysis	#30: Analyze the robustness or sensitivity of the model to various assumptions or initial conditions.	0. Not documented<br>1. Documented
	31/9%	Methods for explainability or interpretability (e.g., saliency maps), and how they were validated	#31: If applied, describe the methods that allow one to explain or interpret the model's results and provide the parameters used to generate them. Describe how any such methods were validated in the current study.	0. Not documented<br>1. Documented
	32/15%	Validation or testing on external data	#32: Describe the data used to evaluate performance of the completed algorithm. When these data are not drawn from a different data source than the training data, note and justify this limitation. If there are differences in structure of annotations or data between the training set and evaluation set, explain the differences, and describe and justify the approach taken to accommodate the differences.	0. Not described<br>I. Employed internal test data<br>E. Employed external test data
**RESULTS**				
**Data**	33/8%	Flow of participants or cases, using a diagram to indicate inclusion and exclusion	#33: Specify the criteria to include and exclude patients or examinations or pieces of information and document the numbers of cases that met each criterion. We strongly recommend including a flowchart/diagram in your results to show initial patient population and those excluded for any reason.	0. Not documented<br>1. Documented
	34/10%	Demographic and clinical characteristics of cases in each partition	#34: Demographic and clinical characteristics of cases in each partition should be specified. State the performance metrics on all data partitions.	0. Documented<br>D. Documented aggregate statistics<br>DP. Documented statistics for each data partition
**Model performance**	35a/49%	Test performance	#35: Report the final model's performance on the test partition.<br>0	0. Not documented<br>V. Performance on validation dataset<br>T. Performance on testing dataset
	35b/17%	Benchmark of performance		0. Not documented

				1. Documented
	36/40%	Estimates of diagnostic accuracy and their precision (such as 95% confidence intervals)	#36: For classification tasks, include estimates of diagnostic accuracy and their precision, such as 95% confidence intervals.	0. Diagnostic performance reported without measure of precision<br>1. Diagnostic performance reported with confidence interval or standard error
	37/24%	Failure analysis of incorrectly classified cases	#37: Provide information to help understand incorrect results. If the task entails classification into two or more categories, provide a confusion matrix that shows tallies for predicted versus actual categories. Consider presenting examples of incorrectly classified cases to help readers better understand the strengths and limitations of the algorithm.	0. Not discussed<br>1. Discussed misclassified cases or model errors
**DISCUSSION**				
**Study limitations**	38	Study limitations, including potential bias, statistical uncertainty, and generalizability	#38: Summarize the results succinctly and place them into context; explain how the current work advances our knowledge and the state of the art. Identify the study's limitations, including those involving the study's methods, materials, biases, statistical uncertainty, unexpected results, and generalizability.	0. Not discussed<br>1. Discussed
**Implications for practice**	39	Implications for practice, including the intended use and/or clinical role	#39: Describe the implications for practice, including the intended use and possible clinical role of the AI model. Describe the key impact the work may have on the field. Envision the next steps that one might take to build upon the results. Discuss any issues that would impede successful translation of the model into practice.	0. Not discussed<br>1. Discussed
**OTHER INFORMATION**				
**Registration**	40/7%	Registration number and name of registry	#40: Comply with the clinical trial registration statement from the International Committee of Medical Journal Editors (ICMJE).	0. Not documented<br>1. Documented
**Study protocol**	41/1%	Where the full study protocol can be accessed	#41: State where readers can access the full study protocol if it exceeds the journal's word limit.	0. Not documented<br>1. Provided access to the full study protocol
**Funding**	42/18%	Sources of funding and other support; role of funders	#42: Specify the sources of funding and other support and the exact role of the funders in performing the study. Indicate whether the authors had independence in each phase of the study.	0. Not documented<br>F. Funding source documented<br>FR. Funding source and role documented

				NF. Stated no funding received

Note: CLAIM = Checklist for Artificial Intelligence in Medical Imaging.
Extracted from Mongan J, Moy L, Kahn CE Jr. Checklist for Artificial Intelligence in Medical Imaging (CLAIM): A Guide for Authors and Reviewers. Radiol Artif Intell. 2020 Mar 25;2(2):e200029

Supplementary Table S5 QUADAS-2 tool for risk of bias and concern on application

Domain and Description	Modified signaling question	Risk of bias	Applicability concern
**Patient selection** - describe methods of patient selection: Describe included patients (prior testing, presentation, intended use of index test and setting)	Signaling question 1: was a consecutive or random sample of patients enrolled?	Could the selection of patients have introduced bias?	Are there concerns that the included patients do not match the review question?
	Signaling question 2: was a case–control design avoided?		
	Signaling question 3: did the study avoid inappropriate exclusions?		
**Index test** - describe the index test and how it was conducted and interpreted	Signaling question 1: were the imaging acquisition protocol, image processing approach described in detail?	Could the conduct or interpretation of the index test have introduced bias?	Are there concerns that the index test, its conduct, or interpretation differ from the review question?
	Signaling question 2: were the segmentation method(s), and feature extraction software described in detail?		
	Signaling question 3: was the validation independent (i. e. external)?		
**Reference standard** - describe the reference standard and how it was conducted and interpreted	Signaling question 1: is the reference standard likely to correctly classify the target condition?	Could the reference standard, its conduct, or its interpretation have introduced bias?	Are there concerns that the target condition as defined by the reference standard does not match the review question?
**Flow and timing** - describe any patients who did not receive the index test(s) and/or reference standard or who were excluded from the 2x2 table (refer to flow diagram): Describe the time interval and any interventions between index test(s) and reference standard	Signaling question 1: was there an appropriate interval between imaging and reference standard?	Could the patient flow have introduced bias?	-

Note: QUADAS-2 = modified Quality Assessment of Diagnostic Accuracy Studies
Extracted from Whiting PF, Rutjes AW, Westwood Me, et al; QUADAS-2 Group. QUADAS-2: a revised tool for the quality assessment of diagnostic accuracy studies. Ann Intern Med. 2011;155(8):529-536.

Supplementary Table S6 Category of five levels of supporting evidence of meta-analyzes

Levels of Supporting Evidence	Description
Convincing	$p < 10^{-6}$, > 1000 events, the largest study reaches statistical significance ($p < 0.05$), I2 < 50%, the null value excluded by the 95% PI, no small-study effects ($p > 0.1$ for Egger's test) and excess significance ($p > 0.1$), and survived the 10% credibility ceiling ($p < 0.05$)
Highly Suggestive	$p < 10^{-6}$, > 1000 events, the largest study reaches statistical significance ($p < 0.05$)
Suggestive	$p < 10^{-3}$, > 1000 events
Weak	$p < 0.05$
Not Suggestive	$p > 0.05$

Note: Extracted from Dang Y, Hou Y. The prognostic value of late gadolinium enhancement in heart diseases: an umbrella review of meta-analyses of observational studies. Eur Radiol. 2021;31(7):4528-4537.

Supplementary Table S7 Study characteristics of included studies

Study	Year	Country	Journal	Impact Factor	Journal Type	First Authorship	Study Design	Imaging Modality	Biomarker	Clinical question
Baidya Kayal 2019	2019	India	Eur J Radiol	3.528	Imaging	Non-radiologist	Prospective	MRI (ADC, IVIM)	Predictive	Response to NAC
Baidya Kayal 2021	2021	India	NMR Biomed	4.044	Imaging	Non-radiologist	Prospective	MRI (T1WI, T2WI, ADC, IVIM)	Predictive	Response to NAC
Baidya Kayal 2022	2022	India	Eur J Radiol	3.528	Imaging	Non-radiologist	Prospective	MRI (T1 mapping)	Predictive	Response to NAC
Bailly 2017	2017	France	PLoS One	3.240	Non-imaging	Radiologist	Retrospective	PET (FDG)	Predictive, Prognosis	Response to NAC, Survival
Chen 2020A	2020	China	Chin J Radiol	n/a	Imaging	Radiologist	Retrospective	MRI (T1WI)	Prognosis	Recurrence
Chen 2020B	2020	China	Eur J Radiol	3.528	Imaging	Radiologist	Retrospective	MRI (T1WI+C)	Prognosis	Recurrence
Chen 2021	2021	China	Eur Radiol	5.315	Imaging	Radiologist	Retrospective	MRI (T1WI+C)	Predictive	Response to NAC
Cho 2019	2019	Korean	PLoS One	3.240	Non-imaging	Radiologist	Retrospective	CT (ROI on nodule)	Diagnostic	Lung nodule malignancy in OS patients
Dai 2020	2020	China	Biomed Res Int	3.411	Non-imaging	Radiologist	Retrospective	MRI (T2WI, T1WI+C)	Diagnostic	OS vs ES
Djuričić 2022	2022	Serbia	J Magn Reson Imaging	4.813	Imaging	Radiologist	Retrospective	MRI (T2WI)	Predictive	Response to NAC
Dufau 2019	2019	France	Bull Cancer	1.276	Non-imaging	Non-radiologist	Retrospective	MRI (T1WI+C)	Predictive; Prognostic	Response to NAC; metastasis
Jeong 2019	2019	Korean	Contrast Media Mol Imagin	3.161	Imaging	Radiologist	Retrospective	PET (FDG)	Predictive	Response to NAC
Kim 2021A	2021	Korean	Cancers (Basel)	6.639	Non-imaging	Radiologist	Retrospective	PET (FDG)	Predictive; Prognostic	Response to NAC; metastasis
Kim 2021B	2021	Korean	Diagnostics (Basel)	3.706	Non-imaging	Non-radiologist	Retrospective	PET (FDG)	Predictive	Response to NAC
Lee 2020	2020	Korean	PLoS One	3.240	Non-imaging	Radiologist	Retrospective	MRI (ADC)	Predictive	Response to NAC

Lin 2020	2020	China	Cancer Imaging	3.909	Imaging	Non-radiologist	Retrospective	CT+C	Predictive	Response to NAC
Liu 2021	2021	China	Biomed Res Int	3.411	Non-imaging	Radiologist	Retrospective	CT	Prognosis	Recurrence
Luo 2022	2022	China	Front Oncol	6.244	Non-imaging	Radiologist	Retrospective	MRI (T1WI, T2WI, T1WI+C)	Prognostic	Lung metastasis
Pereira 2021	2021	Brazil	Br J Radiol	3.039	Imaging	Radiologist	Retrospective	CT	Prognostic	Lung metastasis
Sheen 2019	2019	Korean	PLoS One	3.240	Non-imaging	Radiologist	Retrospective	PET (FDG)	Prognostic	Metastasis
Song 2019	2019	China	Eur Radiol	5.315	Imaging	Radiologist	Retrospective	PET (FDG)	Predictive; Prognostic	Response to NAC; Survival
Wan 2021	2021	China	Med Phys	4.071	Non-imaging	Non-radiologist	Retrospective	CT	Prognostic	Survival
Wu 2018	2018	China	EBioMedicine	8.143	Non-imaging	Non-radiologist	Retrospective	CT	Prognostic	Survival
Xu 2019	2019	China	Phys Med Biol	3.609	Non-imaging	Non-radiologist	Retrospective	CT	Prognostic	Survival
Xu 2021	2021	China	Quant Imaging Med Surg	3.837	Imaging	Non-radiologist	Retrospective	CT (ROI on non-tumorous bone)	Predictive	Response to NAC
Yin 2021	2021	China	Front Oncol	6.244	Non-imaging	Radiologist	Retrospective	CT	Diagnostic	OS vs ES & CS
Zhang 2021	2021	China	Front Oncol	6.244	Non-imaging	Radiologist	Retrospective	MRI (DCE)	Predictive	Response to NAC
Zhao 2019	2019	China	J Bone Oncol	3.500	Non-imaging	Radiologist	Retrospective	MRI (DWI)	Prognostic	Survival
Zhong 2022	2022	China	Eur Radiol	5.315	Imaging	Radiologist	Retrospective	MRI (T2WI)	Predictive	Response to NAC

Note: NAC = neoadjuvant chemotherapy, OS = osteosarcoma, ES = Ewing sarcoma, CS = chondrosarcoma, DCE = dynamic contrast- enhanced, DWI = diffusion weighted imaging.

Supplementary Table S8 PICOT of included studies

Study	Sample Size	Institution	Inclusion Period	Patient Condition	Gender (F/M)	Age	Comparing Test	Reference Standard	Outcome	Timing
Baidya Kayal 2019	40	L; SC	March 2016 to March 2019	needle biopsy proven osteosarcoma	10/30	17.7 ± 5.9 y/o	None	RECIST	Response to NAC	3 time points: pre-NACT (t0), after 1st NACT (t1, 2–3 weeks) and after 3rd NACT (t2, 8–9 weeks)
Baidya Kayal 2021	40	L; SC	March 2016 to March 2019	needle biopsy proven osteosarcoma	9/31	17.2 ± 5.7 y/o	None	Histology	Response to NAC	3 time points: at baseline (t0), after the first NACT (t1) and after the third NACT (t2)
Baidya Kayal 2022	35	L; SC	March 2016 to March 2019	needle biopsy proven osteosarcoma	8/27	17.9 ± 6 y/o	None	Histology	Response to NAC	2 time points: before NACT (baseline) and after NACT completion (follow-up)
Bailly 2017	31	L; SC	2004 to 2014	histological proven osteosarcoma	18/13	12.8 ± 2.9 y/o	None	RECIST; Follow-up with imaging	Response to NAC, Survival	2 time points: baseline value, and the post-chemotherapy value
Chen 2020A	107	L; MC	Jan 2009 to Oct 2017	histological proven osteosarcoma	38/69	22.6 ± 12.4 y/o	Radiologist's interpretation	Follow-up with imaging	Recurrence	1 time point: pre-treatment
Chen 2020B	93	L; MC	Jan 2009 to Oct 2017	histological proven osteosarcoma	34/59	5–86 y/o	Radiologist's interpretation	Follow-up with imaging	Recurrence	1 time point: pre-treatment
Chen 2021	102	L; MC	Feb 2009 and Jan 2019	histological proven osteosarcoma	T: 29/39; V: 12/22	T: 21.81 ± 13.46 y/o; V: 20.76 ± 8.63 y/o	Radiologist's interpretation	Histology	Response to NAC	1 time point: pre-treatment

Cho 2019	42 pulmonary nodules in 16 patients	L; SC	Jan 2009 to Dec 2014	pathologically proven pulmonary nodules in histological proven osteosarcoma	6/10	13.8 y/o	None	Histology	Lung nodule malignancy in OS patients	1 time point: preoperative
Dai 2020	35 OS; 31 ES	L; SC	Apr 2013 to Dec 2017	histologically confirmed cases	OS: 16/19; ES: 10/21	OS: 30:7 ± 16:5 y/o; ES: 24:5 ± 9:6 y/o	None	Histology	OS vs ES	1 time point: details not documented
Djuričić 2022	54	L; SC	2010 to 2014	histological proven osteosarcoma	19/35	Median, 14 y/o for female, 16 y/o for male	None	Histology	Response to NAC	1 time point: pre-NAC
Dufau 2019	69	L; MC	Jan 2007 to Dec 2016	histological proven osteosarcoma	35/34	Median 15.7 y/o	None	Histology; Imaging	Response to NAC; metastasis	1 time point: details not documented
Jeong 2019	70	L; SC	Jun 2016 to May 2017	histological proven osteosarcoma	not documented	not documented	None	Histology	Response to NAC	2 time points: at baseline, and after NAC
Kim 2021A	52	L; SC	not documented	histological proven osteosarcoma	21/31	All ≤ 14 y/o	None	Histology; not documented	Response to NAC; metastasis	1 time point: pre-NAC
Kim 2021B	105	L; SC	Jun 2006 to May 2014	histological proven osteosarcoma	30/75	77.14% ≤ 19 y/o	None	Histology	Response to NAC	2 time points: pre-NAC, and after NAC
Lee 2020	17	L; SC	Mar 2009 to May 2017	histological proven osteosarcoma	4/13	17 y/o	Radiologist's interpretation	Histology	Response to NAC	1 time point: post-NAC
Lin 2020	191	L; SC	Nov 2013 to Nov 2017	histological proven osteosarcoma	T: 23/34 for pGR; 33/47 for non-pGR; V: 11/14 for pGR; 16/13 for non-pGR	Median, T: 16 for pGR; 14 for non-pGR; V: 15 for pGR, 18 for non-pGR	None	Histology	Response to NAC	2 time points: pre-NAC, and after NAC

Liu 2021	80	L; MC	Aug 2021 to Dec 2018	histological proven osteosarcoma	49/31	25:59 ± 15:74 y/o	Clinical model	Follow-up with imaging	Recurrence	1 time point: at diagnosis
Luo 2022	78	L; SC	Jan 2014 to Dec 2020	histological proven osteosarcoma	SLM: 15/30; non-SLM: 14/19	SLM: 19.49 ± 13.86; non-SLM: 16.45 ± 7.53 y/o	None	Follow-up with imaging	Lung metastasis	1 time point: pre-treatment
Pereira 2021	81	L; SC	Jan 2012 to Jun 2018	histological proven osteosarcoma	LM: 17/34; non-LM: 15/15	LM: 22 ± 12 y/o; non-LM: 21 ± 14 y/o	None	Follow-up with imaging	Lung metastasis	1 time point: pre-treatment
Sheen 2019	83	L; SC	Jun 2006 to Aug 2012	histological proven osteosarcoma	23/60	80.72% ≤ 19 y/o	None	Follow-up with imaging	Metastasis	1 time point: pre-treatment
Song 2019	35	L; SC	Jan 2013 to Dec 2017	histological proven osteosarcoma	15/20	Median, 33 y/o	None	Histology; Follow-up with imaging	Response to NAC; Survival	1 time point: pre-treatment
Wan 2021	150	L; SC	Jan 2008 to Apr 2012	histological proven osteosarcoma	76/74	Median 15 y/o	None	Follow-up with imaging	Survival	1 time point: at diagnosis
Wu 2018	150	L; SC	Jan 2008 to Apr 2012	histological proven osteosarcoma	76/74	Median 15 y/o	Clinical model	Follow-up with imaging	Survival	1 time point: at diagnosis
Xu 2019	150	L; SC	Jan 2008 to Apr 2012	histological proven osteosarcoma	76/74	Median 15 y/o	None	Follow-up with imaging	Survival	1 time point: at diagnosis
Xu 2021	157	L; SC	Nov 2013 to Nov 2017	histologically confirmed cases	pGR: 28/41; non-pGR: 38/51	pGR: 17.65 ± 7.57 y/o; non-pGR: 16.48 ± 7.30 y/o	None	Histology	Response to NAC	1 time point: post-NAC
Yin 2021	81 ES; 106 OS; 127 CS	L; SC	Apr 2006 to Dec 2019	histologically confirmed cases	ES: 26/55; OS: 47/59; CS: 60/67	Median, ES: 17.00; OS: 26.00; CS: 44.00	None	Histology	OS vs ES & CS	1 time point: details not documented

Zhang 2021	102	L; SC	Jan 2016 and May 2020	histological proven osteosarcoma	42/60	17 ± 9.77 y/o	None	RECIST	Response to NAC	2 time points: within 1 week before the NAC implementation and at the end of the two cycles of NAC
Zhao 2019	112	L; SC	Jan 2012 to Dec 2017	histological proven osteosarcoma	T: 36/49; V: 12/15	T: median 18; V: median 17.5	Clinical model	Follow-up with imaging	Survival	1 time point: pre-treatment
Zhong 2022	144	L; SC	Mar 2016 to Aug 2019	histological proven osteosarcoma	T: 11/14 for pGR; 23/53 for non-pGR; V: 7/4 for pGR; 10/22 for non-pGR	T: 22.5 ± 13.2 for pGR; 17.9 ± 10.1 for non-pGR; V: 22.8 ± 12.0 for pGR, 19.5 ± 11.4 for non-pGR	Clinical model	Histology	Response to NAC	1 time point: post-NAC

Note: PICOT = population, intervention, control, outcome and timing. L = Local data collection, P = Public data, LP = Both local and public data; SC = Single-center data, MC = Multi-center data, SLM = synchronous lung metastases, NAC = neoadjuvant chemotherapy.

Supplementary Table S9 Radiomics methodological issue of included studies

Study	Imaging	ROI segmentation	Radiomics feature extraction	Non-radiomics features	Feature reduction and selection	Classifier/ Evaluation	Outcome	Validation	Model type
Baidya Kayal 2019	MRI (ADC, IVIM)	CM, 1 SR, manual; not documented	MATLAB, v2017	None	paired *t*-test; one-way ANOVA	ROC curve analysis	Response to NAC	None	1a
Baidya Kayal 2021	MRI (T1WI, T2WI, ADC, IVIM)	CM, 1 SR, manual; not documented	MATLAB	None	(1) classification accuracy of each individual feature; (2) classification accuracy of selected features in combination that belongs to a particular TA method; (3) classification accuracy of all selected features in combination from all three TA methods.	ROC curve analysis	Response to NAC	8-fold cross-validation	1b
Baidya Kayal 2022	MRI (T1 mapping)	CM, 1 SR, manual; not documented	MATLAB, v2017	None	Mann–Whitney *U* test	ROC curve analysis	Response to NAC	None	1a
Bailly 2017	PET (FDG)	CM, 2 URs, semi-automatic; PLANE- T Onco-Solution	Not documented	Bone metastasis, lung metastasis	univariate and multivariate Cox proportional hazard regression model	univariate and multivariate Cox proportional hazard regression model	Response to NAC, Survival	None	1a
Chen 2020A	MRI (T1WI)	CM, 2 SRs, manual; ITK-SNAP	MATLAB, v2016b; R; Pyradiomics	Radiologic feature	Spearman correlation; LASSO	LASSO	Recurrence	External, 2 other centers	3
Chen 2020B	MRI (T1WI+C)	CM, 2 SRs, manual; ITK-SNAP	MATLAB, v2016b; R	Radiologic feature	Spearman correlation; LASSO	LASSO	Recurrence	External, 2 other centers	3
Chen 2021	MRI (T1WI+C)	CM, 2 SRs, manual; ITK-SNAP, v3.6.0	Python, v3.6.8; R, v3.5.1; Pyradiomics	None	Pearson correlation	LASSO-LR; SVM; GP; NB;	Response to NAC	External, 2 other centers	3

Cho 2019	CT (ROI on nodule)	CM, 2 SRs, manual; Medical Imaging Solution for Segmentation and Texture Analysis	Medical Imaging Solution for Segmentation and Texture Analysis, on C++ language	Radiologic feature	independent t test; LR	ROC curve analysis	Lung nodule malignancy in OS patients	None	1a
Dai 2020	MRI (T2WI, T1WI+C)	CM, 2 SRs, manual; ITK-SNAP, v3.6.0	Analysis Kit	None	independent-sample t-test, Spearman's test, LASSO	LASSO	OS vs ES	10-fold cross-validation	1b
Djuričić 2022	MRI (T2WI)	CM, 2 SRs, manual; Fuji/ImageJ2, v v29.2.1.2	Fuji/ImageJ2, v v29.2.1.2	None	LASSO	LASSO, LR	Response to NAC;	k-fold cross-validation	1b
Dufau 2019	MRI (T1WI+C)	CM; 1 UR, manual; not documented	MATLAB	None	independent t test	SVM; PCA	Response to NAC; metastasis	Randomly split	2a
Jeong 2019	PET (FDG)	C, region-growing algorithm + 1 UR, automatic + manual; not documented	Chang-Gung Image Texture Analysis (open-source software)	None	Mann–Whitney *U* test, ROC analysis, logistic analysis	SVM, RF, GB	Response to NAC	10-fold cross-validation	1b
Kim 2021A	PET (FDG)	not documented	LiFEx, v4.0	*KI67* and *EZRIN* expression	ROC analysis	RF, GB	Response to NAC; metastasis	10-fold cross-validation	1b
Kim 2021B	PET (FDG)	CM, region growing method based on SUV ≥1.5, automatic	LiFEx, v4.0	Deep learning feature	ROC analysis	RF, SVM	Response to NAC	10-fold cross-validation	1b
Lee 2020	MRI (ADC)	CM, 2 SRs, manual; not documented	MR OncoTreat	Radiologists' opinion	Mann–Whitney *U* test	ROC curve analysis	Response to NAC	None	1a
Lin 2020	CT+C	CM, 2 OCs, manual; ITK-SNAP	open-source Radiomics packages,	pulmonary metastases	intra-class correlation coefficient, Pearson correlation analysis, Mann-Whitney U test, LASSO	LASSO	Response to NAC	Non-randomly split	2b

			MATLAB, v2016						
Liu 2021	CT	CM, 2 SRs, manual; ITK-SNAP	MATLAB, v2015b	Hemoglobin, joint invasion	mRMR, LASSO	LASSO	Recurrence	External, 2 other centers	3
Luo 2022	MRI (T1WI, T2WI, T1WI+C)	CM; 2 URs, manual; ITK-SNAP, v3.8.0	Analysis Kit, Pyradiomics, v3.0	Clinical feature	LASSO	LR, SVM	Lung metastasis	Randomly split	2a
Pereira 2021	CT	CM, 3 SRs semi-automatic; 3D-slicer, v4.10.2	Pyradiomics, v.2.2.0; Weka, v3.8.4	None	intra-class correlation coefficient, correlation-based-features selection	RF, NB, Multilayer perceptron	Lung metastasis	Randomly split	2a
Sheen 2019	PET (FDG)	CM, region growing method based on SUV ≥2.0, automatic	LiFEx, v4.0	None	Spearman correlation, Akaike's Information Criterion	ROC curve analysis	Metastasis	Randomly split	2a
Song 2019	PET (FDG)	C, 1 UR, manual; ITK-SNAP, v3.6.0	Pyradiomics	Clinical feature	Student's t test	ROC curve analysis; Cox regression analyis; Kaplan-Meier curves	Response to NAC; Survival	None	1a
Wan 2021	CT	CM; 2 URs, manual; ITK-SNAP, v3.6.0	MATLAB, v2018a	sparse autoencoder feature (deep learning feature)	Mann–Whitney *U* test, mRMR,	SVM	Survival	Randomly split	2a
Wu 2018	CT	CM, 3 OCs, manual; ITK-SNAP	MATLAB, v2015b	Clinical feature	intra-class correlation coefficient, Spearman correlation, LASSO	LASSO	Survival	Randomly split	2a
Xu 2019	CT	CM, 3 OCs, manual; ITK-SNAP	MATLAB, v2017b	None	mRMR, LASSO, relief, gini index	LR, ANN, SVM, NB	Survival	4-fold cross-validation	1b

Xu 2021	CT (ROI on non-tumorous bone)	CM, 2 OCs + 1UR, manual; ITK-SNAP	MATLAB, v2017b	Clinical feature	LOOCV	LR	Response to NAC	Randomly split	2a
Yin 2021	CT	CM, 2 SRs, manual; MITK, v 2018.04.2	Artificial Intelligence Kit, v3.3.0	Clinical feature	Spearman correlation; GBDT	RF	OS vs ES & CS	Randomly split	2a
Zhang 2021	MRI (DCE)	CM, 2 SRs, manual; ITK-SNAP, v3.8.0	Radcloud	Clinical feature	SelectKBest, LASSO	KNN, LR, SVM	Response to NAC	Randomly split	2a
Zhao 2019	MRI (DWI)	CM, 3 OCs manual; MIM	IBEX; R, v3.6.3	Clinical feature	LASSO	LASSO	Survival	Randomly split	2a
Zhong 2022	MRI (T2WI)	CM, 2 SRs, manual; nnU-Net, automatic; MultiLabel, v1.0	FeAture Explorer, v0.3.6; Pyradiomics, v3.0; R, v4.1.0	Clinical feature	Pearson correlation; ANOVA, relief, RFE	LR, SVM	Response to NAC	Randomly split	2a

Note: C = Image cropping documented, CM = Reproducible image cropping method documented; UR = Radiologist with unspecified expertise, SR = Radiologist with relevant subspecialist expertise, OC = Other clinician, LOOCV = Leave-one-out cross validation, mRMR = maximum relevance minimum redundancy, GBDT = gradient boosting decision tree, LASSO = least absolute shrinkage and selection operator, LR = logistic regression, SVM = support vector machine, GP = Gaussian process, NB = Naive Bayes, GB = gradient boosting, RF = random forest, ANN = artificial neural network, ANOVA = analysis of variance, RFE = recursive feature elimination, K-nearest neighbor (KNN).

Supplementary Table S10 Model presentation and performance metrics of included studies

Study	Full prediction model	Nomogram, calculator, etc.	Biologic correlation	Discrimination statistics	Calibration statistics	Cost-effectiveness analysis	Potential clinical utility
Baidya Kayal 2019	N	None	microcapillary perfusion	AUC, no confidential interval	N	N	None
Baidya Kayal 2021	Y	None	microcapillary perfusion, tumor heterogeneity	AUC	N	N	None
Baidya Kayal 2022	N	None	tumor heterogeneity	AUC	N	N	None
Bailly 2017	N	None	glycolytic activity	AUC, no confidential interval	N	N	None
Chen 2020A	Y	Nomogram or formula	tumor heterogeneity	AUC	Calibration curve	N	DCA
Chen 2020B	Y	Nomogram or formula	tumor heterogeneity	AUC	Calibration curve	N	DCA
Chen 2021	Y	Nomogram or formula	tumor heterogeneity	AUC	Calibration curve	N	DCA
Cho 2019	N	None	tumor heterogeneity	AUC, no confidential interval	N	N	None
Dai 2020	Y	None	tumor heterogeneity	AUC	N	N	None
Djuričić 2022	Y	None	increased structural irregularity/ complexity predicted higher chemoresistance	AUC	N	N	None
Dufau 2019	N	None	angiogenèse, agressivité tumorale élevée, chimio sensibilité aux antimitotiques	AUC	N	N	None
Jeong 2019	N	None	tumor heterogeneity	AUC, no confidential interval	N	N	None

Kim 2021A	N	None	tumor heterogeneity	AUC, no confidential interval	N	N	None
Kim 2021B	N	None	tumor heterogeneity	AUC, no confidential interval	N	N	None
Lee 2020	N	None	tumor heterogeneity	AUC, no confidential interval	N	N	None
Lin 2020	Y	Nomogram or formula	tumor heterogeneity	AUC	Calibration curve	N	DCA
Liu 2021	Y	Nomogram or formula	tumor heterogeneity	AUC	Calibration curve	N	DCA
Luo 2022	Y	Nomogram or formula	tumor vascular permeability, tumor heterogeneity	AUC	Calibration curve	N	DCA
Pereira 2021	Y	None	None	AUC	N	N	None
Sheen 2019	Y	Nomogram or formula	tumor heterogeneity	AUC, no confidential interval	Hosmer-Lemeshow test result	N	None
Song 2019	N	None	tumor heterogeneity	AUC	N	N	None
Wan 2021	Y	None	tumor heterogeneity	AUC	N	N	None
Wu 2018	Y	Nomogram or formula	tumor heterogeneity	AUC	Calibration curve	N	DCA
Xu 2019	Y	None	tumor heterogeneity	AUC	N	N	None
Xu 2021	Y	None	bone microenvironment and complex bone cell-tumor interactions	AUC	N	N	None
Yin 2021	Y	None	None	AUC, no confidential interval	N	N	None
Zhang 2021	Y	Nomogram or formula	tumor heterogeneity	AUC	N	N	None
Zhao 2019	Y	Nomogram or formula	tumor heterogeneity	AUC	N	N	None

Zhong 2022	Y	Nomogram or formula	tumor size	AUC	Calibration curve	N	DCA

Note: Y = yes, N = no, DCA = decision curve analysis.

Supplementary Table S11 RQS rating per study

Study	Baidya Kayal [illegible]	Baidya Kayal [illegible]	Baidya Kayal [illegible]	Bailly 2017	Chen 2020A	Chen 2020B	Chen 2021	Cho 2019	Dai 2020	Djuričić 2022	Dufau 2019	Jeong 2019	Kim 2021A	Kim 2021B	Lee 2020	Lin 2020	Liu 2021	Luo 2022	Pereira 2021	Sheen 2019	Song 2019	Wan 2021	Wu 2018	Xu 2019	Xu 2021	Yin 2021	Zhang 2021	Zhao 2019	Zhong 2022
**Total 16 items (ideal score 36)**	3	11	4	3	17	18	17	3	10	10	8	10	9	11	5	13	18	13	10	11	3	10	16	9	11	9	12	14	17
**Domain 1: protocol quality and stability in image and segmentation (0 to 5 points)**	2	2	2	2	1	2	2	2	2	2	0	2	0	2	2	2	3	2	2	1	1	1	2	1	2	2	3	1	2
**Protocol quality (2 points)**	1	1	1	0	0	1	1	1	1	1	0	1	0	1	1	1	1	1	1	1	1	0	1	0	1	1	1	0	1
**Multiple segmentations (1 point)**	0	0	0	1	1	1	1	1	1	1	0	0	0	0	1	1	1	1	1	0	0	1	1	1	1	1	1	1	1
**Test-retest (1 point)**	1	1	1	1	0	0	0	0	0	0	0	1	0	1	0	0	1	0	0	0	0	0	0	0	0	0	1	0	0
**Phantom study (1 point)**	0	0	0	0	0	0	0	0	0	0	0	0	0	0	0	0	0	0	0	0	0	0	0	0	0	0	0	0	0
**Domain 2: feature selection and validation (-8 to 8 points)**	-8	-1	-8	-2	7	7	7	-2	5	5	5	5	5	5	-2	5	8	5	5	5	-2	5	5	5	5	5	5	5	5
**Feature reduction or**	-3	-3	-3	3	3	3	3	3	3	3	3	3	3	3	3	3	3	3	3	3	3	3	3	3	3	3	3	3	3

**adjustment of multiple testing (-3 or 3 points)**																													
**Validation (-5, 2, 3, 4, or 5 points)**	-5	2	-5	-5	4	4	4	-5	2	2	2	2	2	2	-5	2	5	2	2	2	-5	2	2	2	2	2	2	2	2
**Domain 3: biologic/clinical validation and utility (0 to 6 points)**	1	1	1	2	5	5	4	2	1	1	1	1	2	2	4	3	5	3	0	1	2	2	5	1	2	1	2	4	5
**Non-radiomics features (1 point)**	0	0	0	1	1	1	0	1	0	0	0	0	1	1	1	1	1	1	0	0	1	1	1	0	1	1	1	1	1
**Biologic correlations (1 point)**	1	1	1	1	1	1	1	1	1	1	1	1	1	1	1	1	1	1	0	1	1	1	1	1	1	0	1	1	1
**Comparison to "gold standard" (2 points)**	0	0	0	0	2	2	2	0	0	0	0	0	0	0	2	0	2	0	0	0	0	0	2	0	0	0	0	2	2
**Potential clinical utility (2 points)**	0	0	0	0	1	1	1	0	0	0	0	0	0	0	0	1	1	1	0	0	0	0	1	0	0	0	0	0	1
**Domain 4: model performance index (0 to 5 points)**	1	2	2	1	4	4	4	1	2	2	2	1	1	1	1	2	2	2	2	3	2	2	4	2	2	1	2	2	4
**Cut-off analysis (1 point)**	0	0	0	0	0	0	0	0	0	0	0	0	0	0	0	0	0	0	0	0	0	0	0	0	0	0	0	0	0
**Discrimination statistics (2 points)**	1	2	2	1	2	2	2	1	2	2	2	1	1	1	1	2	2	2	2	1	2	2	2	2	2	1	2	2	2

**Calibration statistics (2 points)**	0	0	0	0	2	2	2	0	0	0	0	0	0	0	0	0	0	0	0	2	0	0	2	0	0	0	0	0	2
**Domain 5: high level of evidence (0 to 8 points)**	7	7	7	0	0	0	0	0	0	0	0	0	0	0	0	0	0	0	0	0	0	0	0	0	0	0	0	0	0
**Prospective study (7 points)**	7	7	7	0	0	0	0	0	0	0	0	0	0	0	0	0	0	0	0	0	0	0	0	0	0	0	0	0	0
**Cost-effectiveness analysis (1 point)**	0	0	0	0	0	0	0	0	0	0	0	0	0	0	0	0	0	0	0	0	0	0	0	0	0	0	0	0	0
**Domain 6: Open science and data (0 to 4 points)**	0	0	0	0	0	0	0	0	0	0	0	1	1	1	0	1	0	1	1	1	0	0	0	0	0	0	0	2	1

Note: RQS = Radiomics Quality Score.
Extracted from Lambin P, Leijenaar RTH, Deist TM, et al. Radiomics: the bridge between medical imaging and personalized medicine. Nat Rev Clin Oncol. 2017;14(12):749-762.

Supplementary Table S12 TRIPOD adherence per study

Study	Baidya Kayal 2019	Baidya Kayal 2021	Baidya Kayal 2022	Bailly 2017	Chen 2020A	Chen 2020B	Chen 2021	Cho 2019	Dai 2020	Djuričić 2022	Dufau 2019	Jeong 2019	Kim 2021A	Kim 2021B	Lee 2020	Lin 2020	Liu 2021	Luo 2022	Pereira 2021	Sheen 2019	Song 2019	Wan 2021	Wu 2018	Xu 2019	Xu 2021	Yin 2021	Zhang 2021	Zhao 2019	Zhong 2022
**Overall**	13	16	13	11	18	20	21	13	17	18	18	12	13	14	14	19	18	20	18	12	14	15	22	15	20	17	20	17	23
**Title and Abstract**	0	0	0	0	0	2	1	0	0	0	1	0	1	1	0	1	1	1	1	1	0	0	1	1	2	1	0	1	1
**1**	0	0	0	0	0	1	0	0	0	0	0	0	0	0	0	0	0	0	0	0	0	0	0	0	1	0	0	0	0
**2**	0	0	0	0	0	1	1	0	0	0	1	0	1	1	0	1	1	1	1	1	0	0	1	1	1	1	0	1	1
**Introduction**	1	1	1	1	2	1	2	1	1	2	2	1	1	1	1	2	1	1	1	2	1	1	2	1	1	1	1	1	1
**3a**	1	1	1	1	1	1	1	1	1	1	1	1	1	1	1	1	1	1	1	1	1	1	1	1	1	1	1	1	1
**3b**	0	0	0	0	1	0	1	0	0	1	1	0	0	0	0	1	0	0	0	1	0	0	1	0	0	0	0	0	0
**Methods**	7	7	7	5	8	8	9	6	9	8	7	6	5	6	7	7	7	9	8	4	8	7	10	7	9	8	10	7	12
**4a**	P	P	P	R	R	R	R	R	R	R	R	R	R	R	R	R	R	R	R	R	R	R	R	R	R	R	R	R	R
**4b**	L	L	L	L	L	L	L	L	L	L	L	L	L	L	L	L	L	L	L	L	L	L	L	L	L	L	L	L	L
**5a**	SC	SC	SC	SC	MC	MC	MC	SC	SC	MC	MC	SC	SC	SC	SC	SC	MC	SC	SC	SC	SC	SC	SC	SC	SC	SC	SC	SC	SC
**5b**	1	1	1	0	1	1	1	1	1	1	1	0	0	0	1	1	1	1	1	0	1	0	1	1	1	1	1	0	1
**5c, if relevant (N = 25)**	0	0	0	0	1	1	1	n/a	n/a	1	1	0	1	1	1	1	1	n/a	0	0	1	0	1	0	1	n/a	1	1	1
**6a**	1	1	1	1	1	1	1	1	1	1	1	1	0	1	1	1	1	1	1	0	1	1	1	1	1	1	1	1	1
**6b**	0	0	1	0	0	0	0	0	0	0	0	0	0	0	0	0	0	0	0	0	0	0	0	0	1	0	0	0	1
**7a**	0	0	0	0	0	0	1	0	1	1	0	0	0	0	0	0	0	1	1	0	1	1	0	0	0	1	1	0	1
**7b**	0	0	0	0	0	0	0	0	0	0	0	0	0	0	1	0	0	0	0	0	1	0	0	0	0	0	1	0	1
**8**	0	1	0	0	0	0	0	0	0	0	0	0	0	0	0	0	0	0	0	0	0	0	1	0	0	0	0	1	0
**9**	0	0	0	0	0	0	0	0	E	0	0	0	0	0	0	0	0	E	0	0	0	0	E	0	E	0	E	0	E
**10a**	1	1	1	1	1	1	1	1	1	1	1	1	1	1	1	1	1	1	1	1	1	1	1	1	1	1	1	1	1
**10b**	0	0	0	0	1	1	1	0	1	1	1	1	1	1	0	1	1	1	1	0	0	1	1	1	1	1	1	1	1
**10d**	1	0	0	0	1	1	1	0	0	0	0	0	0	0	0	0	0	0	0	0	0	0	1	0	0	0	0	0	1

**11, if done (N = 0)**	n/a	n/a	n/a	n/a	n/a	n/a	n/a	n/a	n/a	n/a	n/a	n/a	n/a	n/a	n/a	n/a	n/a	n/a	n/a	n/a	n/a	n/a	n/a	n/a	n/a	n/a	n/a	n/a	n/a
**Results**	1	4	1	1	5	6	6	2	4	5	5	2	3	3	2	6	6	6	5	3	2	4	6	3	5	4	6	5	6
**13a**	0	1	0	0	0	1	1	1	0	1	1	0	0	0	1	1	1	1	1	0	0	0	1	0	1	1	1	0	1
**13b**	1	1	0	1	1	1	1	1	1	1	1	0	1	1	1	1	1	1	1	1	1	1	1	0	1	1	1	1	1
**14a**	0	1	0	0	1	1	1	0	1	1	1	1	1	1	0	1	1	1	1	1	0	1	1	1	1	1	1	1	1
**14b, if done (N = 5)**	n/a	n/a	n/a	0	n/a	n/a	n/a	1	n/a	n/a	n/a	n/a	n/a	n/a	n/a	n/a	1	n/a	n/a	n/a	1	n/a	n/a	n/a	n/a	n/a	n/a	1	n/a
**15a**	0	0	0	0	1	1	1	0	1	1	1	1	1	1	0	1	1	1	1	0	0	1	1	1	1	1	1	1	1
**15b**	0	0	0	0	1	1	1	0	0	0	0	0	0	0	0	1	1	1	0	1	0	0	1	0	0	0	1	1	1
**16**	0	1	1	0	1	1	1	0	1	1	1	0	0	0	0	1	1	1	1	0	1	1	1	1	1	0	1	1	1
**Discussion**	3	3	3	3	3	3	3	3	3	3	3	3	3	3	3	3	3	3	3	2	3	3	3	3	3	3	3	3	3
**18**	1	1	1	1	1	1	1	1	1	1	1	1	1	1	1	1	1	1	1	0	1	1	1	1	1	1	1	1	1
**19b**	1	1	1	1	1	1	1	1	1	1	1	1	1	1	1	1	1	1	1	1	1	1	1	1	1	1	1	1	1
**20**	1	1	1	1	1	1	1	1	1	1	1	1	1	1	1	1	1	1	1	1	1	1	1	1	1	1	1	1	1
**Other information**	1	1	1	1	0	0	0	1	0	0	0	0	0	0	1	0	0	0	0	0	0	0	0	0	0	0	0	0	0
**21**	0	0	0	0	0	0	0	0	0	0	0	0	0	0	0	0	0	0	0	0	0	0	0	0	0	0	0	0	0
**22**	NF	NF	NF	FR	F	F	F	NF	0	0	0	F	F	F	FR	F	F	F	0	F	F	F	F	F	F	F	0	F	F
**Validation (N = 16)**	n/a	n/a	n/a	n/a	3	3	3	n/a	n/a	n/a	0	n/a	n/a	n/a	n/a	3	3	3	1	1	n/a	1	2	n/a	1	1	2	2	3
**Model type**	1a	1b	1a	1a	3	3	3	1a	1b	1b	2a	1b	1b	1b	1a	2b	3	2a	2a	2a	1a	2a	2a	1b	2a	2a	2a	2a	2a
**10c**	n/a	n/a	n/a	n/a	1	1	1	n/a	n/a	n/a	0	n/a	n/a	n/a	n/a	1	1	1	1	1	n/a	1	1	n/a	1	1	1	1	1
**10e, if done**	n/a	n/a	n/a	n/a	n/a	n/a	n/a	n/a	n/a	n/a	n/a	n/a	n/a	n/a	n/a	n/a	n/a	n/a	n/a	n/a	n/a	n/a	n/a	n/a	n/a	n/a	n/a	n/a	n/a
**12**	n/a	n/a	n/a	n/a	1	1	1	n/a	n/a	n/a	0	n/a	n/a	n/a	n/a	1	1	1	0	0	n/a	0	1	n/a	0	0	1	1	1
**13c**	n/a	n/a	n/a	n/a	0	0	0	n/a	n/a	n/a	0	n/a	n/a	n/a	n/a	0	0	1	0	0	n/a	0	0	n/a	0	0	0	0	1
**17, if done**	n/a	n/a	n/a	n/a	n/a	n/a	n/a	n/a	n/a	n/a	n/a	n/a	n/a	n/a	n/a	n/a	n/a	n/a	n/a	n/a	n/a	n/a	n/a	n/a	n/a	n/a	n/a	n/a	n/a

**19a**	n/a	n/a	n/a	n/a	1	1	1	n/a	n/a	n/a	0	n/a	n/a	n/a	n/a	1	1	0	0	0	n/a	0	0	n/a	0	0	0	0	0

Note: TRIPOD = Transparent Reporting of a multivariable prediction model for Individual Prognosis Or Diagnosis.
Extracted from Collins GS, Reitsma JB, Altman DG, Moons KG. Transparent reporting of a multivariable prediction model for individual prognosis or diagnosis (TRIPOD): the TRIPOD statement. Ann Intern Med. 2015;162(1):55-63.

Supplementary Table S13 CLAIM adherence per study

Study	Baidya Kayal 2019	Baidya Kayal 2021	Baidya Kayal 2022	Bailly 2017	Chen 2020A	Chen 2020B	Chen 2021	Cho 2019	Dai 2020	Djuričić 2022	Dufau 2019	Jeong 2019	Kim 2021A	Kim 2021B	Lee 2020	Lin 2020	Liu 2021	Luo 2022	Pereira 2021	Sheen 2019	Song 2019	Wan 2021	Wu 2018	Xu 2019	Xu 2021	Yin 2021	Zhang 2021	Zhao 2019	Zhong 2022
**Overall**	24	32	27	22	36	40	43	26	33	33	31	23	23	28	26	39	42	41	40	32	24	33	39	28	37	37	42	36	44
**Title and Abstract**	2	1	2	2	2	2	2	2	2	2	2	2	1	1	2	2	2	2	2	2	2	2	1	1	2	2	2	2	2
**1**	1	1	1	1	1	1	1	1	1	1	1	1	1	1	1	1	1	1	1	1	1	1	1	1	1	1	1	1	1
**2**	1	0	1	1	1	1	1	1	1	1	1	1	0	0	1	1	1	1	1	1	1	1	0	0	1	1	1	1	1
**Introduction**	1	2	3	2	2	2	3	2	2	2	2	1	2	1	2	2	2	2	2	2	2	1	2	2	2	2	2	1	2
**3**	1	1	1	1	1	1	1	1	1	1	1	1	1	1	1	1	1	1	1	1	1	1	1	1	1	1	1	1	1
**4a**	0	1	1	1	1	1	1	1	1	1	1	0	1	0	0	1	1	1	1	1	1	0	1	1	0	1	1	0	1
**4b**	0	0	1	0	0	0	1	0	0	0	0	0	0	0	1	0	0	0	0	0	0	0	0	0	1	0	0	0	0
**Methods**	17	23	18	14	26	29	31	17	25	25	21	18	17	23	16	29	30	31	30	25	16	24	29	22	27	27	31	27	32
**5**	P	P	P	R	R	R	R	R	R	R	R	R	R	R	R	R	R	R	R	R	R	R	R	R	R	R	R	R	R
**6**	1	1	1	1	1	1	1	1	1	1	1	1	1	1	1	1	1	1	1	1	1	1	1	1	1	1	1	1	1
**7a**	L	L	L	L	L	L	L	L	L	L	L	L	L	L	L	L	L	L	L	L	L	L	L	L	L	L	L	L	L
**7b**	SC	SC	SC	SC	MC	MC	MC	SC	SC	SC	MC	SC	SC	SC	SC	SC	MC	SC	SC	SC	SC	SC	SC	SC	SC	SC	SC	SC	SC
**7c**	SV	SV	SV	MV	MV	MV	MV	MV	SV	SV	0	SV	0	SV	SV	MV	MV	MV	SV	SV	SV	0	SV	0	MV	MV	SV	SV	MV
**7d**	1	1	1	0	0	1	1	1	1	1	0	1	0	1	1	1	1	1	1	1	1	0	1	0	1	1	1	0	1
**7e**	1	1	1	1	1	1	1	1	1	1	0	1	1	1	1	1	1	1	1	1	1	1	1	1	1	1	1	1	1
**7f**	1	1	1	1	0	0	1	1	1	1	0	0	1	1	1	1	1	1	1	1	1	1	1	1	1	1	1	0	1
**8**	1	1	1	0	1	1	1	1	1	1	1	0	0	0	1	1	1	1	1	0	1	0	1	1	1	1	1	0	1
**9**	0	0	0	P	P	P	P	0	0	P	0	P	0	P	0	P	NP	P	P	P	0	P	P	P	P	P	P	NP	P
**10**	CM	CM	CM	CM	CM	CM	CM	CM	CM	CM	CM	C	0	CM	CM	CM	CM	CM	CM	CM	C	CM	CM	CM	CM	CM	CM	CM	CM

**11**	1	1	1	1	1	1	1	1	1	1	1	1	1	1	1	1	1	1	1	1	1	1	1	1	1	1	1	1	1
**12**	0	0	0	0	0	0	0	1	0	0	1	0	0	0	0	0	0	0	0	1	0	0	0	0	0	0	0	0	0
**13**	0	0	0	0	0	0	0	0	E	0	0	0	0	0	0	0	0	E	0	0	0	0	E	0	E	0	E	0	E
**14**	1	1	1	1	1	1	1	1	1	1	1	1	0	1	1	1	1	1	1	0	1	1	1	1	1	1	1	1	1
**15a**	0	0	0	0	0	0	0	0	0	0	0	0	0	0	0	0	0	0	0	0	0	0	0	0	0	0	0	0	0
**15b**	DI	P	P	DI	FU	FU	P	P	P	P	P	P	P	P	P	P	FU	FU	FU	FU	PFU	FU	FU	FU	P	P	DI	FU	P
**16**	SR	SR	SR	UR	SR	SR	SR	SR	SR	SR	UR	UR	0	A	SR	OC	SR	UR	SR	A	UR	UR	OC	OC	OC	SR	SR	OC	A
**17**	0	0	0	S	S	S	SV	S	SV	SV	0	0	0	0	0	S	S	SV	SV	0	SV	SV	S	S	S	SV	SV	S	SV
**18**	0	0	0	M	M	VM	VM	M	M	V	0	0	0	0	0	VM	VM	VM	VM	0	0	M	M	M	M	M	VM	VM	VM
**19a**	1	1	1	1	1	1	1	1	1	1	1	1	1	1	1	1	1	1	1	1	1	1	1	1	1	1	1	1	1
**19b**	0	1	0	0	0	0	0	0	0	1	0	0	0	0	0	0	0	0	0	0	0	0	1	0	0	0	0	1	0
**19c**	0	1	0	0	1	1	1	0	1	1	1	1	1	1	0	1	1	1	1	1	0	1	1	1	1	1	1	1	1
**20**	0	1	0	0	1	1	1	0	1	0	1	1	1	1	0	1	1	1	1	1	0	1	1	1	1	1	1	1	1
**21**	0	1	0	0	1	1	1	0	1	0	1	1	1	1	0	1	1	1	1	1	0	1	1	1	1	1	1	1	1
**22a**	0	0	0	0	1	1	1	0	1	1	1	1	1	1	0	1	1	1	1	0	0	1	1	1	1	1	1	1	1
**22b**	0	0	0	0	0	0	0	0	0	0	0	0	0	0	0	0	0	0	0	0	0	0	0	0	0	0	0	0	0
**23**	SV	S	SV	0	SV	SV	SV	S	S	SV	S	S	SV	SV	S	SV	SV	SV	SV	SV	S	SV	SV	SV	SV	SV	S	SV	SV
**24**	0	1	0	0	1	1	1	0	1	1	1	1	1	1	0	1	1	1	1	1	0	1	1	1	1	1	1	1	1
**25**	0	0	0	0	1	1	1	0	0	0	1	0	0	0	0	1	1	1	1	1	0	1	1	0	1	1	1	1	1
**26**	0	1	0	0	1	1	1	0	1	0	1	0	1	1	0	1	1	1	1	1	0	1	1	1	1	1	1	1	1
**27, if applicable (N = 14)**	n/a	n/a	n/a	n/a	1	1	n/a	n/a	n/a	n/a	n/a	n/a	0	n/a	0	1	1	1	n/a	n/a	n/a	0	1	n/a	0	0	1	0	1
**28**	1	1	1	1	1	1	1	1	1	1	1	1	1	1	1	1	1	1	1	1	1	1	1	1	1	1	1	1	1
**29**	0	1	1	0	1	1	1	0	1	1	1	0	0	0	0	1	1	1	1	0	1	1	1	1	1	0	1	1	1
**30**	0	0	0	0	0	1	1	0	0	1	0	0	0	0	0	1	1	1	1	0	0	0	0	0	0	0	1	1	1
**31**	0	0	0	0	1	1	1	0	0	0	0	0	0	0	0	1	1	1	0	1	0	0	1	0	0	0	1	1	1
**32**	I	I	I	I	E	E	E	I	I	I	E	I	I	I	I	E	E	E	E	E	I	E	E	I	E	E	E	E	E
**Results**	1	3	1	1	4	5	5	2	2	2	4	0	1	1	3	4	6	4	4	2	2	4	5	1	4	4	5	4	6
**33**	0	1	0	0	0	1	1	1	0	1	1	0	0	0	1	1	1	1	1	0	0	0	1	0	1	1	1	0	1
**34**	1	1	0	1	1	1	1	1	1	0	1	0	1	1	1	1	1	1	1	1	1	1	1	0	1	1	1	1	1
**35a**	0	V	0	0	T	T	T	0	V	V	T	V	V	V	0	T	T	T	T	T	0	T	T	V	T	T	T	T	T

**35b**	0	0	0	0	1	1	1	0	0	0	0	0	0	0	1	0	1	0	0	0	0	0	1	0	0	0	0	1	1
**36**	0	1	1	0	1	1	1	0	1	1	1	0	0	0	0	1	1	1	1	0	1	1	1	1	1	0	1	1	1
**37**	0	0	0	0	0	0	0	0	0	0	0	0	0	0	0	0	1	0	0	0	0	1	0	0	0	1	1	0	1
**Discussion**	2	2	2	2	2	2	2	2	2	2	2	2	2	2	2	2	2	2	2	1	2	2	2	2	2	2	2	2	2
**38**	1	1	1	1	1	1	1	1	1	1	1	1	1	1	1	1	1	1	1	0	1	1	1	1	1	1	1	1	1
**39**	1	1	1	1	1	1	1	1	1	1	1	1	1	1	1	1	1	1	1	1	1	1	1	1	1	1	1	1	1
**Other information**	1	1	1	1	0	0	0	1	0	0	0	0	0	0	1	0	0	0	0	0	0	0	0	0	0	0	0	0	0
**40**	0	0	0	0	0	0	0	0	0	0	0	0	0	0	0	0	0	0	0	0	0	0	0	0	0	0	0	0	0
**41**	0	0	0	0	0	0	0	0	0	0	0	0	0	0	0	0	0	0	0	0	0	0	0	0	0	0	0	0	0
**42**	NF	NF	NF	FR	F	F	F	NF	0	0	0	F	F	F	FR	F	F	F	0	F	F	F	F	F	F	F	0	F	F

Note: CLAIM = Checklist for Artificial Intelligence in Medical Imaging.
Extracted from Mongan J, Moy L, Kahn CE Jr. Checklist for Artificial Intelligence in Medical Imaging (CLAIM): A Guide for Authors and Reviewers. Radiol Artif Intell. 2020 Mar 25;2(2):e200029

Supplementary Table S14 QUADAS-2 assessment per study

Study	Baidya Kayal 201[illegible]	Baidya Kayal 2021	Baidya Kayal 2022	Bailly 2017	Chen 2020A	Chen 2020B	Chen 2021	Cho 2019	Dai 2020	Djuričić 2022	Dufau 2019	Jeong 2019	Kim 2021A	Kim 2021B	Lee 2020	Lin 2020	Liu 2021	Luo 2022	Pereira 2021	Sheen 2019	Song 2019	Wan 2021	Wu 2018	Xu 2019	Xu 2021	Yin 2021	Zhang 2021	Zhao 2019	Zhong 2022
**Risk of bias**																													
**Patient Selection**	U	L	U	U	L	L	L	L	L	L	L	U	H	U	L	L	L	L	L	L	L	U	L	L	L	L	L	U	L
**Index Test**	H	L	H	H	H	L	L	H	L	L	H	L	H	L	H	L	L	L	L	L	H	H	L	H	L	L	L	H	L
**Reference Standard**	H	L	L	H	L	L	L	L	L	L	L	L	L	L	L	L	L	L	L	L	L	L	L	L	L	L	H	L	L
**Flow and Timing**	L	L	U	U	L	U	L	U	U	U	U	U	U	L	U	L	L	U	U	L	L	U	L	U	U	U	L	U	L
**Application concern**																													
**Patient Selection**	L	L	L	L	L	L	L	L	L	L	L	L	U	L	L	L	L	L	L	L	L	L	L	L	L	L	L	L	L
**Index Test**	H	L	H	H	H	L	L	H	L	L	H	L	H	L	H	L	L	L	L	L	H	H	L	H	L	L	L	H	L
**Reference Standard**	H	L	L	H	L	L	L	L	L	L	L	L	L	L	L	L	L	L	L	L	L	L	L	L	L	L	H	L	L

Note: QUADAS-2 = modified Quality Assessment of Diagnostic Accuracy Studies. L = low risk, U = unclear, H = high risk.
Extracted from Whiting PF, Rutjes AW, Westwood Me, et al; QUADAS-2 Group. QUADAS-2: a revised tool for the quality assessment of diagnostic accuracy studies. Ann Intern Med. 2011;155(8):529-536.

Supplementary Table S15 Subgroup analysis of study quality according to study characteristics

Subgroup	Studies, n	Ideal percentage of RQS, mean ± SD	P value	TRIPOD adherence rate, mean ± SD	P value	CLAIM adherence rate, mean ± SD	P value
Overall	29	29.21 ± 12.96%	n/a	59.23 ± 11.50%	n/a	63.72 ± 13.30%	n/a
Journal type							
Imaging	13	30.77 ± 14.81%	0.570	61.81 ± 12.32%	0.295	65.38 ± 14.33%	0.555
Non-imaging	16	27.95 ± 11.59%		57.14 ± 11.22%		62.38 ± 12.72%	
First authorship							
Radiologist	19	30.55 ± 14.04%	0.452	59.40 ± 12.38%	0.921	65.08 ± 14.80%	0.460
Non-radiologist	10	26.67 ± 10.81%		58.93 ± 11.07%		61.15 ± 10.05%	
Imaging Modality							
CT	9	30.56 ± 12.11%	0.291	62.30 ± 9.96%	0.002[a*]	68.59 ± 10.62	0.004[b*]
MRI	14	31.55 + 13.98%		63.27 ± 10.71%		67.03 ± 12.57	
PET	6	21.76 ± 10.60%		45.24 ± 4.33%		48.72 ± 7.46%	
Publication period							
Before	12	22.69 ± 13.18%	0.020[*]	53.57 ± 11.89%	0.026[*]	56.08 ± 11.82%	0.007[*]
After	16	33.82 ± 10.95%		63.24 ± 10.16%		69.12 ± 11.79%	

Note: Post-hoc multiple comparisons were performed using Tukey-Kramer method, the significance threshold is 0.05 for the adjusted P value using Bonferroni method.
* statistical significance.
a. Post-hoc multiple comparisons, CT vs MRI, P = 0.970 CT vs PET, P =0.006, MRI vs PET, P = 0.002.
b. Post-hoc multiple comparisons, CT vs MRI, P = 0.943 CT vs PET, P =0.007 MRI vs PET, P = 0.007.

Supplementary Table S16 Model metrics of studies included in meta-analysis

Study	Imaging modality	Predictor	Reference standard	TP	FP	FN	TN	AUC (95%CI)
Chen 2021	MRI (T1WI+C)	Radiomics + Clinical	Histology	8	2	3	21	0.98
Dufau 2019	MRI (T1WI+C)	Radiomics	Histology	10	1	0	6	0.842 (0.793−0.883)
Zhang 2021	MRI (DCE)	Radiomics + Clinical	RECIST	10	1	2	8	0.95
Zhong 2022	MRI (T2WI)	Radiomics + Clinical	Histology	9	7	2	25	0.793 (0.610-0.975)

Note: Predictive model for response to NAC, performance on validation dataset. P for good responders, N for poor responders; 5 studies, 44/115 events/sample size.

Supplementary Figure S1 Correlation between quality evaluation tools

RQS = ideal percentage of RQS, TRIPOD = TRIPOD adherence rate, CLAIM = CLAIM adherence rate. The correlation was considered as high, if |r|≥0.8; moderate, if 0.5≤|r|<0.8; low, if 0.3≤|r|<0.5; and not correlated if |r|<0.3.

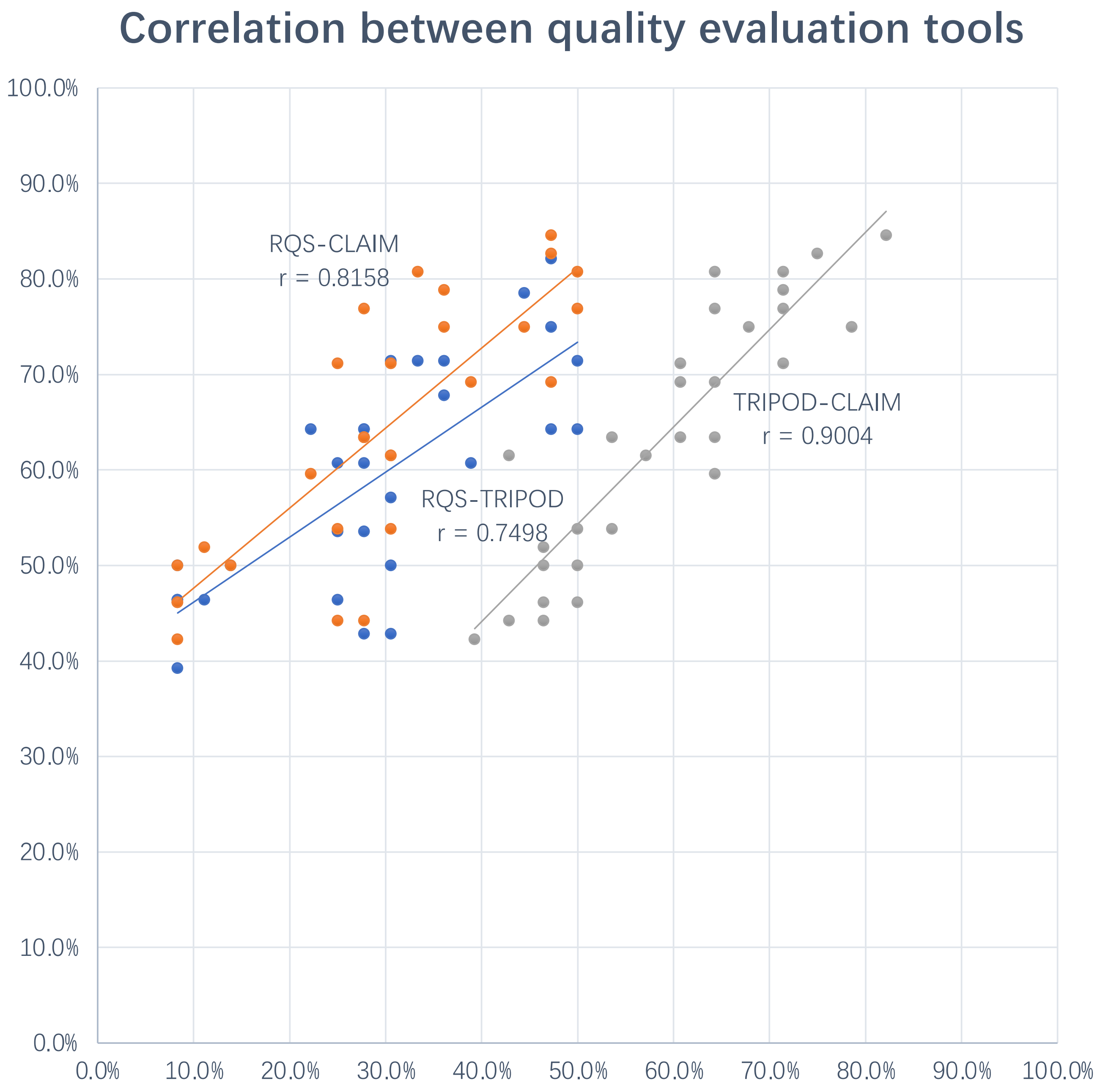

Supplementary Figure S2 Subgroup analysis of quality evaluation results

(A) Published period: P for RQS = 0.020, P for TRIPOD. =0.026, P for CLAIM = 0.007
(B) Imaging modality: P for RQS = 0.291, P for TRIPOD. =0.0.002[a], P for CLAIM = 0.004[b]
(C) First authorship: P for RQS = 0.452, P for TRIPOD. =0.921, P for CLAIM = 0.460
(D) Journal type: P for RQS = 0.570, P for TRIPOD. =0.295, P for CLAIM = 0.555
a. Post-hoc multiple comparisons, CT vs MRI, P = 0.970 CT vs PET, P =0.006, MRI vs PET, P = 0.002.
b. Post-hoc multiple comparisons, CT vs MRI, P = 0.943 CT vs PET, P =0.007 MRI vs PET, P = 0.007.

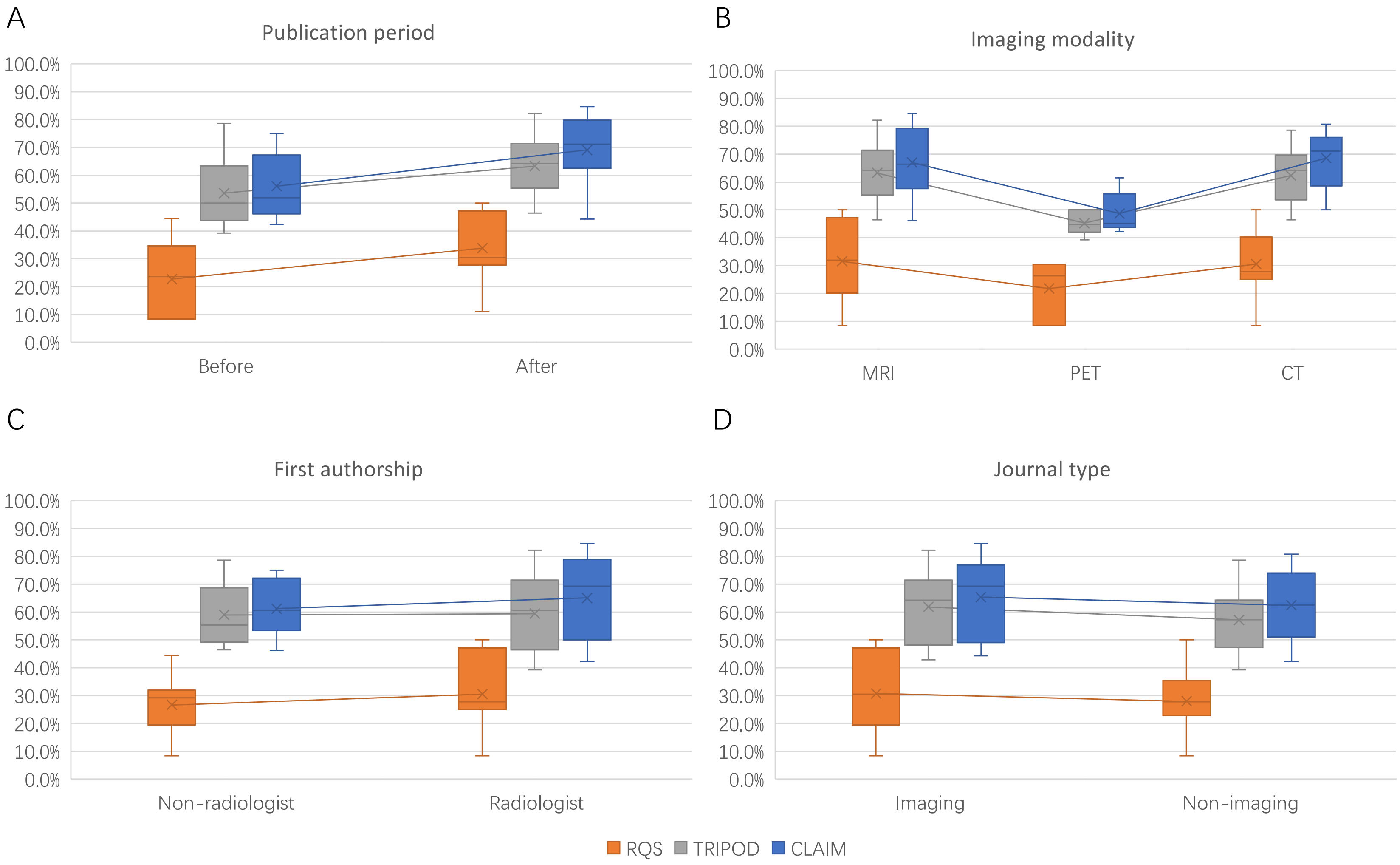
A
Publication period
100.0%
90.0%
80.0%
70.0%
60.0%
50.0%
40.0%
30.0%
20.0%
10.0%
0.0%
Before
After
B
Imaging modality
MRI
PET
CT
C
First authorship
Non-radiologist
Radiologist
D
Journal type
Imaging
Non-imaging
RQS
TRIPOD
CLAIM

Supplementary Figure S3 Forrest plot of pooled sensitivity

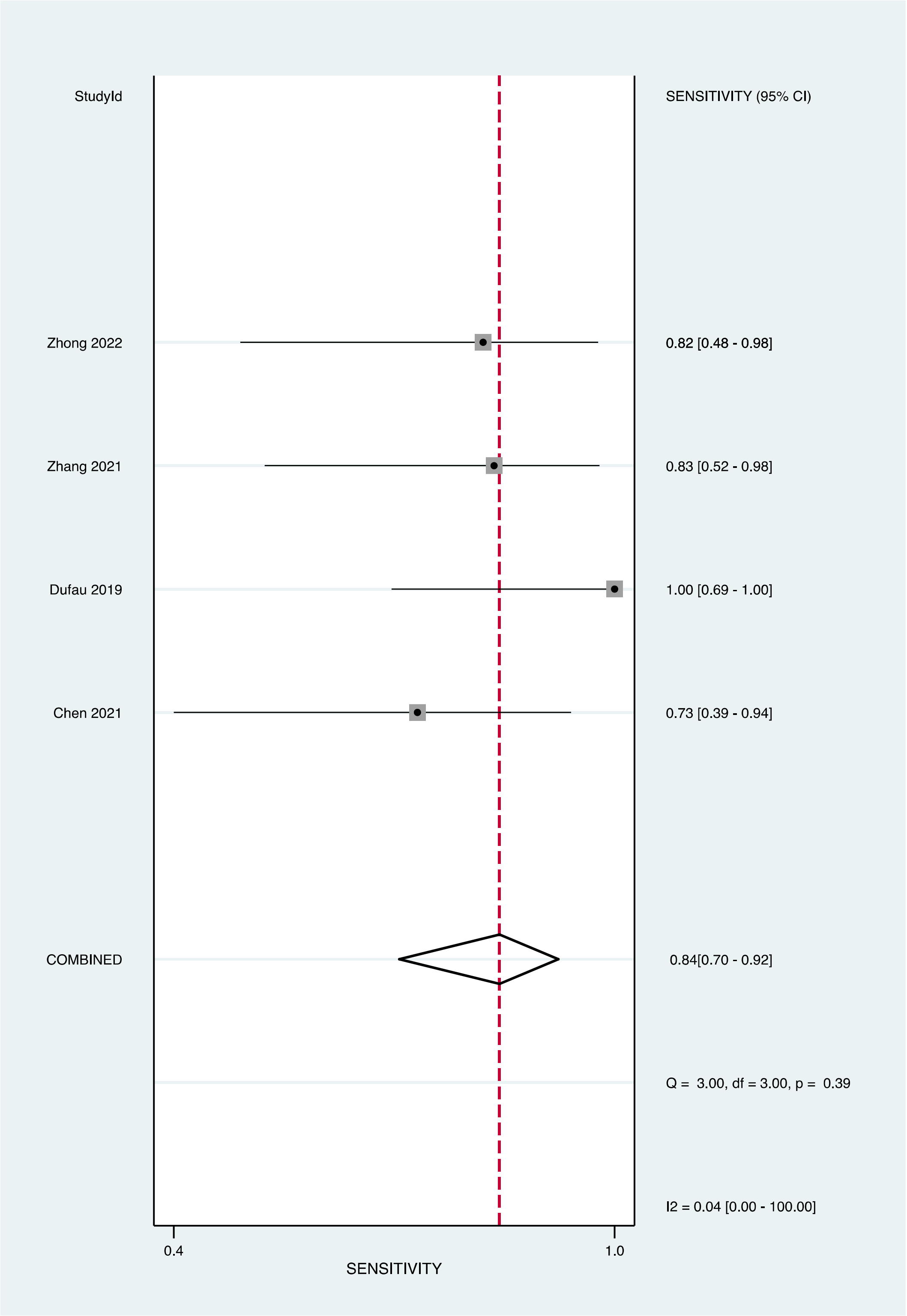

Supplementary Figure S4 Forrest plot of pooled specificity

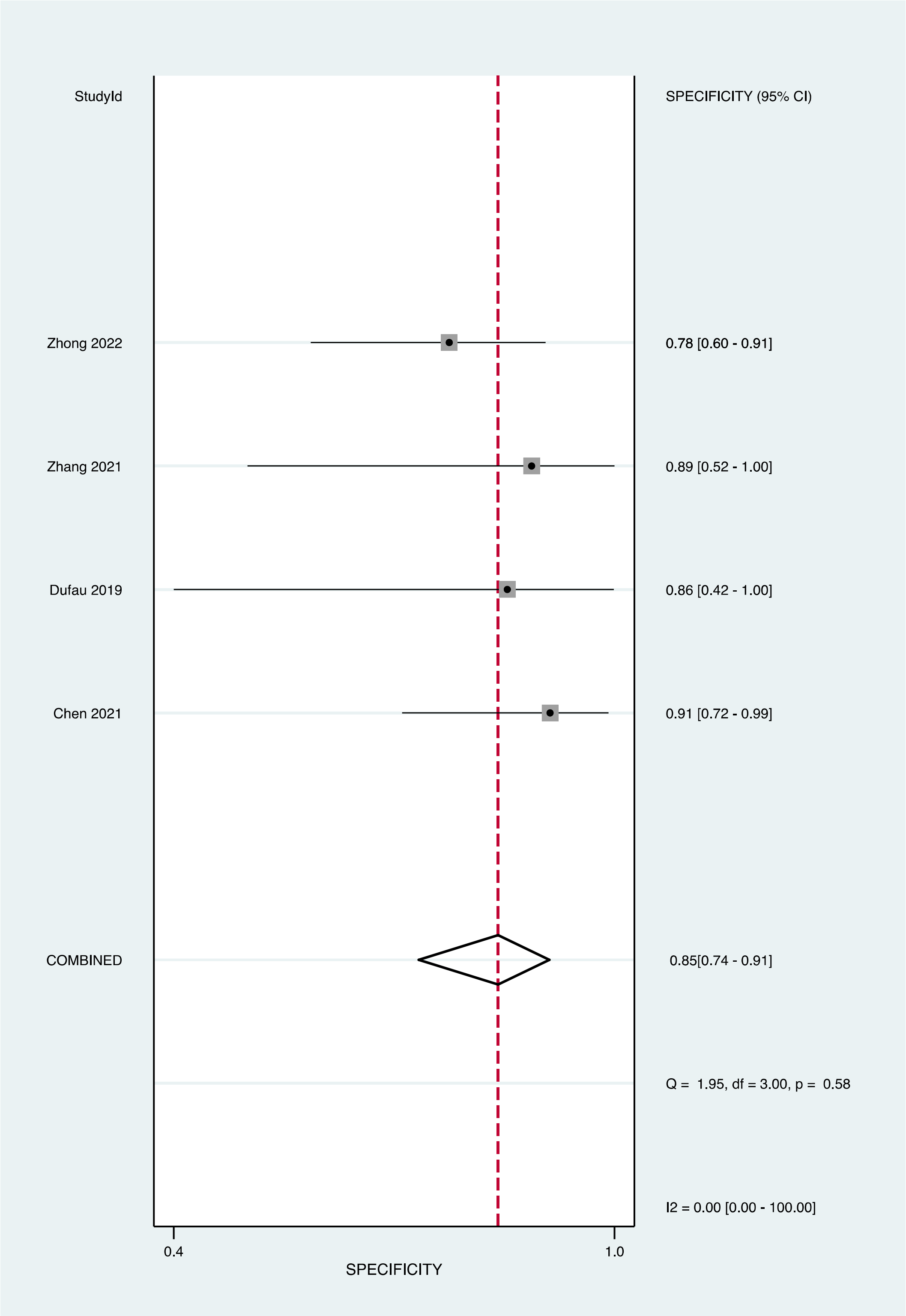

Supplementary Figure S5 Forrest plot of pooled positive likelihood ratio

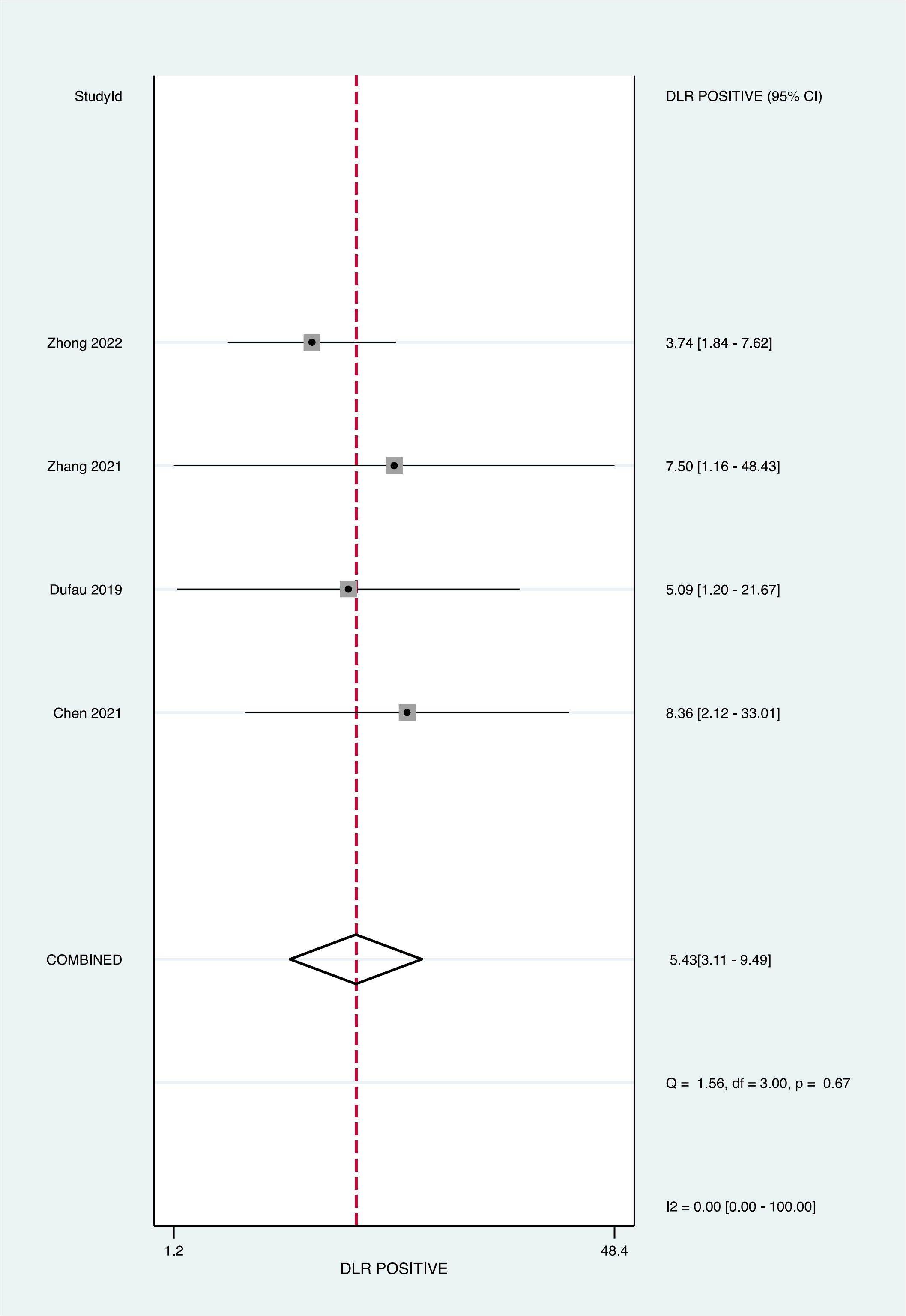

Supplementary Figure S6 Forrest plot of pooled negative likelihood ratio

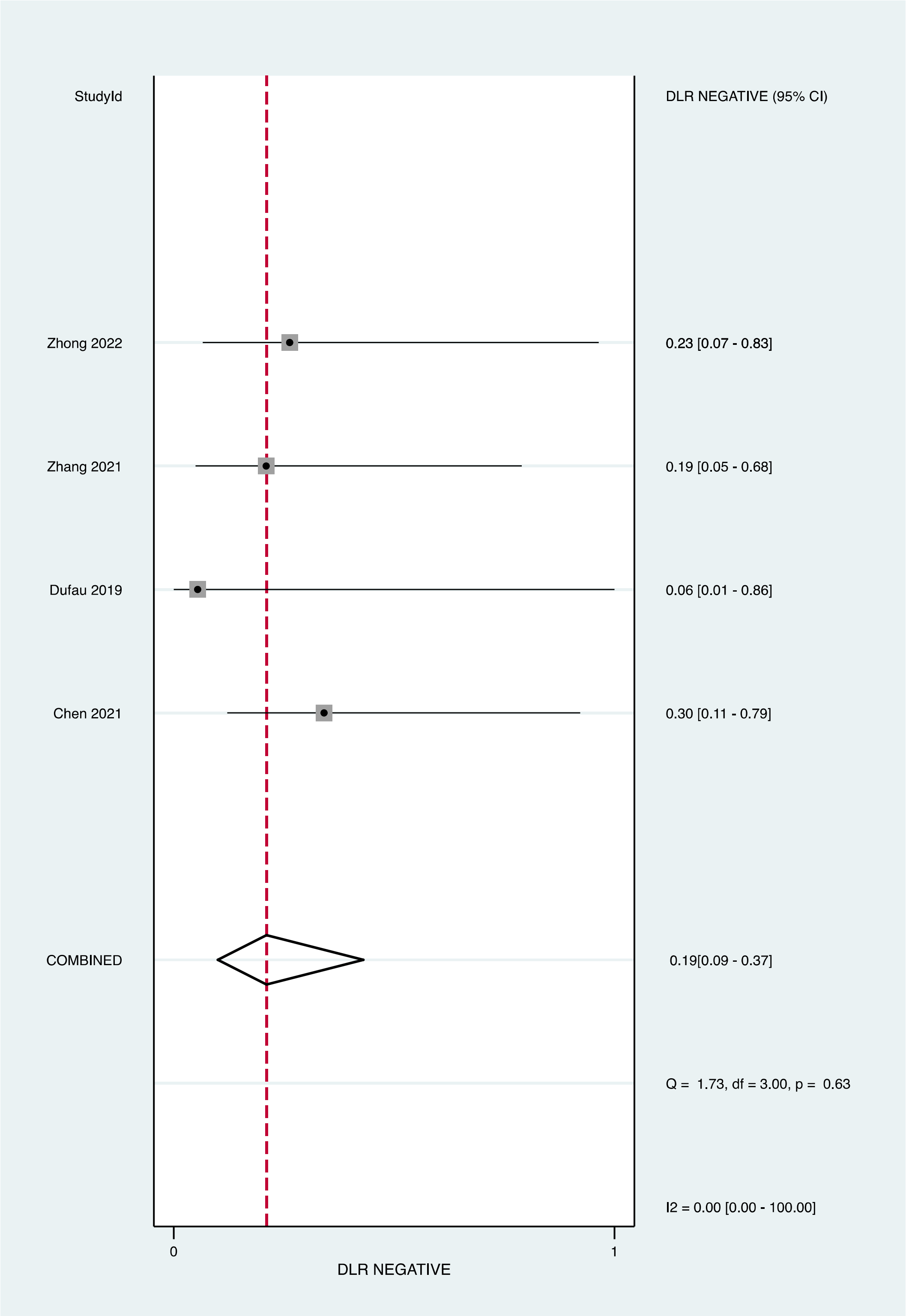

Supplementary Figure S7 SROC curve of the model performance

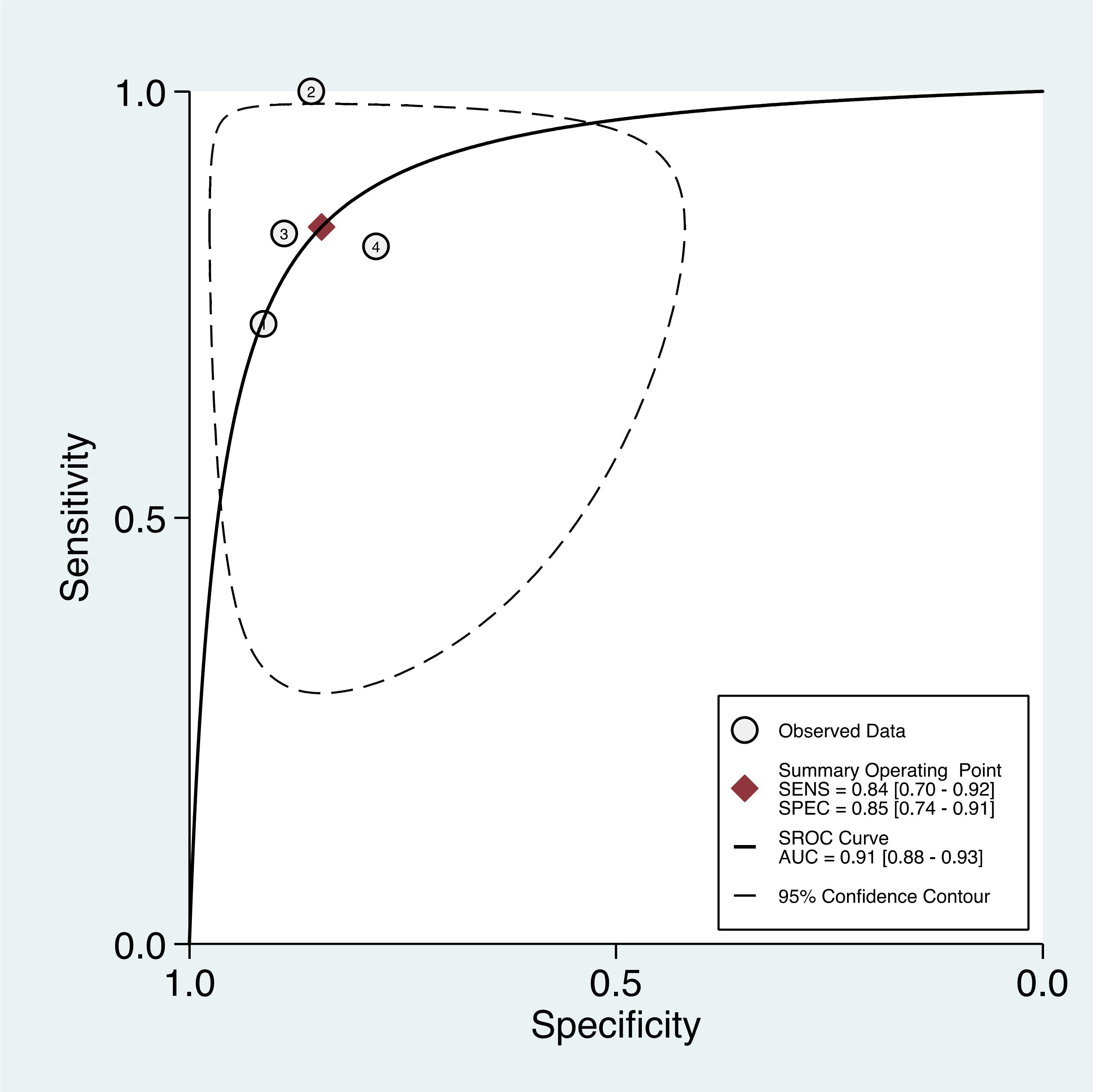

Supplementary Figure S8 Funnel plot of studies included in meta-analysis

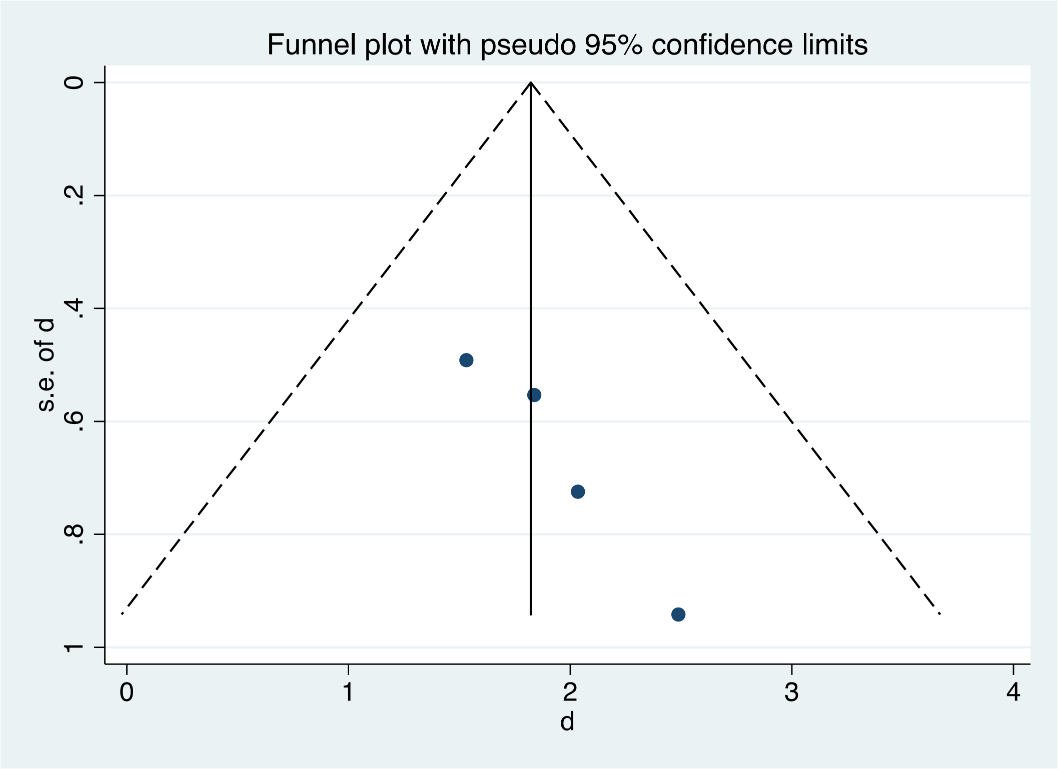

Supplementary Figure S9 Deeks funnel plot of studies included in meta-analysis

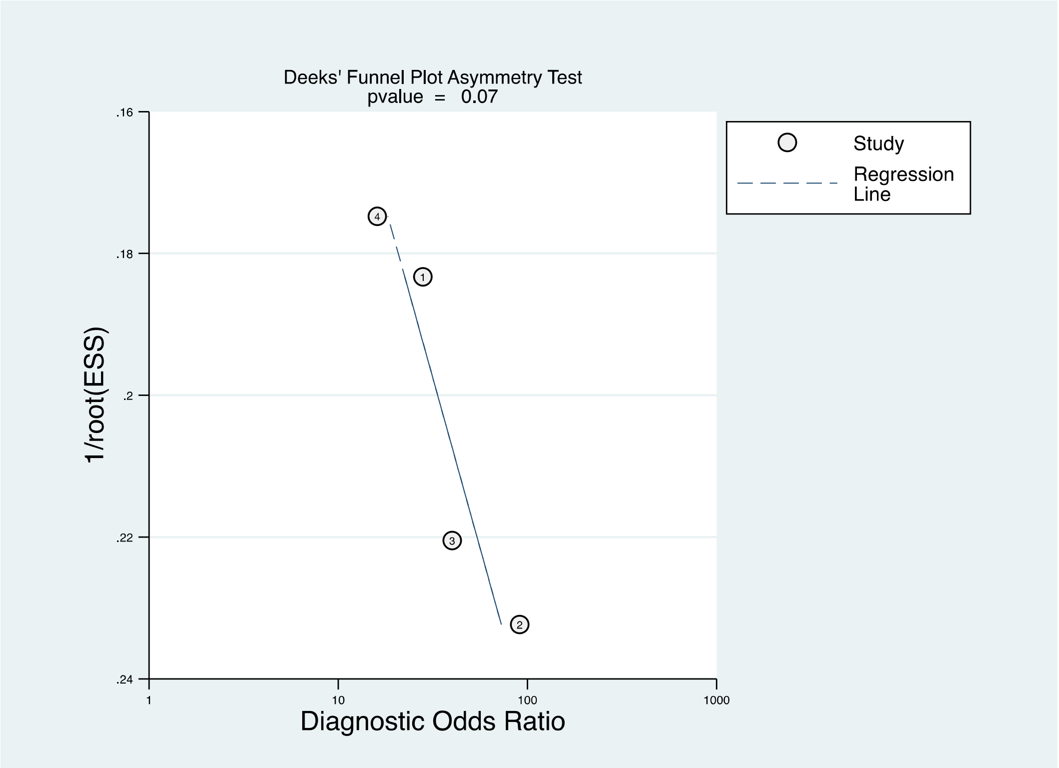

Supplementary Figure S10 Trim and fill analysis of studies included in meta-analysis

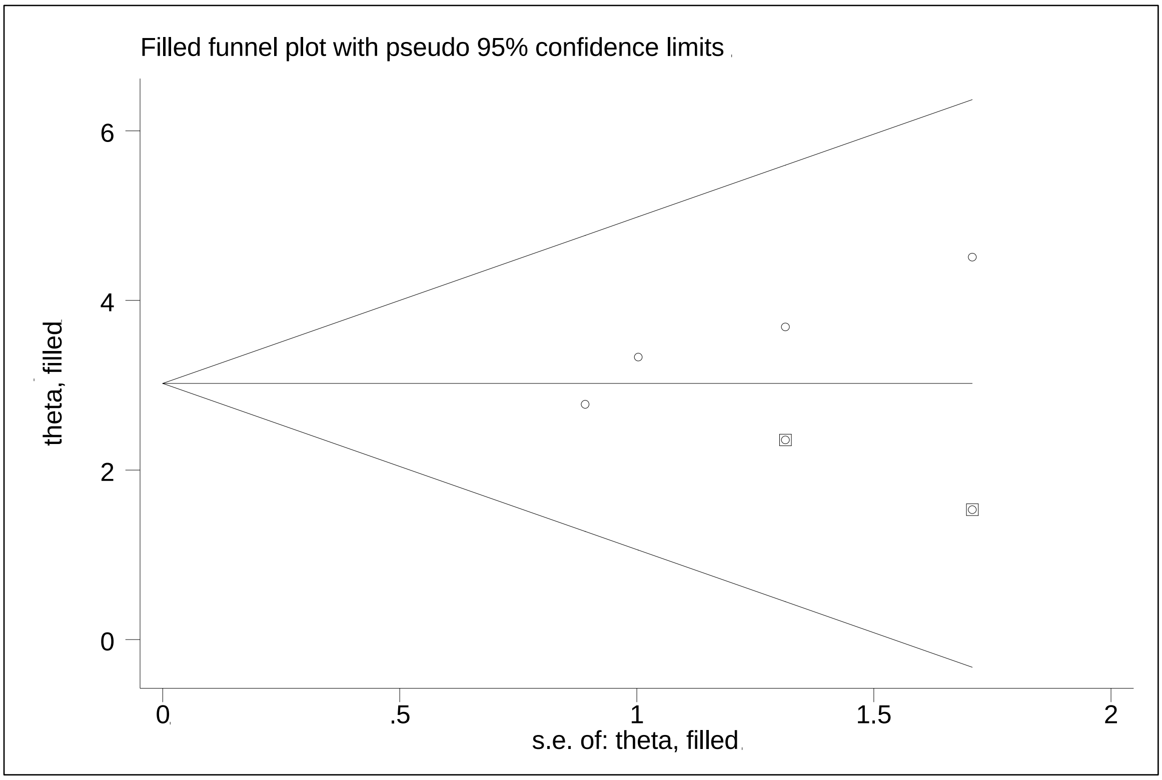

Supplement: Supplementary file 1 — Additional file 1. Note S1: Review protocol. Note S2: Search strategy and study selection. Note S3: Consensus reached during data extraction and quality assessment. Note S4: Data synthesis and analysis methods. Table S1: Data extraction sheet. Table S2: RQS elements according to six key domains. Table S3: TRIPOD reporting completeness checklist. Table S4: CLAIM for authors and reviewers. Table S5: QUADAS-2 tool for risk of bias and concern on application. Table S6: Category of five levels of supporting evidence of meta-analyzes. Table S7: Study characteristics of included studies. Table S8: PICOT of included studies. Table S9: Radiomics methodological issue of included studies. Table S10: Model presentation and performance metrics of included studies. Table S11: RQS rating per study. Table S12: TRIPOD adherence per study. Table S13: CLAIM adherence per study. Table S14: QUADAS-2 assessment per study. Table S15: Subgroup analysis of study quality according to study characteristics. Table S16: Model metrics of studies included in meta-analysis. Figure S1: Correlation between quality evaluation tools. Figure S2: Subgroup analysis of quality evaluation results. Figure S3: Forrest plot of pooled sensitivity. Figure S4: Forrest plot of pooled specificity. Figure S5: Forrest plot of pooled positive likelihood ratio. Figure S6: Forrest plot of pooled negative likelihood ratio. Figure S7: SROC curve of the model performance. Figure S8: Funnel plot of studies included in meta-analysis. Figure S9: Deeks' funnel plot of studies included in meta-analysis. Figure S10: Trim and fill analysis of studies included in meta-analysis. [file 13244_2022_1277_MOESM1_ESM.pdf]
